# Bulky Terphenyl Phosphines Stabilize Otherwise Highly Reactive Iridium Fragments, Key in C–H Activation Reactions

**DOI:** 10.1021/acs.inorgchem.6c01943

**Published:** 2026-07-10

**Authors:** Alejandra Pita-Milleiro, Martina Landrini, Miquel Navarro, Juan J. Moreno, Jefferson Guzmán, Celia Maya, Jesús Campos

**Affiliations:** 16778 Instituto de Investigaciones Químicas (IIQ), Consejo Superior de Investigaciones Científicas (CSIC) and Universidad de Sevilla, Avenida Américo Vespucio 49, Sevilla 41092, Spain

## Abstract

The photogenerated Ir­(I) compound [Cp*Ir­(PMe_3_)] and its Ir­(III) derivative [Cp*IrMe­(PMe_3_)­(solvent)]^+^ [Cp* = η^5^-(C_5_Me_5_)] have tamed the field of C–H bond activation chemistry; however, analogues using bulkier phosphines have been barely investigated. We report the synthesis and characterization of their congested versions using sterically demanding terphenyl (C_6_H_3_-2,6-Ar_2_) phosphine ligands. Two Ir­(I) species, [Cp*Ir­(PMe_2_^ArDtbp2^)] (**3**, Ar^Dtbp2^ = C_6_H_3_-2,6-(C_6_H_3_-3,5-^*t*^Bu_2_)_2_) and [Cp*Ir­(PMe_2_^ArDipp2^)] (**3′**, Ar^Dipp2^ = C_6_H_3_-2,6-(C_6_H_3_-2,6-^*i*^Pr_2_)_2_), were isolated and structurally authenticated, revealing strong metal–arene interactions that confer remarkable stability to these otherwise highly unsaturated species. Despite their apparent similarity, these complexes exhibit sharply contrasting reactivity toward methyl triflate: only the PMe_2_Ar^Dtbp2^ derivative undergoes clean formal oxidative addition to form the targeted cationic methyl complex [Cp*IrMe­(PMe_2_^ArDtbp2^)]^+^ (**4**). Computational studies rationalize this divergent behavior in terms of steric effects beyond conventional metrics. Besides, compound **4** displays rich chemistry, including intramolecular activation of a *tert*-butyl group and selective intermolecular functionalization of 1,2-difluorobenzene, as well as reactions with H_2_ and phenylsilane. These findings evince the role of terphenyl phosphines in stabilizing reactive iridium fragments and highlight their potential for selective C–H activation under sterically congested environments.

## Graphical abstract

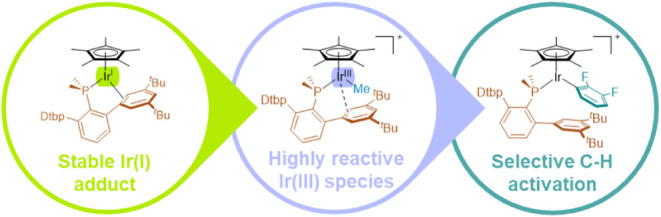

## Introduction

The efficient activation of C–H bonds in alkanes and other common organic compounds remains a significant challenge,^1^ considering its practical implications in both industrial and laboratory syntheses. The most prevalent approach for the efficient activation of C–H bonds involves the use of transition metals. Among all reported systems, iridium complexes based on the motif [Cp*Ir­(PR_3_)] (Cp* = (η^5^-C_5_Me_5_)) occupy a prominent position. The pioneering work of Bergman^2^ with the photogenerated Ir­(I) species [Cp*Ir­(PMe_3_)] demonstrated that intermolecular alkane C–H activation via oxidative addition was indeed possible (Figure 1, “Ir­(I) route”), as also evidenced by Graham in an independent study based on the similar [Cp*Ir­(CO)] fragment.^3^ The later isolation of the related cationic Ir­(III) complex [Cp*Ir­(Me)­(PMe_3_)­(ClCH_2_Cl)]^+^ constituted another seminal discovery by Bergman, exhibiting an outstanding C–H bond activating capacity under very mild conditions (Figure 1, “Ir­(III) route”).^4^

**Figure 1:**
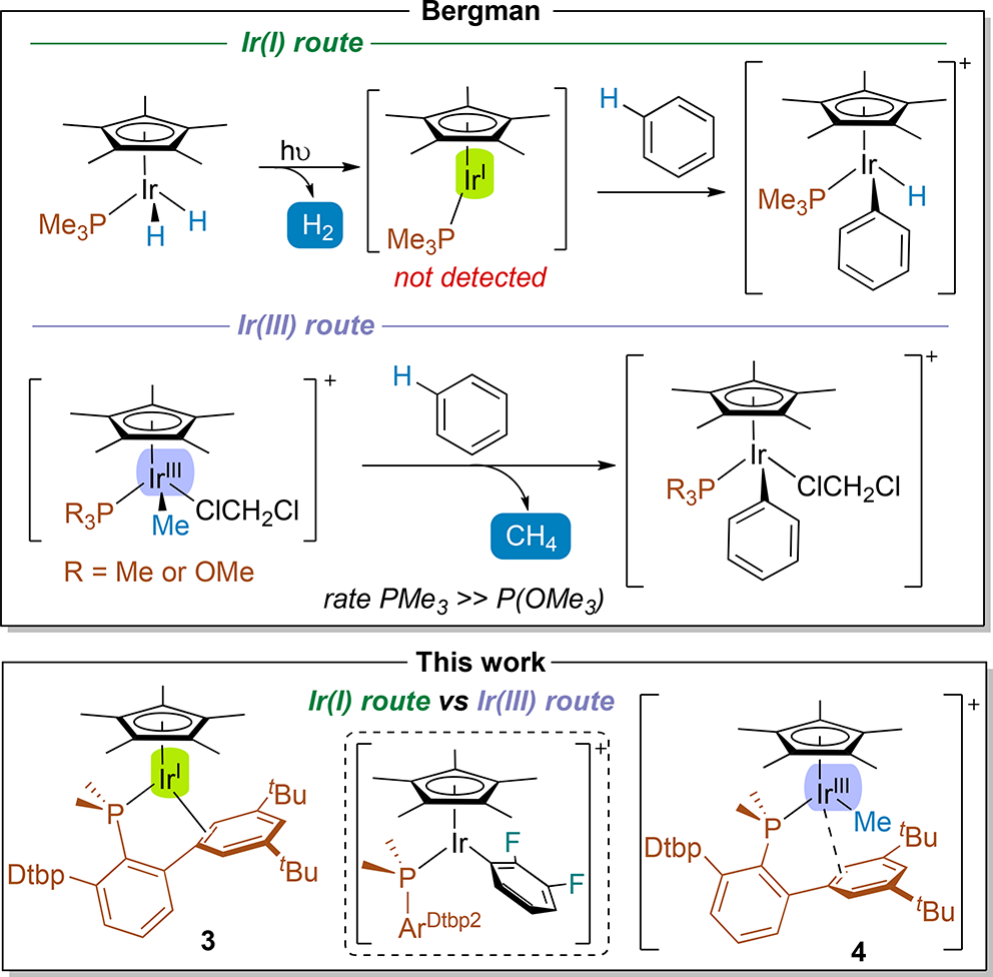
Seminal work by Bergman on the activation of C–H bonds via photogeneration of low-coordinate Ir­(I) species [Cp*Ir­(PMe_3_)] or with the Ir­(III) cation [Cp*Ir­(PMe_3_)­(Me)]^+^, and the bulky Ir­(I) and Ir­(III) versions developed in this work, highlighting the C–H bond activation of 1,2-difluorobenzene.

Since then, numerous neutral and cationic Rh and Ir complexes featuring the [Cp*M­(PR_3_)] (M = Rh, Ir) framework have been disclosed,^5^ and their proficiency in C–H functionalization catalysis^6^ has been widely investigated. Nonetheless, only a single similar example of the iconic Ir­(III) complex [Cp*Ir­(Me)­(PR_3_)­(solvent)]^+^ has been reported, in which the trimethylphosphine ligand is replaced by phosphite P­(OMe)_3_. Interestingly, the resulting complex [Cp*Ir­(Me)­(P­(OMe)_3_)­(ClCH_2_Cl)]^+^ shows up to 30-fold reduced reactivity toward C–H activation, likely due to the lower electron density at the metal center.^5^ These results underscore the potential for fine-tuning the reactivity of this highly active fragment through phosphine ligand modification. However, this intrinsic reactivity has as well hindered further modifications, as intramolecular C–H activation reactions have typically led to cyclometalation, thereby diminishing or fully quenching intermolecular reactivity.^7^

We recently approached this synthetic challenge by using the bulky terphenyl phosphine PMe_2_Ar^Dipp2^ (Ar^Dipp2^ = C_6_H_3_-2,6-(C_6_H_3_-2,6-^*i*^Pr_2_)_2_), envisioning that its use would present several advantages. First, it would circumvent undesired cyclometalation events by blocking the *ortho-*positions of the central aryl substituent. Second, it would foster a secondary interaction between the metal center and one of the flanking aryl rings, providing thermal stability while maintaining great reactivity toward C–H bond activation.^8^ Besides, the stabilizing weak interactions could also facilitate access to a bulkier version of the highly reactive Ir­(I) species [Cp*Ir­(PMe_3_)] transiently photogenerated by Bergman,^2^ but never isolated or spectroscopically detected (Figure 1, “Ir­(I) route”).

In a previous study, we prepared compound [Cp*Ir­(PMe_2_Ar^Dipp2^)­Cl]­[BAr^F^] ([BAr^F^] = tetrakis­[3,5-bis­(trifluoromethyl)­phenyl]),^9^ a species ideally suited to access the targeted derivative of the Bergman’s Ir­(III) cationic fragment by reaction with LiMe or MeMgBr. However, contrary to our expectations, these reactions did not result in the anticipated methylation. Instead, they revealed an interesting reactivity pattern in which either the Cp* or the phosphine ligand where directly involved in the resulting transformations.^10^ To circumvent this, we have now targeted the reduced Ir­(I) compounds of formula [Cp*Ir­(PR_3_)] using not only PMe_2_Ar^Dipp2^, but also the related terphenyl phosphine PMe_2_Ar^Dtbp2^ (Ar^Dtbp2^ = C_6_H_3_-2,6-(C_6_H_3_-3,5-^*t*^Bu_2_)_2_)^8b^ (Figure 1). Their reactivity toward MeOTf (OTf^–^ = trifluoromethanesulfonate) en route to the cationic Ir­(III) versions has been studied, finding a distinctively diverging behavior for the two phosphines. Besides, the intramolecular and intermolecular C–H bond activation reactivity of the resulting cationic [Cp*IrMe­(PMe_2_Ar^Dtbp2^)]^+^ complex has been investigated.

## Results and Discussion

### Synthesis of Unsaturated Ir­(I) Compounds **3** and **3′**

At first, we treated dimer [Cp*IrCl_2_]_2_ with an equimolar amount of PMe_2_Ar^Dtbp2^ in CH_2_Cl_2_, resulting in an immediate color change to a bright orange-red solution. After stirring for 30 min at room temperature, compound [Cp*IrCl_2_(PMe_2_Ar^Dtbp2^)] (**1**) was isolated in 92% yield (Scheme 1). Interestingly, phosphine PMe_2_Ar^Dipp2^ exhibits a slightly more sterically hindered profile in close proximity to the metal center^11^ and, as a result, it does not react with [Cp*IrCl_2_]_2_, which prevented us from isolating the analogous neutral dichloride Ir­(III) species. In our prior work, we observed that for phosphine PMe_2_Ar^Dipp2^, coordination to iridium only takes place in the presence of one equivalent of NaBAr^F^,^9^ leading to the isolation of the cationic complex [Cp*IrCl­(PMe_2_Ar^Dipp2^)]­[BAr^F^] (**2′**; the apostrophe will denote compounds based on PMe_2_Ar^Dipp2^). Similarly, the related cationic complex **2**, based on PMe_2_Ar^Dtbp2^, could be isolated by the analogous procedure or, alternatively, by treating **1** with one equivalent of Na­[BAr^F^], in both cases in almost quantitative yield (see Supporting Information for details).

**Scheme 1:**
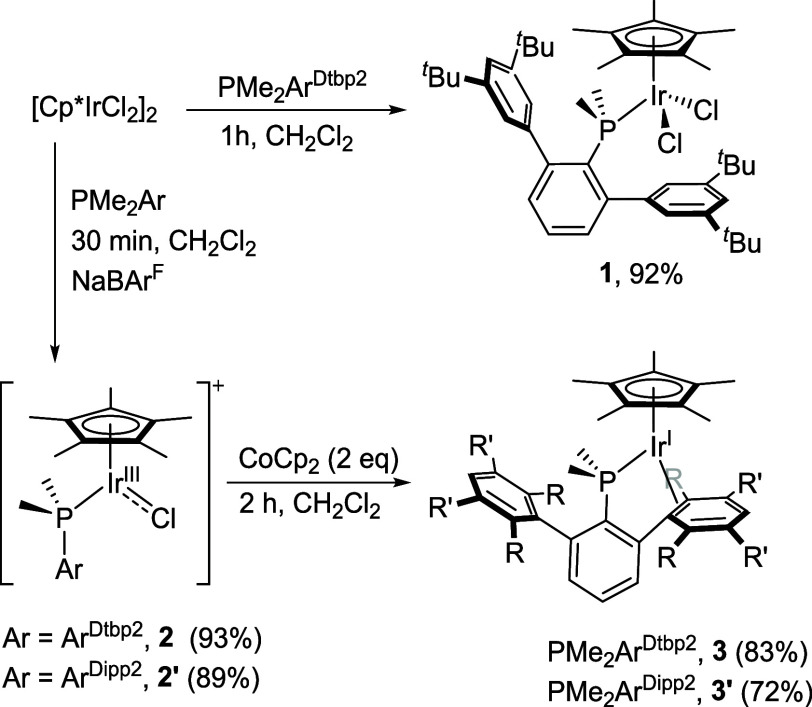
Synthesis of Compounds **1**, **2** and **3**

Compounds **1** and **2** were characterized by multinuclear NMR spectroscopy, exhibiting quite distinct ^31^P­{^1^H} NMR resonances at −19.1 and 8.9 ppm, respectively, the latter similar to that found for **2′** (c.f. 6.6 ppm). Their structures were also authenticated by X-ray diffraction studies, confirming their piano-stool conformations (Figure 2, top). A slight contraction of the Ir–P bond length upon chloride release from **1** (2.315(1) Å) to **2** (2.292(1) Å) is consistent with the more electrophilic character of the metal center. The Ir–Cl bond distances are comparable in both compounds (2.40 Å average for **1** and 2.429(1) Å for **2**), which contrasts with the reduced length measured for **2′** (2.278(1) Å) that we previously attributed to the π-donor capacity of the chloride ligand in a formally 16-electron complex. At variance, the reduced steric profile of PMe_2_Ar^Dtbp2^ in proximity to the iridium atom allows to compensate unsaturation by a weak interaction with one of the flanking aryl rings (Ir–C13, 2.463(4); Ir–C14, 2.494(4) Å), while for **2′** the shortest Ir···C_arene_ distances are 3.124 and 3.160 Å.

**Figure 2:**
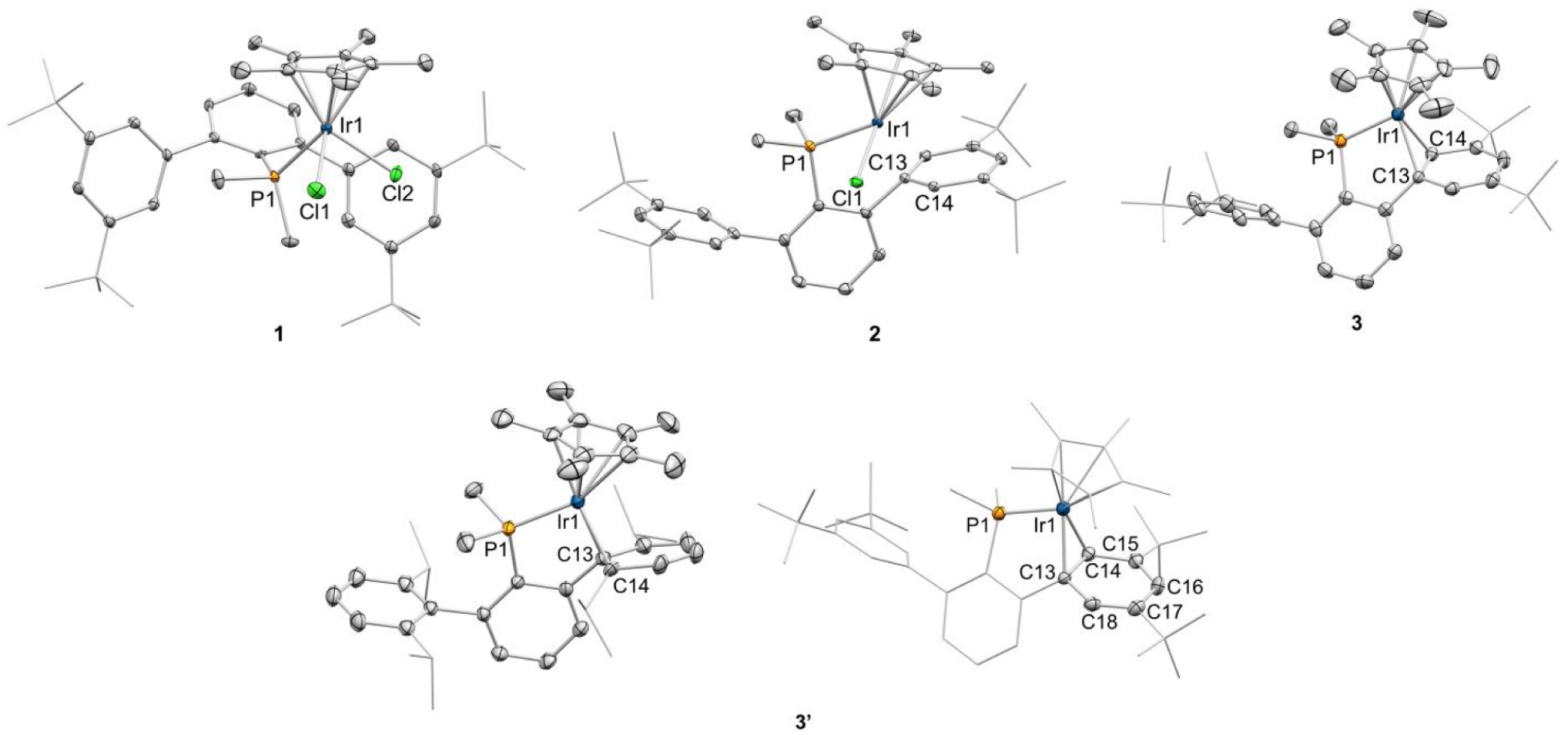
ORTEP diagram of complexes **1**, **2**, **3** (top) and **3′** (bottom; two views of the same crystallographic structure). For cationic complexes, [BAr^F^]^−^ counterion is omitted for clarity. All hydrogen atoms are excluded for clarity and thermal ellipsoids are set at 50% probability. Wireframe is used to represent the *iso*-propyl and *tert*-butyl groups.

Next, we attempted the preparation of the corresponding unsaturated Ir­(I) compounds analogous to the transiently photogenerated Bergman’s [Cp*Ir­(PMe_3_)] fragment.^2^ To our delight, both complexes, **2** and **2′**, could be chemically reduced by reaction with 2 eq. of CoCp_2_ in dichloromethane to form the Ir­(I) species **3** and **3′** (Scheme 1), characterized by a downshift of their corresponding ^31^P­{^1^H} signals to −1.6 and 3.4 ppm, respectively. Intriguingly, the ^1^H NMR spectrum of complex **3** revealed a doublet (^2^ *J*_HP_ = 8.3 Hz) at 3.08 ppm that could be assigned to the proton in the *ortho*-position of one of the lateral rings of the phosphine ligand. This is an extremely low-frequency chemical shift for a supposedly aromatic proton, which results from a strong interaction between the metal center and the aryl ring. This is also supported by the corresponding ^13^C­{^1^H} NMR spectrum, where equally low-frequency resonances for the *ipso*- and one of the *ortho*-carbons were recorded at 50.4 and 41.2 ppm, respectively. Similarly, the ^13^C­{^1^H} NMR spectrum of complex **3′** exhibits two resonances at 61.3 and 51.9 ppm for the *ipso*- and *ortho*- carbons of the aryl ring featuring an interaction with the metal center, once more clearly down-shifted from normal values (c.f. around 130–150 ppm for the noninteracting rings in compounds **1–2**).

Single-crystals of **3** and **3′** were also investigated by X-ray crystallography (Figure 2, top and bottom respectively), confirming the strong bonding between the metal center and of one of the lateral rings of the phosphine. This is reflected by short Ir–C bond distances (**3**: Ir–C13 = 2.127(3), Ir–C14 = 2.145(3) Å; **3′**: Ir–C14 = 2.157(5), Ir–C13 = 2.143(5) Å) that are comparable to the sum of the corresponding covalent radii (2.14 Å).^12^ Besides, the strong interaction restraints conjugation within the ring, as inferred from two shorter (C17–C18, 1.337(5); C15–C16, 1.352(5) Å) and three longer (C14–C15, 1.455(5); C16–C17, 1.442(5); C13–C18, 1.471(5) Å) C–C bond distances, differing from the narrow range of lengths of the noninteracting ring (1.385(5)–1.397(5) Å). The interacting C13–C14 fragment also exhibits a longer bonding distance of 1.476(5) Å, consistent with the expected back-donation from the metal to an antibonding orbital. These results indicate that the aryl ring is indeed donating electron density to the otherwise highly unsaturated metal complex.

The fact that the aryl ring interacts with the metal center is also supported by topological studies: complexes **3′** and **3** feature a bond critical point (bcp) involving the Ir center and the C_*ipso*_ and a bcp involving the Ir and the C_*ortho*_ of one of the lateral rings (Figure 3 and Figures S39 and S40). Overall, the interactions with the lateral aryl rings in **3** and **3′** resemble more the strong coordination of an olefin rather than a weak interaction as found in the Ir­(III) cation **2** and **2′** and other low-coordinate complexes stabilized by terphenyl phosphines.^8,10,11^

**Figure 3:**
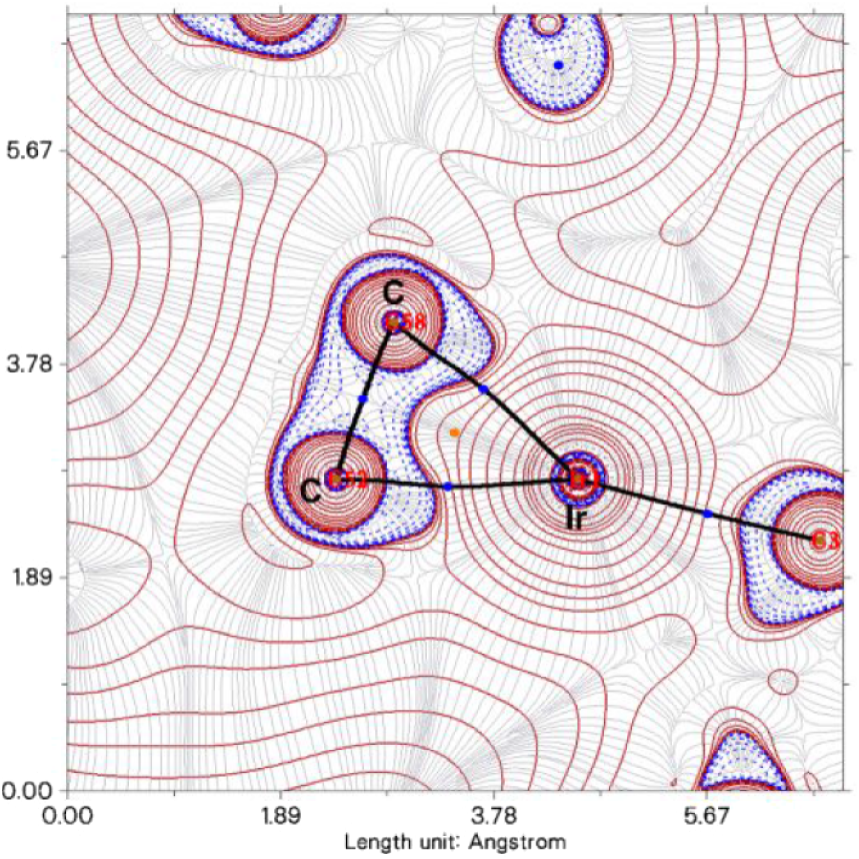
Plot of the Laplacian of the electron density, ∇^2^ρ, of complex **3** in the plane containing the Ir, C*_ipso_*, and one of the C*_ortho_* atoms. Both C atoms belong to the Dtbp group which features an interaction with the metal center.

### Synthesis of Cationic Ir­(III)-Methyl Complexes

With compounds **3** and **3′** in hand, we attempted the preparation of the desired bulkier versions of the Bergman’s cationic methyl complex [Cp*IrMe­(PMe_3_)­(CH_2_Cl_2_)]^+^. Contrary to our expectations, the two apparently analogous Ir­(I) complexes **3** and **3′** showed dissimilar reactivity toward MeOTf. While **3** reacted rapidly even at −20 °C with the carbon electrophile to afford cleanly the desired Ir­(III) cationic complex [Cp*IrMe­(PMe_2_Ar^Dtbp2^)]^+^ (**4**, Scheme 2), with OTf^–^ as counteranion, the PMe_2_Ar^Dipp2^ analogue revealed no reactivity even after heating at 80 °C for 24 h. The formation of **4** was confirmed by ^31^P­{^1^H} NMR spectroscopy, with a distinctive shift to 8.5 ppm (c.f. −1.6 ppm for **3**). ^1^H NMR spectroscopy confirmed the incorporation of a methyl group on the metal center, featuring a doublet with intensity corresponding to 3H at 0.56 ppm (^3^ *J*_HP_ = 7.2 Hz). In turn, the corresponding carbon resonates as a doublet at −15.5 ppm (^2^ *J*_CP_ = 14 Hz).

**Scheme 2:**
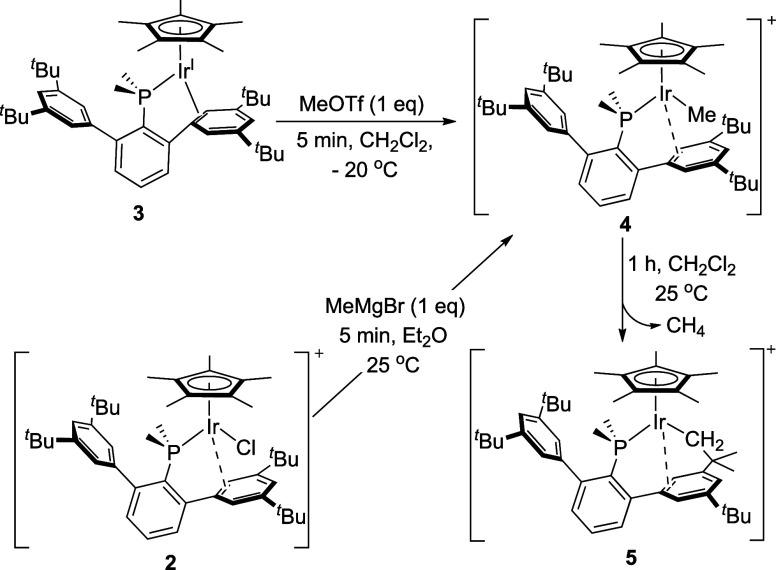
Synthesis of Ir­(III) Compound **4** from Precursors **3** or **2** and Intramolecular C–H Bond Activation toward **5**

Density Functional Theory (DFT) studies confirmed the dissimilar reactivity of complexes **3** versus **3′**. While for both species the formal oxidative addition of MeOTf is thermodynamically favored (**3**, Δ*G* = −17.8 kcal·mol^–1^; **3′**, Δ*G* = −13.2 kcal·mol^–1^), the affordable energy barrier calculated for **3** (Δ*G* ^‡^ = 18.6 kcal·mol^–1^) contrasts with the inaccessible barrier for **3′** (Δ*G* ^‡^ = 35.6 kcal·mol^–1^). Notably, in both systems the methyl group approaches the iridium center to form the Ir–C bond while the metal simultaneously preserves its stabilizing interaction with the flanking aryl ring; yet only for **3** does this trajectory lead to an energetically feasible formal oxidative addition (see Figure 4). We explored this difference in reactivity through Activation Strain Analysis,^13^ which exposes that the elevated difference between the energy barriers is explained by the steric hindrance of the *iso-*propyl groups in the positions 2- and 6- of the flanking aryl rings which prevents the formation of the corresponding Ir–Me species for the Dipp phosphine (see Section 3.7 of the SI for more details). It is worth mentioning that this represents a clear example in which common parameters used to gauge ligand steric profiles do not fully align with experimental observations. Thus, while both PMe_2_Ar^Dtbp2^ and PMe_2_Ar^Dipp2^ phosphines exhibit similar buried volumes (%V_bur_ of 35.5 and 39.3, respectively; experimentally determined for IrCl­(CO)_2_(PMe_2_Ar)),^14^ the reactivity of **3** and **3′** clearly differentiates.

**Figure 4:**
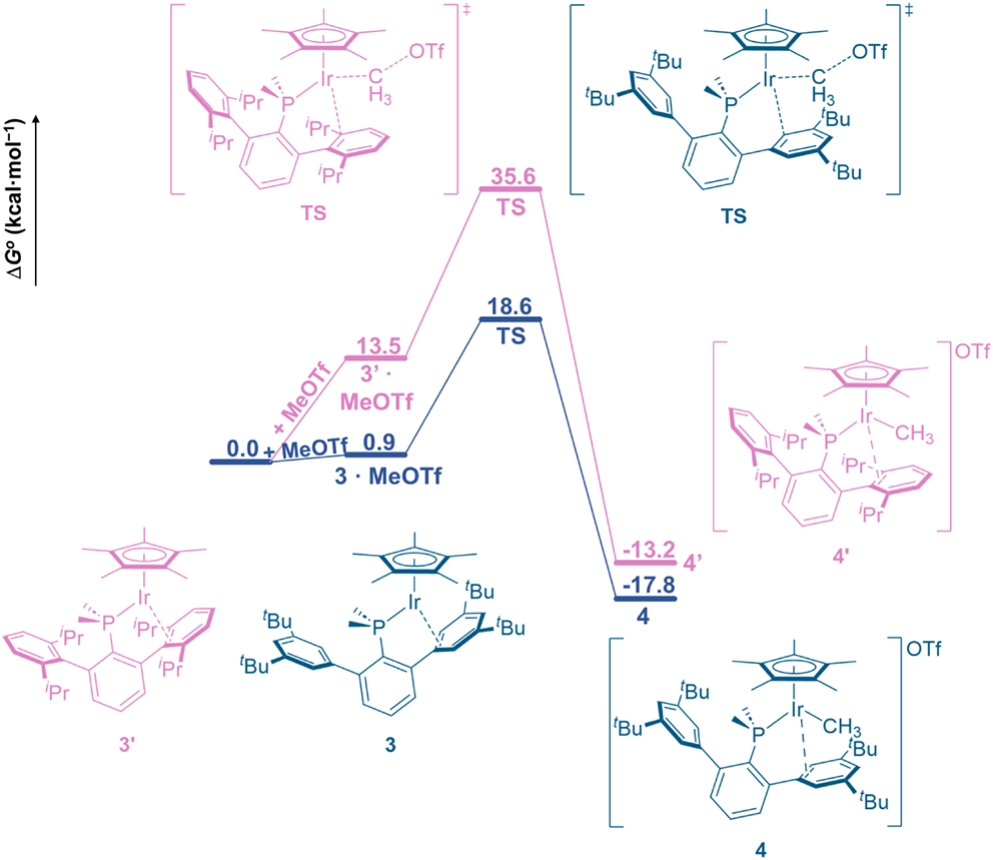
Free energy profile of the proposed mechanism for the formation of **4** and **4′** in blue and pink, respectively.

An alternative, more obvious route, toward compounds **4** and **4′** is logically treating Ir­(III) chloride precursors **2** and **2′** with a nucleophilic methyl source. In fact, we already explored this approach, in our prior work, using the PMe_2_Ar^Dipp2^-based system and MeMgBr as the nucleophile.^10^ Although we tentatively postulated the formation of an intermediate of type [Cp*Ir­(CH_3_)­(PMe_2_Ar^Dipp2^)]^+^, its high reactivity precluded isolation or even spectroscopic identification at low temperatures. Instead, the only observed product derived from a C–H activation/cyclometalation process involving one of the *iso*-propyl groups of a lateral ring with concomitant release of methane. At variance, the higher stability of the methyl complex **4** in the PMe_2_Ar^Dtbp2^-system, permitted us to isolate this species from the more straightforward halide metathesis route (Scheme 2).

The presence of an interaction between the iridium center and the flanking ring in complex **4** has also been confirmed through the observation of low-frequency ^13^C NMR signals (79.5 and 92.7 ppm for the *ortho-* and *ipso-* carbons, respectively). Compared to precursor **3**, (41.2 and 50.4 ppm for the *ortho-* and *ipso-* carbons, respectively) this interaction appears weaker, consistent with the formation of the Ir-Me bond.

Although for the PMe_2_Ar^Dtbp2^ system we could isolate and characterize the aimed cationic methyl Bergman’s analogue **4**, its intrinsic reactivity became also evident. While compound **4** can be stored in solid state for several weeks without signs of degradation, it readily evolves in solution at room temperature by activating one of the *tert*-butyl groups intramolecularly, yielding the eight-membered iridacycle **5** in quantitative yields after one hour (Scheme 2). This process eliminates methane, observed as a singlet at 0.24 ppm in the ^1^H NMR spectrum. The formation of the Ir-bound CH_2_ moiety is confirmed by the presence of two distinctive doublets at 2.36 and 1.81 ppm (^2^ *J*_HH_ = 8.5 Hz) with intensity corresponding to 1 proton each. In addition, these two resonances exhibit a crosspeak in the HSQC NMR spectrum with the same low-frequency carbon resonance, a doublet at −1.6 ppm (^2^ *J*_CP_ = 14 Hz), consistent with a metal-bound methylene unit.

### Intermolecular Reactivity of Cation [Cp*IrMe­(PMe_2_Ar^Dtbp2^)]**^+^** (**4**)

Despite the intrinsic intramolecular reactivity of the cationic methyl complex **4**, we wondered whether we could still react it with external substrates prior to cyclometalation. In fact, **4** reacts rapidly with H_2_ to form the corresponding hydride species **6** (Scheme 3) as evinced in the ^1^H NMR spectrum by a doublet with 1H intensity that resonates at −16.6 ppm (^2^ *J*_HP_ = 31.1 Hz). As an alternative route, complex **6** can also be formed by treating complex **5** with H_2_. The first synthetic route is supported by DFT studies which shows the oxidative addition pathway of dihydrogen as the rate limiting step (see Figure S35; Δ*G* ^‡^ = 14.0, Δ*G* = −8.3 kcal·mol^–1^), leading to a Ir­(V) dihydride intermediate. Reductive elimination of methane is facile and leads to a σ-methane complex (Δ*G* ^‡^ = 6.3 kcal·mol^–1^), though the alkane is rapidly released upon overcoming a smaller energy barrier of only 1.2 kcal·mol^–1^ toward the thermodynamically favored monohydride species **6** (Δ*G* = −22.0 kcal·mol^–1^). Similarly, computational studies support a σ-bond metathesis mechanism that does not change the oxidation state of the metal (see Scheme S36), which corroborates the yield of **6** from **5**. Thus, coordination of the dihydrogen molecule to **5** results in a σ-H_2_ intermediate (Δ*G* ^‡^ = 8.1, Δ*G* = −6.3 kcal·mol^–1^) which undergoes the elimination of the newly formed methyl group via σ-bond metathesis (Δ*G* ^‡^ = 19.9, Δ*G* = −44.4 kcal·mol^–1^). Finally, the monohydride can also be prepared by the reaction of **2** with PhSiH_3_, once more further corroborating its proposed structure.

**Scheme 3:**
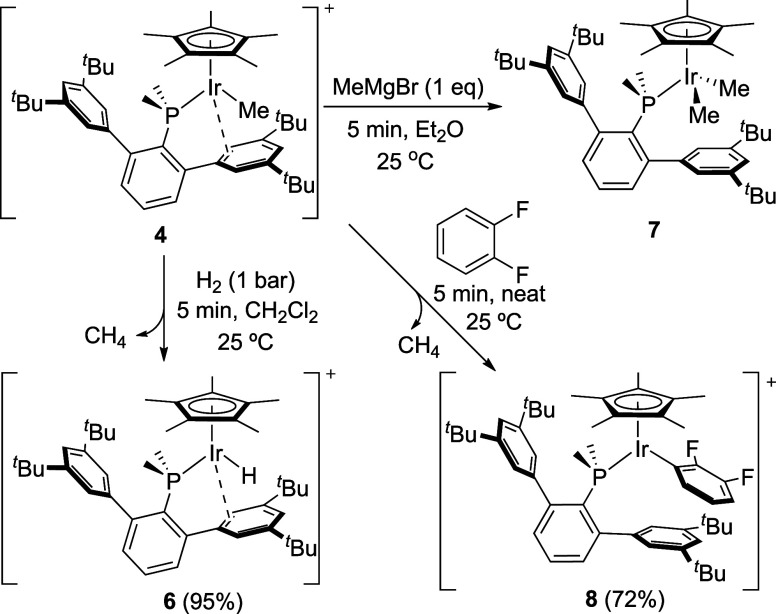
Reactivity of **4** with H_2_, MeMgBr and C_6_H_4_F_2_

Next, we examined the reaction of **4** with an additional equivalent of MeMgBr, which yields the corresponding neutral doubly methylated complex **7** (Scheme 2). In accordance with this, a doublet with an intensity of 6H centered at −0.51 ppm (^3^ *J*_PH_ = 5.4 Hz) is observed by ^1^H NMR. The corresponding carbon resonance appears as a doublet centered at −20.6 ppm (^2^ *J*_CP_ = 8 Hz). Both the proton and the carbon signals appear at more negative frequencies with respect to the monomethylated species **4**, as also occurs with the ^31^P­{^1^H} NMR signal, in accordance with a higher saturation of the iridium center (δ_P_ = −30.0 ppm for **7**, Δδ_P_ = −21.5 ppm with respect to **4**). The mechanism, as supported by DFT studies (see Figure S33), involves the facile (Δ*G* ^‡^ = 10.0 kcal·mol^–1^) direct nucleophilic attack of MeMgBr on the metal center leading to the highly thermodynamically favored product **7** (Δ*G* = −35.9 kcal·mol^–1^). Alternatively, this species can be accessed by directly treating the monochloride precursor **2** with an excess of the Grignard reagent. Efforts to release ethane by reductive elimination to obtain the Ir­(I) complex **3** proved unsuccessful, in agreement with computational calculations (Δ*G* ^‡^ = 54.7 kcal·mol^–1^) (see Figure S34).

Finally, we tested whether **4** would still be capable of intermolecular C–H activation before the cyclometalation toward **5** takes place. To favor the bimolecular process, we used 1,2-difluorobenzene as substrate and solvent, based on previous examples involving the activation of fluorinated aromatic hydrocarbons by related transition metal complexes of group 9.^14^ Upon dissolving **4** in dry 1,2-difluorobenzene, the yellow solution readily darkens to an intense red color due to the formation of the C–H activation product **8** (Scheme 3). At variance, when the cyclometalated compound **5** was dissolved in 1,2-difluorobenzene, no reactivity could be detected.

Alternatively, when less activated substrates as benzene or toluene were used, the intramolecular activation toward **5** outcompeted the intermolecular reaction preventing us from isolating any pure compound for full characterization. Besides, it is important to comment that the bulkier environment of this complex results in significantly slower substrate C–H activation compared to the parent Bergman’s related Ir system, where benzene activation was completed in around 10 h (c.f. ∼10% conversion for **4** after two days and within a mixture of species, including **5**).

The formation of **8** was monitored by ^31^P­{^1^H} NMR, revealing the appearance of a new resonance at 1.8 ppm, along with two new fluorine signals at −119.6 and −138.9 ppm in the ^19^F­{^1^H} NMR spectrum. The existence of the two latter resonances aligns with the functionalization of 1,2-difluorobenzene (c.f. δ_F_ = −139.1 ppm), also supported by the disappearance of resonances associated to the Ir–Me unit. Monodimensional ^1^H, ^19^F HOESY NMR experiments were performed to assign these fluorine signals. Since no dipolar coupling with aromatic protons was found for the signal at −119.6 ppm, we assigned it to the fluorine in *ortho-*position to the activated carbon. Consistently, the fluorine signal at −138.9 ppm correlates with the para proton of the arene, in accordance with its *meta*-position with respect to the Ir–C moiety (see Figure S27). Both fluorine signals appear as doublets due to coupling to each other (^3^ *J*_FF_ = 30 Hz).

Crystals suitable for X-ray diffraction were obtained from a saturated benzene/1,2-difluorobenzene solution. The structure confirms the activation of 1,2-difluorobenzene in the *ortho*-position to the fluorine atom, with an iridium-carbon distance of 2.059(4) Å (Figure 5). The structure exhibits only a slight pyramidalization of the *ipso*-carbon of one of the lateral rings, defined by a torsion angle of 166° (C39–C40–C41–C42). For these specific complexes, this pyramidalization seems to correlate relatively well with the interaction energy of the phosphine with the metal (Figure S38), as it enables the stabilization of the unsaturated metal fragment by weak π-type interactions. In this case, the long Ir–C40 and Ir–C41 distances of 3.239(4) and 3.575(4) Å, respectively, permit minimal additional stabilization, which likely contributes to the reduced thermal stability of **8**, whose spectroscopic characterization had to be run at low temperature to avoid decomposition.

**Figure 5:**
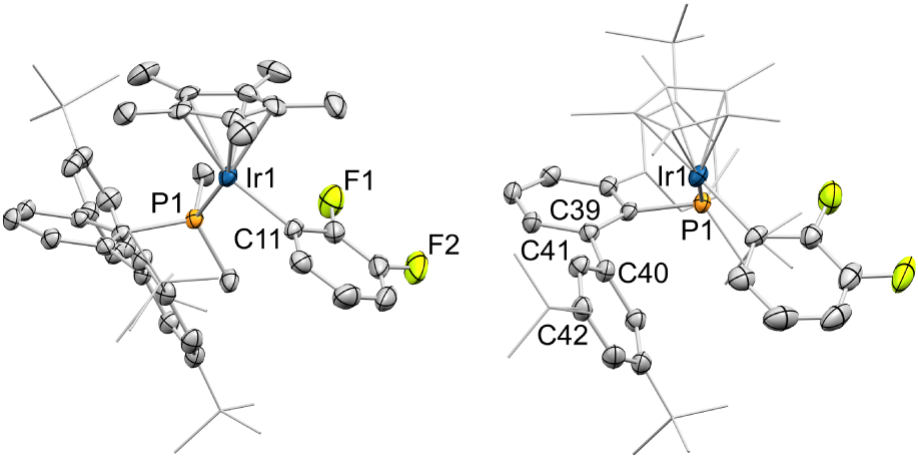
Two views of the ORTEP diagram of complex **8**. Triflate counterion and all hydrogen atoms are excluded for clarity. Thermal ellipsoids are set at 50% probability.

Interestingly, despite the reduced reactivity of **4** compared to the Bergman analogue, the activation of 1,2-difluorobenzene exclusively leads to the 2,3-isomer, while no hint of the 3,4-isomer was detected. This contrasts with previously reported Rh-based complexes,^14^ that often have led to a mixture of the two isomers that upon heating evolve to the more stable 2,3-isomer. Nonetheless, the aforediscussed and more related [Cp*IrMe­(PMe_3_)­(CH_2_Cl_2_)]^+^ species yielded the two possible isomers, with the preferred one being instead the 3,4-substituted one, clearly differing from our reported system. This was somewhat surprising, as our bulkier phosphine leads to the activation of the slightly more congested position.

## Conclusion

In summary, we have successfully isolated bulkier versions of two paradigmatic iridium compounds, [Cp*Ir­(PMe_3_)] and [Cp*Ir­(Me)­PMe_3_]^+^, that triggered the rapid evolution of C–H bond activation chemistry, but whose derivatization had remained mostly unexplored. In particular, we describe the isolation of the Ir­(I) neutral compounds [Cp*Ir­(PMe_2_Ar^Dtbp2^)] (**3**) and [Cp*Ir­(PMe_2_Ar^Dipp2^)] (**3′**), whose stability contrast to the transient nature of the photogenerated [Cp*Ir­(PMe_3_)]. The enhanced stability derives from the kinetic and thermodynamic stabilization provided by the bulky terphenyl phosphines used, where strong interactions between the metal and one of the lateral aryl rings have been experimentally and computationally analyzed.

Interestingly, despite their apparent similar nature, these two Ir­(I) species exhibit quite distinct reactivity toward methyl trifluoromethanesulfonate, as only **3** reacts to yield the corresponding cationic methyl complex [Cp*IrMe­(PMe_2_Ar^Dtbp2^)]­[OTf] (**4**), while **3′** was unreactive. Complex **4** readily undergoes an intramolecular C–H bond activation process of one of the *tert*-butyl substituents of the phosphine, to produce an eight-membered iridacycle (**5**). Nonetheless, the reactivity of **4** prior to cyclometalation can still be investigated, as we have done for dihydrogen, phenylsilane and even to selectively activate one of the C–H bonds of 1,2-difluorobenzene. This latter result underscores the potential of this type of compounds for intermolecular C–H activation reactions even under congested environments.

## Experimental Section

### General Considerations

All manipulations were carried out using standard Schlenk techniques, under high purity nitrogen. All solvents were dried and distilled under nitrogen prior to use. *n*-Pentane (C_5_H_12_) was distilled over sodium. Diethyl ether was distilled over sodium/benzophenone. CH_2_Cl_2_ and CD_2_Cl_2_ were dried and distilled over CaH_2_. Complex **2′** ^9^ and PMe_2_Ar^Dtbp2^ ^11^ were prepared according to literature methods. Solution NMR spectra were recorded on Bruker DRX-400 and DRX-500 spectrometers. Spectra were referenced to external SiMe_4_ (δ: 0 ppm) using the residual proton solvent peaks as internal standards (^1^H NMR experiments), or the characteristic resonances of the solvent nuclei (^13^C NMR experiments), while ^31^P was referenced to H_3_PO_4_. Spectral assignments were made by routine one- and two-dimensional NMR experiments (^1^H, ^1^H­{^31^P}, ^13^C­{^1^H}, ^31P^{^1^H}, COSY, HOESY, HSQC and HMBC) where appropriate. In all NMR characterizations, the labels dipp′ and dtbp′ represent the aryl ring that interacts with the Ir center, or is closest to it, to distinguish it from the other unit. In the absence of a specification, the signals are considered equivalent due to symmetry. Elemental analyses were performed at Instituto de Investigaciones Químicas, Sevilla, with a LECO TruSpec CHN elemental analyzer. The values are reported as a percentage by weight. No uncommon hazards are noted.

**Complex 1.** To a dry CH_2_Cl_2_ solution (12 mL) of [IrCp*Cl_2_]_2_ (74 mg, 0.093 mmol), a dry CH_2_Cl_2_ solution of PMe_2_Ar^Dtbp2^ (100 mg, 0.195 mmol) was added at room temperature. There was an instantaneous color change from orange to bright orange-red. The solution was stirred for 30 min. Then, the solvent was evaporated under reduced pressure. The residue was washed with pentane (3 mL). Yield: (158 mg, 92%). **Anal. Calcd.** for C_46_H_66_Cl_2_IrP: C, 60.51; H, 7.29. **Found**: C, 60.59; H, 7.28. ^**1**^ **H NMR** (500 MHz, CD_2_Cl_2_, 25 °C) δ: 7.48 (s, 2H, *p*-dtbp), 7.41 (s, 2H, *o*-dtbp), 7.35 (td, ^3^ *J*_HH_ = 7.5 Hz, ^5^ *J*_HP_ = 1.5 Hz, 1H, *p*-C_6_H_3_), 7.25 (dd, ^3^ *J*_HH_ = 7.5 Hz, ^4^ *J*_HP_ = 2.1 Hz, 2H, *m*-C_6_H_3_), 7.03 (s, 2H, *o*-dtbp′), 1.63 (d, ^2^ *J*_HP_ = 10.7 Hz, 6H, PMe_2_), 1.45 (s, 18H, (CH_3_)_3_C), 1.37 (s, 18H, (CH_3_)_3_C), 1.34 (d, ^4^ *J*_HP_ = 1.7 Hz, 15H, C_5_Me_5_) ppm. ^**13**^ **C­{** ^**1**^ **H} NMR** (125 MHz, CD_2_Cl_2_, 25 °C) δ: 151.4 (d, ^2^ *J*_CP_ = 8 Hz, *o*-C_6_H_3_), 151.3 (s, *m*-dtbp), 149.0 (s, *m*-dtbp), 142.5 (d, ^3^ *J*_CP_ = 3 Hz, *ipso*-dtbp), 132.1 (d, ^3^ *J*_CP_ = 8 Hz, *m*-C_6_H_3_), 128.5 (d, ^4^ *J*_CP_ = 3 Hz, *p*-C_6_H_3_), 128.4 (d, ^1^ *J*_CP_ = 41 Hz, *ipso*-C_6_H_3_), 127.8 (s, *o*-dtbp), 124.0 (s, *o*-dtbp), 122.5 (s, *p*-dtbp), 91.20 (d, ^2^ *J*_CP_ = 3 Hz, *C*_5_Me_5_), 35.3 (s, (CH_3_)_3_ *C*), 35.0 (s, (CH_3_)_3_ *C*), 31.8 (s, (*CH*_3_)_3_C), 31.5 (s, (*CH*_3_)_3_C), 18.9 (d, ^1^ *J*_CP_ = 36 Hz, PMe_2_), 8.9 (s, C_5_ *Me*_5_) ppm. ^**31**^ **P­{** ^**1**^ **H} NMR** (202 MHz, CD_2_Cl_2_, 25 °C) δ: −19.1 ppm.

**Complex 2.** A dry CH_2_Cl_2_ solution (12 mL) of **1** (120 mg, 0.133 mmol) and NaBAr^F^ (118 mg, 0.133 mmol) was stirred at room temperature for 30 min. The solution boasts a dark brown-red color. The solvent was evaporated under reduced pressure and the residue was washed with pentane (3 × 5 mL). Yield: 215 mg (93%). **Anal. Calcd.** for C_78_H_79_BF_24_IrP: C, 54.90; H, 4.67. **Found**: C, 54.91; H, 4.63. ^**1**^ **H NMR** (500 MHz, CD_2_Cl_2_, 25 °C) δ: 7.85 (s, 2H, *p*-dtbp), 7.76 (m, 8H, *o*-Ar), 7.59 (s, 4H, *p*-Ar), 7.35 (td, 1H, ^3^ *J*_HH_ = 7.6 Hz, ^5^ *J*_HP_ = 2.0 Hz, *p*-C_6_H_3_), 7.21 (s, 4H, *o*-dtbp), 6.81 (dd, 2H, ^3^ *J*_HH_ = 7.6 Hz, ^4^ *J*_HP_ = 2.7 Hz, *m*-C_6_H_3_), 1.74 (d, 6H, ^2^ *J*_HP_ = 11.0 Hz, PMe_2_), 1.40 (s, 36H, (CH_3_)_3_C), 1.21 (d, 15H, ^4^ *J*_HP_ = 1.6 Hz, C_5_Me_5_) ppm. ^**13**^ **C­{** ^**1**^ **H} NMR** (125 MHz, CD_2_Cl_2_, 25 °C) δ: 162.2 (q, ^1^ *J*_CB_ = 49 Hz, *ipso*-Ar), 151.7 (br, *m*-dtbp), 147.5 (d, ^2^ *J*_CP_ = 13 Hz, *o*-C_6_,H_3_), 135.3 (s, *o*-Ar), 132.0 (br, *p*-C_6_H_3_), 131.7 (d, ^3^ *J*_CP_ = 9 Hz, *m*-C_6_H_3_), 129.4 (q, ^2^ *J*_CF_ = 32 Hz, *m*-Ar), 127.1 (br, *ipso*-C_6_H_3_), 126.5–125.2 (br, overlapped *ipso*-dtbp), 125.6 (br, overlapped *o*-dtbp, *p*-dtbp), 125.1 (d, ^1^ *J*_CF_ = 272 Hz, CF_3_), 117.9 (m, *p*-Ar), 95.7 (*C*_5_Me_5_), 35.7 (s, (CH_3_)_3_ *C*), 31.4 (s, (*C*H_3_)_3_C), 16.0 (d, ^1^ *J*_CP_ = 40 Hz, PMe_2_), 8.6 (s, C_5_ *Me*_5_) ppm. ^**31**^ **P­{** ^**1**^ **H} NMR** (202 MHz, CD_2_Cl_2_, 25 °C) δ: 8.9 ppm.

**Complex 3.** A dry CH_2_Cl_2_ solution (12 mL) of **2** (120 mg, 0.133 mmol) and CoCp_2_ (51 mg, 0.270 mmol) was stirred at room temperature for 30 min, observing the formation of orange-yellow cobaltocenium salts. The solvent was evaporated and the residue was extracted with pentane (3 × 5 mL). A final evaporation under reduced pressure gave a dark yellow solid. Yield: 92 mg (83%). **Anal. Calcd.** for C_46_H_66_IrP: C, 65.60; H, 7.90. **Found**: C, 65.74; H, 7.83. ^**1**^ **H NMR** (500 MHz, CD_2_Cl_2_, 25 °C) δ: 7.43 (s, 1H, *o*-dtbp), 7.04 (m, 4H, overlapped *p*-C_6_H_3_, *p*-dtbp, *o*-dtbp, *m*-C_6_H_3_), 6.67 (d, 1H, ^4^ *J*_HP_ = 7.1 Hz, *m*-C_6_H_3_), 6.51 (s, 1H, *o*-dtbp′), 6.26 (s, 1H, *p*-dtbp′), 3.08 (d, 1H, ^2^ *J*_HP_ = 8.3 Hz, *o*-dtbp′), 1.68 (d, 3H, ^2^ *J*_HP_ = 10.0 Hz, PMe), 1.59 (br, 15H, C_5_Me_5_), 1.33 (s, 18H, (CH_3_)_3_C), 1.32 (s, 9H, (CH_3_)_3_C), 1.25 (d, 3H, ^2^ *J*_HP_ = 10.0 Hz, PMe), 1.23 (s, 9H, (CH_3_)_3_C) ppm. ^**13**^ **C­{** ^**1**^ **H} NMR** (125 MHz, CD_2_Cl_2_, 25 °C) δ: 165.7 (d, ^2^ *J*_CP_ = 29 Hz, *o*-C_6_H_3_), 152.9 (s, *m*-dtbp′), 150.5 (br, *m*-dtbp), 149.7 (br, *m*-dtbp), 147.7 (br, *o*-C_6_H_3_), 141.4 (br, *ipso*-dtbp), 138.1 (s, *m*-dtbp′), 133.2 (d, ^1^ *J*_CP_ = 46 Hz, *ipso*-C_6_H_3_), 128.7 (br, *p*-C_6_H_3_), 127.7 (d, ^3^ *J*_CP_ = 5 Hz, *m*-C_6_H_3_), 126.7 (d, ^3^ *J*_CP_ = 13 Hz, *m*-C_6_H_3_), 125.1 (br, *o*-dtbp), 124.7 (br, *p*-dtbp), 123.3 (s, *o*-dtbp′), 121. (s, *o*-dtbp), 109.8 (s, *p*-dtbp′), 90.4 (d, ^2^ *J*_CP_ = 3 Hz, *C*_5_Me_5_), 50.4 (br, *ipso*-dtbp′), 41.2 (s, *o*-dtbp′), 36.1 (s, (CH_3_)_3_ *C*), 35.2 (br, (CH_3_)_3_ *C*), 34.7 (s, (CH_3_)_3_ *C*), 31.6 (s, (*C*H_3_)_3_C), 31.2 (s, (*C*H_3_)_3_C), 30.9 (s, (*C*H_3_)_3_C), 20.0 (d, ^1^ *J*_CP_ = 34 Hz, PMe), 15.5 (d, ^1^ *J*_CP_ = 38 Hz, PMe), 9.7 (C_5_ *Me*_5_) ppm. ^**31**^ **P­{** ^**1**^ **H} NMR** (162 MHz, CD_2_Cl_2_, 25 °C) δ: −1.6 ppm.

**Complex 4.** A solution of **3** (100 mg, 0.119 mmol) in 10 mL of dry pentane was prepared and 1 equivalent of MeOTf (13 μL, 0.119 mmol) was added while stirring. Immediately after the addition, a solid began to precipitate. The reaction mixture was then stirred for one hour to ensure that the precursor had been fully converted. The solution was then filtered, and the solid was washed with dry pentane and cold, dry diethyl ether. The solid was finally dried under vacuum to yield a fine yellow solid. Yield: 102 mg (85%). **4** is stable in the solid-state but rapidly evolves into complex **5** in solution at RT. For this reason, **4** was characterized at low temperature. **Anal. Calcd.** For C_48_H_69_F_3_IrO_3_PS: C, 57.29; H, 6.91; S, 3.19. **Found**: C, 57.22; H, 6.45; S, 3.50. ^**1**^ **H NMR** (400 MHz, CD_2_Cl_2_, 10 °C) δ: 7.54 (s, 2H, *p*-dtbp), 7.33 (td, 1H, ^3^ *J*_HH_ = 7.4 Hz, ^5^ *J*_HP_ = 1.7 Hz, *p*-C_6_H_3_), 7.05 (s, 2H, *o*-dtbp), 6.78 (br, 2H, *m*-C_6_H_3_), 5.95 (br, 2H, *o*-dtbp′), 1.46 (br, 6H, PMe_2_), 1.37 (s, 18H, (CH_3_)_3_C), 1.31 (s, 18H, (CH_3_)_3_C), 1.29 (br, 15H, (C_5_Me_5_)), 0.56 (d, 3H, ^2^ *J*_HP_ = 7.2 Hz, Ir-Me) ppm. ^**1**^ **H NMR** (400 MHz, CD_2_Cl_2_, −80 °C) δ: 7.40 (s, 1H, *p*-dtbp), 7.34 (s, 1H, *p*-dtbp′), 7.26 (br, 1H, *p*-C_6_H_3_), 7.15 (s, 1H, *o*-dtbp), 7.12 (br, 1H, *m*-C_6_H_3_), 6.98 (s, 1H, *o*-dtbp), 6.71 (s, 1H, *o*-dtbp′), 6.39 (m, 1H, *m*-C_6_H_3_), 4.58 (s, 1H, *o*-dtbp′), 1.89 (d, 3H, ^2^ *J*_HP_ = 9.8 Hz, PMe), 1.28 (s, 9H, (CH_3_)_3_C), 1.24 (s, 18H, (CH_3_)_3_C), 1.18 (br, 24H, overlapped (CH_3_)_3_C, C_5_Me_5_), 0.72 (d, 3H, ^2^ *J*_HP_ = 10.3 Hz, PMe), 0.44 (br, 3H, Ir-Me) ppm. ^**13**^ **C­{** ^**1**^ **H} NMR** (100 MHz, CD_2_Cl_2_, −80 °C) δ: 151.6 (s, *m*-dtbp), 149.9 (s, *m*-dtbp), 145.4 (overlapped *ipso*-dtbp), 145.1 (br, overlapped *m*-dtbp′), 137.7 (br, *o*-C_6_H_3_), 133.3 (d, ^1^ *J*_CP_ = 63 Hz, *ipso*-C_6_H_3_), 130.9 (br, *p*-C_6_H_3_), 130.2 (br, *o*-C_6_H_3_), 130.0 (br, *m*-C_6_H_3_), 129.8 (br, *m*-C_6_H_3_), 125.9 (s, *o*-dtbp′), 123.6 (s, *o*-dtbp), 122.4 (s, *o*-dtbp), 121.4 (s, *p*-dtbp), 119.9 (s, *p*-dtbp′), 118.2 (s, OTf), 96.8 (s, *C*_5_Me_5_), 92.7 (br, *ipso*-dtbp′), 79.5 (s, *o*-dtbp′), 35.9 (s, (CH_3_)_3_ *C*), 34.4 (s, (CH_3_)_3_ *C*), 34.3 (s, (CH_3_)_3_ *C*), 34.2 (s, (CH_3_)_3_ *C*), 30.5 (s, (*C*H_3_)_3_C), 30.4 (s, (*C*H_3_)_3_C), 29.9 (s, (*C*H_3_)_3_C), 29.7 (s, (*C*H_3_)_3_C), 18.2 (d, ^1^ *J*_CP_ = 39 Hz, PMe), 8.4 (d, ^1^ *J*_CP_ = 40 Hz, PMe), 7.4 (s, C_5_Me_5_), −15.5 (d, ^2^ *J*_CP_ = 14 Hz, Ir-Me) ppm. ^**31**^ **P­{** ^**1**^ **H} NMR** (162 MHz, CD_2_Cl_2_, −80 °C) δ: 8.5 ppm.

**Complex 5.** A dry CH_2_Cl_2_ solution (5 mL) of **4** (100 mg, 0.100 mmol) was left stirring at room temperature for 1 h. The solvent was evaporated, and the residue was washed with pentane (3 × 5 mL) and dried under reduced pressure to obtain an orange solid. Yield: 89 mg (90%). **Anal. Calcd.** for C_47_H_65_F_3_IrO_3_PS: C, 57.01; H, 6.62; S, 3.24. **Found**: C, 57.03; H, 6.54; S, 3.58. ^**1**^ **H NMR** (500 MHz, CD_2_Cl_2_, 25 °C) δ: 7.62 (s, 1H, *p*-dtbp), 7.50 (td, 1H, ^3^ *J*_HH_ = 7.5 Hz, ^5^ *J*_HP_ = 2.5 Hz, *p*-C_6_H_3_), 7.38 (m, 3H, overlapped, *o*-dtbp, *m*-C_6_H_3_), 7.11 (s, 1H, *p*-dtbp′), 6.85 (s, 1H, *o*-dtbp′), 6.83 (dd, 1H, ^3^ *J*_HH_ = 7.5 Hz, ^4^ *J*_HP_ = 2.7 Hz, *m*-C_6_H_3_), 3.97 (s, 1H, *o*-dtbp′), 2.36 (d, 1H, ^2^ *J*_HH_ = 8.5 Hz, CH_2_), 1.81 (dd, 1H, ^2^ *J*_HH_ = 8.5 Hz, ^3^ *J*_HP_ = 2.7 Hz, CH_2_), 1.50 (d, 15H, C_5_Me_5_), 1.43 (s, 9H, (CH_3_)_3_C), 1.41 (s, 18H, (CH_3_)_3_C), 1.27 (s, 12H, overlapped (CH_3_)_3_C, PMe_2_) ppm. ^**13**^ **C­{** ^**1**^ **H} NMR** (100 MHz, CD_2_Cl_2_, 25 °C) δ: 158.2 (d, ^2^ *J*_CP_ = 25 Hz, *o*-C_6_H_3_), 153.1 (br, *m*-dtbp), 152.5 (s, *m*-dtbp′), 148.1 (d, ^2^ *J*_CP_ = 5 Hz, *o*-C_6_H_3_), 147.4 (s, *m*-dtbp′), 139.1 (d, ^3^ *J*_CP_ = 3 Hz, *ipso*-dtbp), 133.7 (d, ^4^ *J*_CP_ = 3 Hz, *p*-C_6_H_3_), 131.1 (d, ^3^ *J*_CP_ = 17 Hz, *m*-C_6_H_3_), 129.9 (d, ^3^ *J*_CP_ = 9 Hz, *m*-C_6_H_3_), 124 (s, *o*-dtbp′), 123.8 (br, overlapped *o*-dtbp), 123.4 (s, *p*-dtbp), 122.2 (d, ^1^ *J*_CP_ = 70 Hz, *ipso*-C_6_H_3_), 118.2 (s, *p*-dtbp′), 98.2 (s, *C*_5_Me_5_), 87.2 (d, ^3^ *J*_CP_ = 3 Hz, *ipso*-dtbp′) 71.1 (s, *o*-dtbp′), 36.6 (s, (CH_3_)_3_ *C*), 35.5 (s, (CH_3_)_3_ *C*), 31.6 (s, (*C*H_3_)_3_C), 31.4 (s, (*C*H_3_)_3_C), 30.6 (s, (*C*H_3_)_3_C), 8.9 (d, ^1^ *J*_CP_ = 38 Hz, PMe), 8.6 (s, C_5_ *Me*_5_), 1.8 (d, ^1^ *J*_CP_ = 40 Hz, PMe), −1.6 (d, ^2^ *J*_CP_ = 14 Hz, CH_2_) ppm. ^**31**^ **P­{** ^**1**^ **H} NMR** (162 MHz, CD_2_Cl_2_, 25 °C) δ: −6.9 ppm.

**Complex 6.** A dry CH_2_Cl_2_ solution (12 mL) of **2** (100 mg, 0.057 mmol) and PhSiH_3_ (10.4 μL, 0.114 mmol) was stirred at room temperature for 30 min. The solvent was evaporated. The residue was dissolved in Et_2_O (4 mL) and precipitated with pentane (15 mL). The solvent was filtered off and the precipitated was dried under reduce pressure to afford a yellow solid. Yield: 82 mg (84%). **Anal. Calcd**. for C_78_H_79_BF_24_IrP: C, 54.90; H, 4.67. **Found**: C, 54.90; H, 4.67. ^**1**^ **H NMR** (500 MHz, CD_2_Cl_2_, 25 °C) δ: 7.72 (m, 8H, *o*-Ar), 7.56 (s, 4H, *p*-Ar), 7.54 (m, 1H, overlapped *p*-dtbp), 7.38 (td, 1H, ^3^ *J*_HH_ = 7.8 Hz, ^5^ *J*_HP_ = 2.3 Hz, *p*-C_6_H_3_), 7.16 (br, 1H, *p*-dtbp′), 7.14 (t, 1H, ^4^ *J*_HH_ = 1.8 Hz, *o*-dtbp), 7.10 (ddd, 1H, ^3^ *J*_HH_ = 7.4 Hz, ^4^ *J*_HP_ = 3.3 Hz, ^4^ *J*_HH_ = 1.0 Hz, *m*-C_6_H_3_), 7.01 (t, 1H, ^4^ *J*_HH_ = 1.8 Hz, *o*-dtbp), 6.93 (ddd, 1H, ^3^ *J*_HH_ = 8.0 Hz, ^4^ *J*_HP_ = 2.7 Hz, ^4^ *J*_HH_ = 1.0 Hz, *m*-C_6_H_3_), 6.90 (br, 1H, *o*-dtbp′), 5.06 (br, 1H, *o*-dtbp′), 1.88 (d, 3H, ^2^ *J*_HP_ = 11.0 Hz, PMe), 1.60 (d, 3H, ^2^ *J*_HP_ = 11.4 Hz, PMe), 1.55 (d, 15H, ^4^ *J*_HP_ = 1.3 Hz, C_5_Me_5_), 1.44 (s, 9H, (CH_3_)_3_C), 1.38 (s, 9H, (CH_3_)_3_C), 1.33 (s, 9H, (CH_3_)_3_C), 1.32 (s, 9H, (CH_3_)_3_C), −16.6 (d, 1H, ^2^ *J*_HP_ = 31.1 Hz, Ir-H) ppm. ^**13**^ **C­{** ^**1**^ **H} NMR** (125 MHz, CD_2_Cl_2_, 25 °C) δ: 162.2 (q, ^1^ *J*_CB_ = 50 Hz, *ipso*-Ar), 154.1 (d, ^2^ *J*_CP_ = 26 Hz, *o*-C_6_H_3_), 151.3 (s, *m*-dtbp), 150.7 (s, *m*-dtbp), 150.3 (s, *m*-dtbp), 148.6 (br, *o*-C_6_H_3_), 147.3 (s, *m*-dtbp), 138.7 (br, *ipso*-dtbp), 135.2 (s, *o*-Ar), 132.3 (d, ^3^ *J*_CP_ = 6 Hz, *m*-C_6_H_3_), 131.9 (br, *p*-C_6_H_3_), 131.3 (d, ^3^ *J*_CP_ = 14 Hz, *m*-C_6_H_3_), 129.3 (overlapped *ipso-*C_6_H_3_), 129.0 (q, ^2^ *J*_CF_ = 32 Hz, *m*-Ar), 125.0 (d, ^1^ *J*_CF_ = 272 Hz, CF_3_), 124.8 (s, *o*-dtbp), 124.4 (s, *o*-dtbp′), 122.9 (s, *p*-dtbp), 119.9 (s, *p*-dtbp′), 117.9 (br, *p*-Ar), 99.3 (s, *C*_5_Me_5_), 86.5 (d, ^2^ *J*_CP_ = 4 Hz, *ipso*-dtbp′), 62.7 (s, *o*-dtbp′), 36.6 ((CH_3_)_3_ *C*), 35.7 ((CH_3_)_3_ *C*), 35.2 ((CH_3_)_3_ *C*), 31.5 ((*C*H_3_)_3_C), 30.7 ((*C*H_3_)_3_C), 30.6 ((*C*H_3_)_3_C), 26.7 (d, ^1^ *J*_CP_ = 46 Hz, PMe), 15.7 (d, ^1^ *J*_CP_ = 39 Hz, PMe), 9.4 (s, C_5_Me_5_) ppm. ^**31**^ **P­{** ^**1**^ **H} NMR** (202 MHz, CD_2_Cl_2_, 25 °C) δ: −4.4 ppm.

**Complex 7.** A sample of **7** was generated in situ within a J-Young NMR tube in CD_2_Cl_2_ starting from **1** (20 mg, 0.012 mol) and 2 equivalents of MeMgBr (4 μL, 3.0 M solution in diethyl ether). The red solution of the precursor turns bright yellow immediately upon addition of the Grignard reagent, resulting in the quantitative spectroscopic formation of **7**. The high instability of this complex prevented us from its isolation in analytically pure form and therefore it was characterized in situ. ^**1**^ **H NMR** (500 MHz, CD_2_Cl_2_, 25 °C) δ: 7.41 (s, 2H, overlapped *p*-dtbp), 7.37 (s, 2H, *o*-dtbp), 7.14 (td, ^3^ *J*_HH_ = 7.5 Hz, ^5^ *J*_HP_ = 1.5 Hz, 1H, *p*-C_6_H_3_), 7.06 (m, 4H, overlapped *m*-C_6_H_3_, *o*-dtbp), 1.41 (br, 18H, (CH_3_)_3_C), 1.35 (br, 15H, C_5_Me_5_), 1.34 (br, 18H, (CH_3_)_3_C), 1.26 (d, 6H, overlapped PMe_2_), −0.51 (d, 6H, ^3^ *J*_HP_ = 5.4 Hz, IrMe_2_) ppm. ^**13**^ **C­{** ^**1**^ **H} NMR** (125 MHz, CD_2_Cl_2_, 25 °C) δ: 150.7 (s, *m*-dtbp), 148.8 (d, ^2^ *J*_CP_ = 9 Hz, *o*-C_6_H_3_), 148.7 (s, *m*-dtbp), 143.8 (d, ^3^ *J*_CP_ = 3 Hz, *ipso*-dtbp), 132.2 (d, ^1^ *J*_CP_ = 31 Hz, *ipso*-C_6_H_3_), 132.0 (d, ^1^ *J*_CH_ = 7 Hz, *m*-C_6_H_3_), 127.4 (s, *o*-dtbp), 126.4 (br, *p*-C_6_H_3_), 124.1 (s, *o*-dtbp), 121.8 (overlapped *p*-dtbp), 121.7 (overlapped *ipso*-dtbp), 91.2 (d, ^2^ *J*_CP_ = 4 Hz, *C*_5_Me_5_), 35.3 (s, (CH_3_)_3_ *C*), 35.0 (s, (CH_3_)_3_ *C*), 31.7 (s, (*C*H_3_)_3_C), 31.5 (s, (*C*H_3_)_3_C), 20.7 (d, ^1^ *J*_CP_ = 35 Hz, PMe_2_), 8.5 (s, C_5_Me_5_), −20.6 (d, ^2^ *J*_CP_ = 8 Hz, IrMe_2_) ppm. ^**31**^ **P­{** ^**1**^ **H} NMR** (202 MHz, CD_2_Cl_2_, 25 °C) δ: −30.0 ppm.

**Complex 8.** Complex **4** (100 mg, 0.100 mmol) was left to stir into a neat solution of dry 1,2-difluorobenzene (5 mL). Over time, a darkening of the solution is observed, and after one hour, a homogeneous red mixture is obtained. The solvent was then removed in vacuum and the solid was washed with pentane (2 × 3 mL). A final evaporation under reduced pressure gave a red solid. Yield: 80 mg (72%).

**8** is stable in the solid state, but it degrades in solution at room temperature. For this reason, it was characterized at low temperature. **Anal. Calcd.** for C_53_H_69_F_5_IrO_3_PS: C, 57.64; H, 6.30; S, 2.90. **Found**: C, 57.9; H, 6.26; S, 2.59. ^**1**^ **H NMR** (400 MHz, CD_2_Cl_2_, −80 °C) δ: 7.66 (s, 1H, *p*-dtbp′), 7.51 (br, 1H, *m*-C_6_H_3_), 7.47 (s, 1H, *p*-dtbp), 7.39 (br, 1H, *o*-dtbp), 7.24 (br, overlapped, 2H, *o*-dtbp, *o*-dtbp′), 7.17 (br, 1H, *m*-C_6_H_3_), 7.11 (br, 1H, *p*-C_6_H_3_), 6.87 (br, 1H, *o*-dtbp′), 6.69 (br, 1H, *m*-odfb), 6.60 (m, 1H, *p*-odfb), 4.89 (br, 1H, *o*-odfb), 1.89 (d, 3H, ^2^ *J*_HP_ = 7.5 Hz, PMe), 1.45 (br, 9H, CH_3_)_3_C), 1.35 (br, 9H, (CH_3_)_3_C), 1.29 (br, 9H, CH_3_)_3_C), 1.15 (br, 15H, C_5_Me_5_), 1.06 (br, PMe), 0.93 (br, 9H, CH_3_)_3_C) ppm. ^**13**^ **C­{** ^**1**^ **H} NMR** (100 MHz, CD_2_Cl_2_, −80 °C) δ: 151.8 (s, *m*-dtbp′), 151.5 (s, *m*-dtbp), 150.7 (s, *m*-dtbp′), 150.6 (d, ^1^ *J*_CP_ = 15 Hz, *ipso*-C_6_H_3_), 149.7 (s, *m*-dtbp), 149.5 (br, *ipso*-dtbp′), 148.2 (br, *o*-C_6_H_3_), 148.0 (d, ^2^ *J*_CP_ = 5 Hz, *ipso*-dtbp) 147.4 (br, *o*-C_6_H_3_), 146.7 (m, *ipso*-odfb) 146.2 (m, *m*-odfb), 145.8 (m, *o*-odfb), 130.3 (m, overlapped *o*-dtbp′, *m*-C_6_H_3_), 126.5 (s, *p*-dtbp′), 126.0 (m, overlapped *o*-odfb, *m*-odfb), 124.9 (s, *o*-dtbp), 124.4 (br, *p*-C_6_H_3_), 123.3 (s, *o*-dtbp), 122.6 (s, *o*-dtbp′), 121.7 (s, *p*-dtbp), 116.9 (d, ^3^ *J*_CP_ = 6 Hz, *m*-C_6_H_3_), 116.7 (d, ^3^ *J*_CP_ = 6 Hz, *m*-C_6_H_3_), 112.2 (d, ^2^ *J*_CF_ = 17 Hz, *p*-odfb), 96.3 (br, *C*_5_Me_5_), 34.8 (s, (CH_3_)_3_ *C*), 34.5 (s, (CH_3_)_3_ *C*), 34.4 (s, (CH_3_)_3_ *C*), 34.3 (s, (CH_3_)_3_ *C*), 30.7 (s, (CH_3_)_3_C), 31.5 (s, overlapped (CH_3_)_3_C), 30.2 (s, (CH_3_)_3_C), 22.3 (m, overlapped PMe_2_), 9.6 (s, C_5_Me_5_) ppm. ^**31**^ **P­{** ^**1**^ **H} NMR** (125 MHz, CD_2_Cl_2_, 25 °C) δ: 1.8 ppm. ^**31**^ **P­{** ^**1**^ **H} NMR** (125 MHz, CD_2_Cl_2_, −80 °C) δ: 0.3 ppm. ^**19**^ **F­{** ^**1**^ **H} NMR** (376 MHz, CD_2_Cl_2_, −80 °C) δ: −79.1 (s, OTf), −119.6 (d, ^3^ *J*_FF_ = 30 Hz, *o*-odfb), −138.9 (d, ^3^ *J*_FF_ = 30 Hz, *m*-odfb) ppm.

**Complex 3′.** A dry CH_2_Cl_2_ solution (12 mL) of [IrCl­(PAr^Dipp2^)­Cp*]­BAr^F^ (100 mg, 0.059 mmol) and CoCp_2_ (23 mg, 0.121 mmol) was stirred at room temperature for 30 min, observing the formation of orange-yellow cobaltocenium salts. The solvent was evaporated and the residue was extracted with pentane (3 × 5 mL). A final evaporation under reduced pressure gave a dark yellow solid. Yield: 34 mg (72%). **Anal. Calcd**. for C_43_H_61_IrP: C, 64.47; H, 7.67. **Found**: C, 64.50; H, 7.69. ^**1**^ **H NMR** (500 MHz, CD_2_Cl_2_, −20 °C) δ: 7.34 (t, 1H, ^3^ *J*_HH_ = 7.8 Hz, *p*-dipp), 7.22 (d, 1H, ^3^ *J*_HH_ = 7.8 Hz, *m*-dipp), 7.12 (d, 1H, ^3^ *J*_HH_ = 7.8 Hz, *m*-dipp), 7.11 (t, 1H, ^3^ *J*_HH_ = 7.6 Hz, *p*-C_6_H_3_), 7.06 (d, 1H, ^3^ *J*_HH_ = 7.6 Hz, ^4^ *J*_HH_ = 1.0 Hz, *m*-C_6_H_3_), 6.76 (ddd, 1H, ^3^ *J*_HH_ = 7.6 Hz, ^4^ *J*_HP_ = 2.9 Hz, ^4^ *J*_HH_ = 1.0 Hz, *m*-C_6_H_3_), 6.26 (d, ^3^ *J*_HH_ = 8.3 Hz, 1H, *m*-dipp′), 6.10 (d, 1H, ^3^ *J*_HH_ = 6.5 Hz, *m*-dipp′), 6.01 (dd, 1H, ^3^ *J*_HH_ = 8.3 Hz, ^3^ *J*_HH_ = 6.5 Hz, *p*-dipp′), 2.98 (sept, 1H, ^3^ *J*_HH_ = 6.8 Hz, C*H*(CH_3_)_2_), 2.55 (sept, 1H, ^3^ *J*_HH_ = 6.5 Hz, C*H*(CH_3_)_2_), 2.05 (sept, 1H, ^3^ *J*_HH_ = 6.7 Hz, C*H*(CH_3_)_2_′) 1.47 (d, 6H, ^2^ *J*_HP_ = 10.2 Hz, PMe), 1.43 (d, 15H, ^4^ *J*_HP_ = 1.4 Hz, C_5_Me_5_), 1.22 (m, 6H, overlapped CH­(C*H*_3_)_2_), 1.15 (m, 6H, overlapped CH­(C*H*_3_)_2_, PMe), 1.10 (m, 6H, overlapped CH­(C*H*_3_)_2_), 0.99 (m, 6H, overlapped CH­(C*H*_3_)_2_′), 0.80 (d, 3H, ^3^ *J*_HH_ = 6.7 Hz, CH­(*CH*_3_)_2_′), 0.51 (sept, 1H, ^3^ *J*_HH_ = 6.8 Hz, C*H*(CH_3_)_2_′), 0.19 (d, 3H, ^3^ *J*_HH_ = 6.7 Hz, CH­(C*H*_3_)_2_′) ppm. ^**13**^ **C­{** ^**1**^ **H} NMR** (100 MHz, CD_2_Cl_2_, 25 °C) δ: 154.8 (d, ^2^ *J*_CP_ = 9 Hz, *o*-C_6_H_3_), 148.1 (s, *o*-dipp), 147.7 (s, *o*-dipp), 146.5 (br, overlapped *o*-C_6_H_3_), 140.2 (br, *ipso*-dipp), 138.2 (br, *o*-dipp′), 136.6 (d, ^1^ *J*_CP_ = 52 Hz, *ipso*-C_6_H_3_), 133.2 (s, *m*-dipp′), 131.2 (d, ^2^ *J*_CP_ = 13 Hz, *m*-C_6_H_3_), 129.4 (br, *m*-C_6_H_3_), 128.4 (s, *p*-dipp), 127.2 (s, *p*-C_6_H_3_), 122.4 (s, *m*-dipp), 122.1 (s, *m*-dipp), 114.8 (s, *p*-dipp′), 107.8 (s, *m*-dipp′), 90.9 (s, *C*_5_Me_5_), 61.3 (br, *ipso*-dipp′), 51.9 (br, *o*-dipp′), 36.7 (s, *C*H­(CH_3_)_2_′), 31.5 (s, *C*H­(CH_3_)_2_), 30.9 (s, *C*H­(CH_3_)_2_), 30.5 (s, *C*H­(CH_3_)_2_′), 26.5 (s, CH­(*C*H_3_)_2_′), 25.7 (s, CH­(*C*H_3_)_2_), 24.6 (s, CH­(*C*H_3_)_2_′), 22.9 (s, CH­(*C*H_3_)_2_), 21.6 (s, CH­(*C*H_3_)_2_′), 21.4 (s, CH­(*C*H_3_)_2_), 21.3 (s, CH­(*C*H_3_)_2_), 21.2 (s, CH­(*C*H_3_)_2_), 18.2 (d, ^1^ *J*_CP_ = 38 Hz, PMe), 15.4 (d, ^1^ *J*_CP_ = 34 Hz, PMe), 9.2 (s, C_5_ *Me*_5_) ppm. ^**31**^ **P­{** ^**1**^ **H} NMR** (125 MHz, CD_2_Cl_2_, 25 °C) δ: 3.4 ppm.

### Single-Crystal X-ray Diffraction (XRD)

Low-temperature diffraction data were collected on a Bruker APEX-II CCD diffractometer or a D8 Quest APEX-III single-crystal diffractometer with a Photon III detector and a IμS 3.0 microfocus X-ray source at the Instituto de Investigaciones Químicas, Sevilla. Data were collected by means of ω and φ scans using monochromatic radiation λ­(MoKα) = 0.71073 Å or λ­(AgKα) = 0.56086 Å. The diffraction images collected were processed and scaled using APEX-III software. Using Olex2,^15^ the structures were solved with SHELXT or SHELXS and were refined against F^2^ on all data by full-matrix least-squares with SHELXL.^16^ All non-hydrogen atoms were refined anisotropically. Hydrogen atoms were included in the model at geometrically calculated positions and refined using a riding model. The isotropic displacement parameters of all hydrogen atoms were fixed to 1.2 times the U value of the atoms to which they are linked (1.5 times for methyl groups).

### Computational Studies

Calculations were performed at the DFT level with the Gaussian 09 (Revision E.01) program.^17^ The hybrid functional PBE0 ^18^ was used throughout all computational studies. Dispersion effects were accounted for by using Grimme’s D3 parameter set with Becke–Johnson (BJ) damping.^19^ Geometry optimizations were carried out without geometry constraints, using the 6–31G­(d,p)^20^ basis set to represent the C, H, P, Cl, O, Mg, F, and S atoms and the Stuttgart/Dresden Effective Core Potential and its associated basis set (SDD)^21^ to describe the Ir atom. Bulk solvent effects (dichloromethane or diethyl ether) were included at the optimization stage with the SMD continuum model.^22^ The stationary points and their nature as minima or saddle points (TS) were characterized by vibrational analysis, which also produced enthalpy (H), entropy (S) and Gibbs energy (G) data at 298.15 K. The minima connected by a given transition state were determined by perturbing the transition states along the TS coordinate and optimizing to the nearest minimum. QTAIM analyses were performed with the Multiwfn^23^ software on wave functions generated with the Gaussian 09 program. Energy Decomposition calculations have been performed with the ADF program.^24^ Activation Strain Analysis was used to provide insight into the factors that govern the height of the reaction.^25^

## Supplementary Material





## Notes

Computational coordinates (XYZ)
Experimental procedures; NMR spectra for all new compounds; XRD for 1, 2, 3, 3′, and 8; computational studies (PDF)
