# Supplementary material for: Bulky Terphenyl Phosphines Stabilize Otherwise Highly Reactive Iridium Fragments, Key in C–H Activation Reactions
Source: Inorg Chem. 2026 Jul 10;65(29):16869–78. doi: 10.1021/acs.inorgchem.6c01943 (PMC13418093; doi:10.1021/acs.inorgchem.6c01943)
Supplement: Supplementary file 2 [file ic6c01943_si_002.pdf]

Supporting Information

Bulky Terphenyl Phosphines Stabilize Otherwise Highly Reactive Iridium Fragments Key in C–H Activation Reactions

Alejandra Pita-Milleiro[+], Martina Landrini[+], Miquel Navarro, Juan J. Moreno, Jefferson Guzmán, Celia Maya, Jesús Campos*

[+]*These authors contributed equally to this work*

Instituto de Investigaciones Químicas (IIQ), Departamento de Química Inorgánica and Centro de Innovación en Química Avanzada (ORFEO-CINQA), Universidad de Sevilla and Consejo Superior de Investigaciones Científicas (CSIC), Avenida Américo Vespucio 49, 41092 Sevilla, Spain.email: jesus.campos@iiq.csic.es

1. NMR Spectra 2

2. X-Ray Structural Characterization of complexes 32

3. Computational Chemistry 35

 3.1 Formation of 5 via oxidative addition 36

 3.2 Elimination of CH_4 from 4 via formation of a fulvene species 37

 3.3 Formation of 7 38

 3.4 Elimination of ethane from 7 39

 3.5 Formation of 6 from 4 40

 3.6 Formation of 6 from 5 41

 3.7 Activation Strain Analysis of the formation of Ir-Me species 42

 3.8 Interaction energy and torsion angle correlation 42

 3.9 Topological studies 43

4. References 48

1. NMR Spectra

1

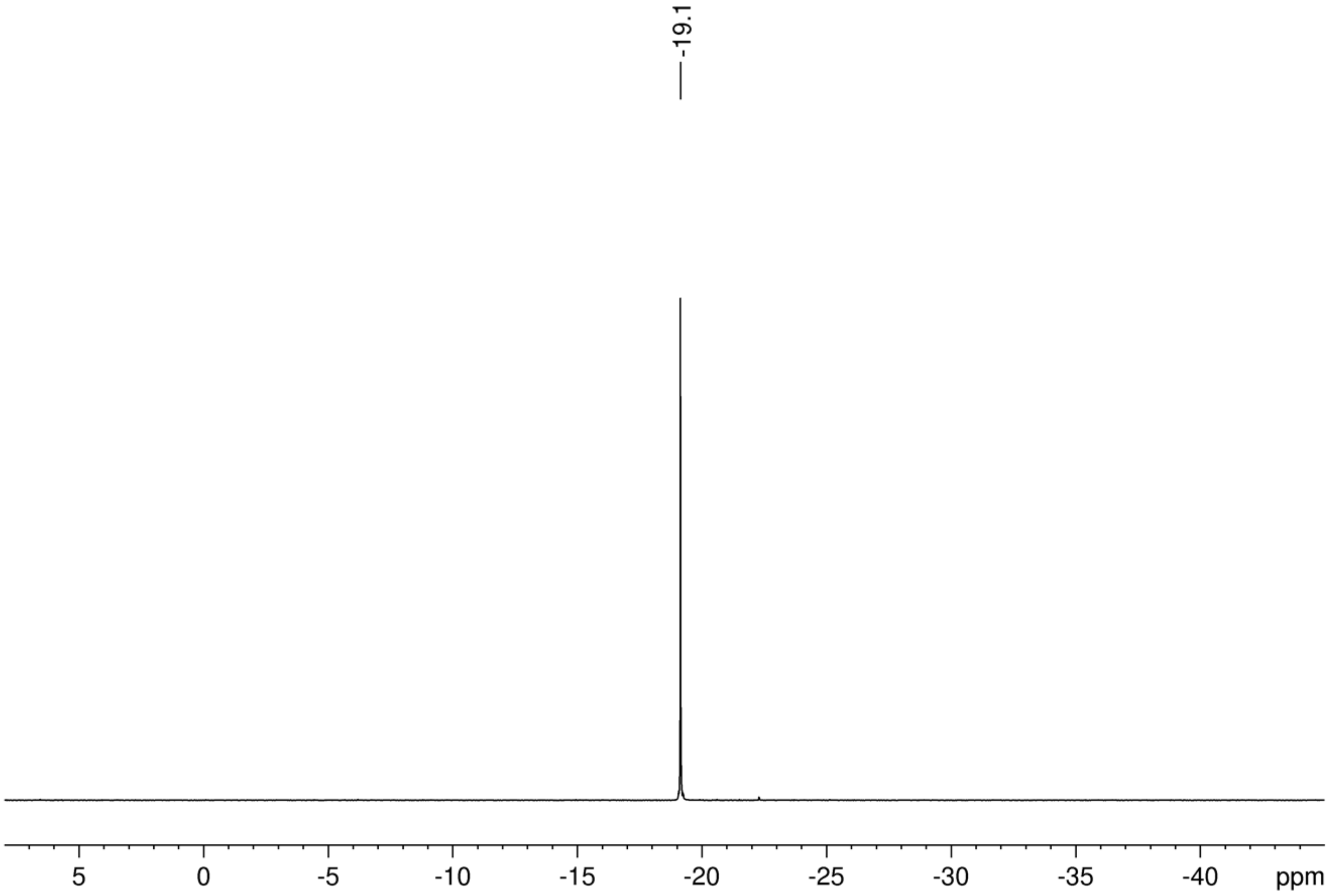

Figure S1. *$^{31}P\{^{1}H\}$ NMR (202 MHz, CD_2Cl_2, 25 °C). Complex* ***1***.

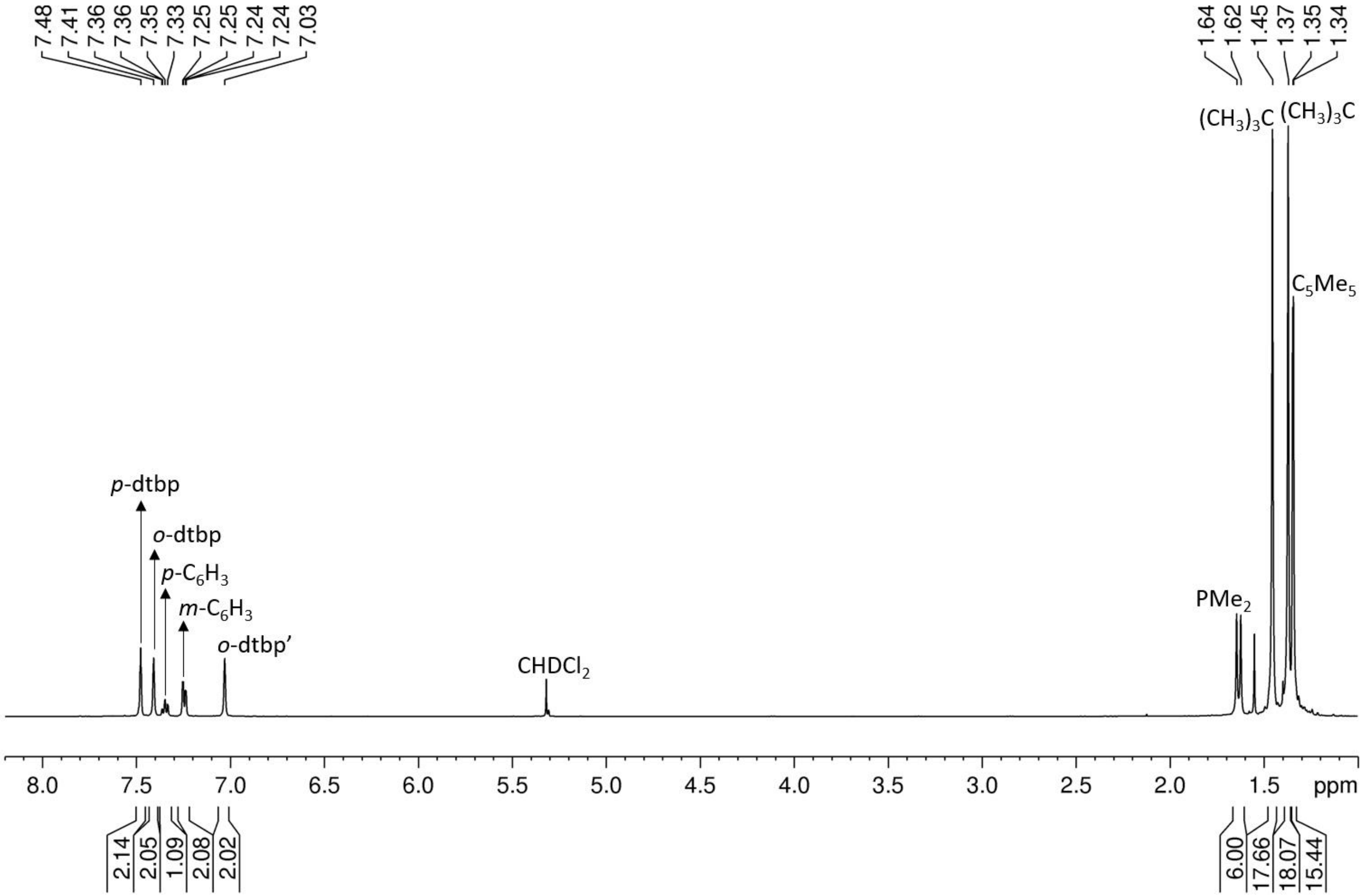

Figure S2. *^{1}H NMR (500 MHz, CD_2Cl_2, 25 °C). Complex **1**.*

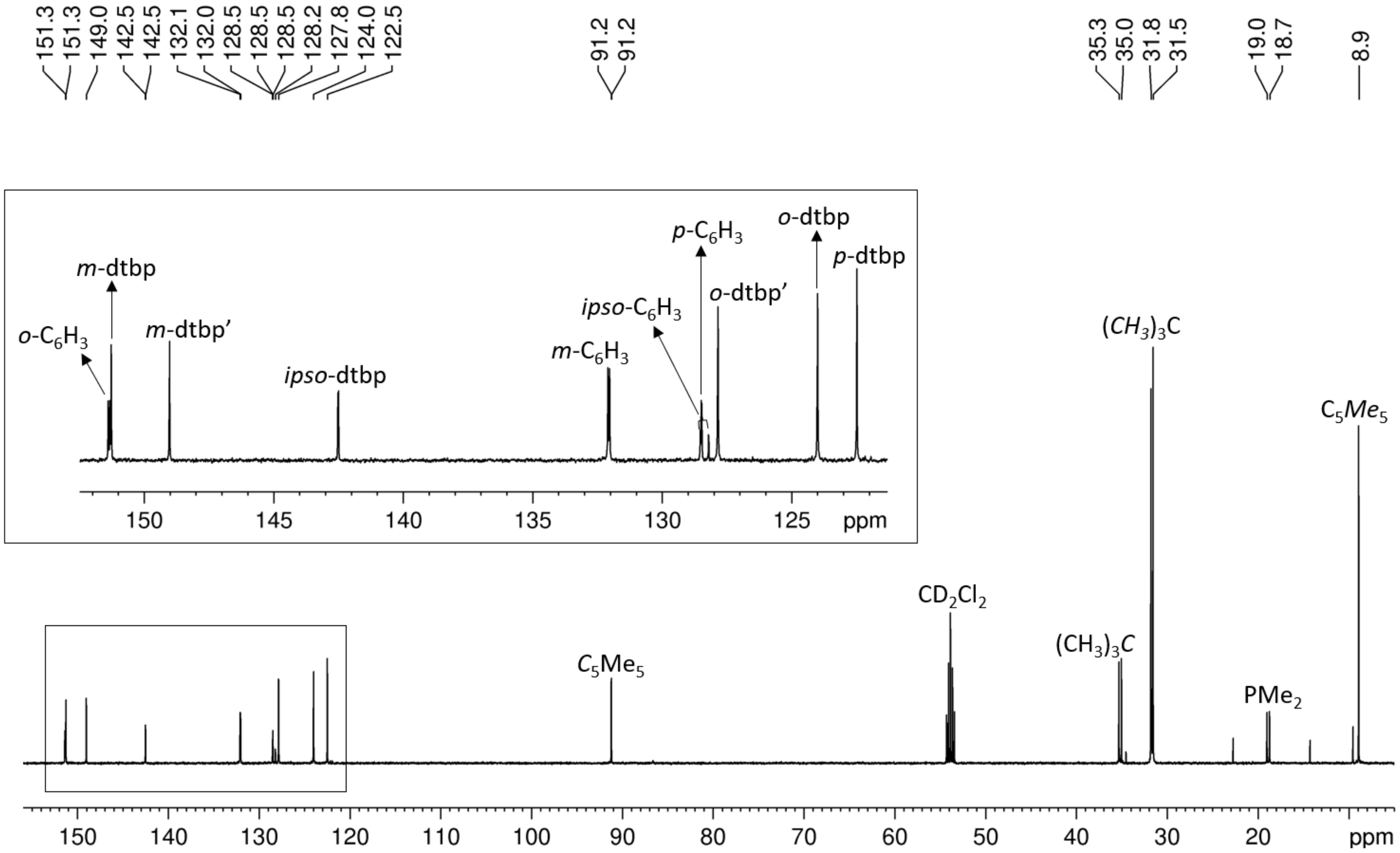

Figure S3. $^{13}C\{^1H\}$ *NMR (125 MHz, CD_2Cl_2, 25 °C). Complex* ***1***.

2

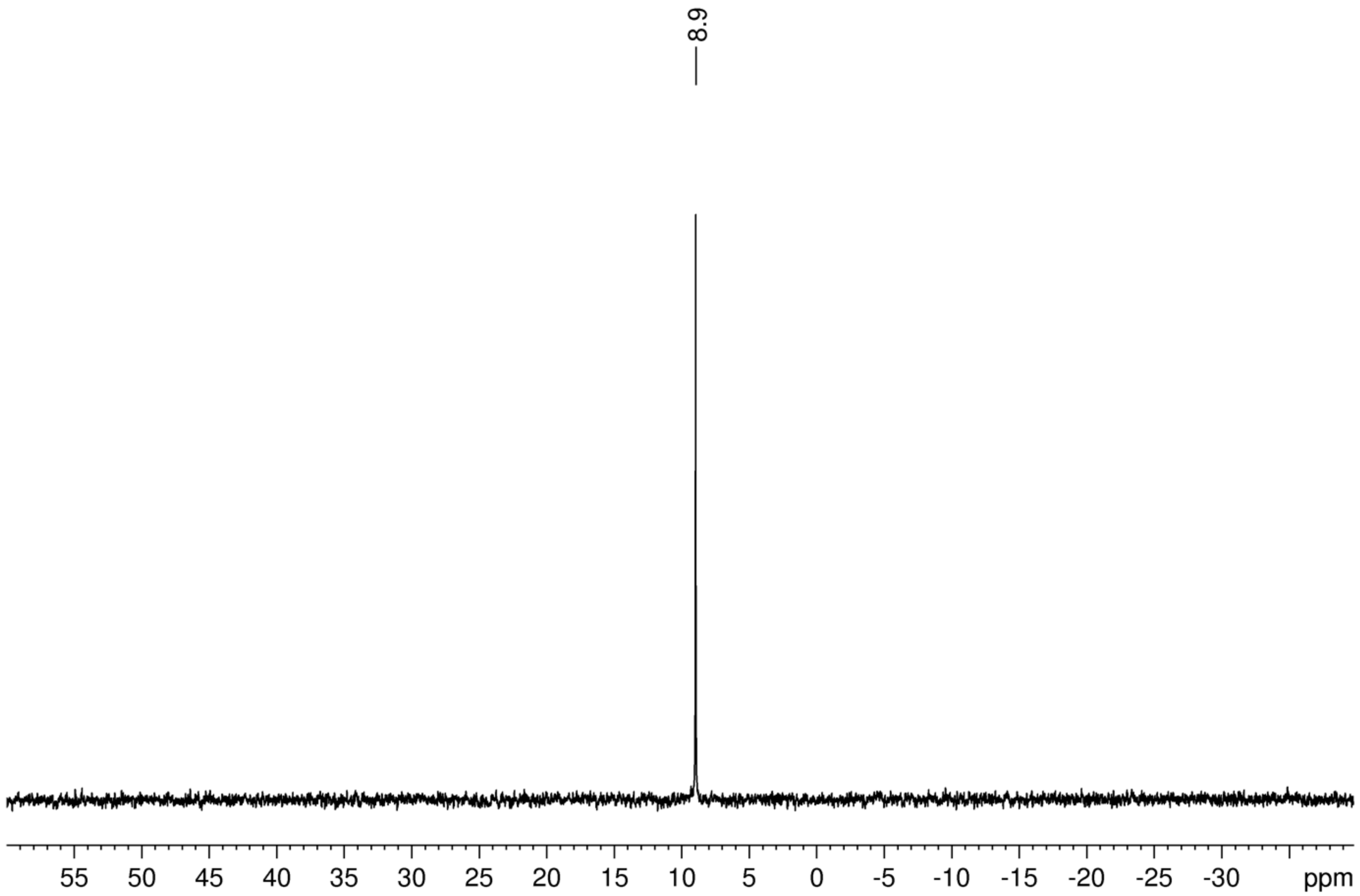

Figure S4. *$^{31}P\{^{1}H\}$ NMR (202 MHz, CD_2Cl_2, 25 °C). Complex* ***2***.

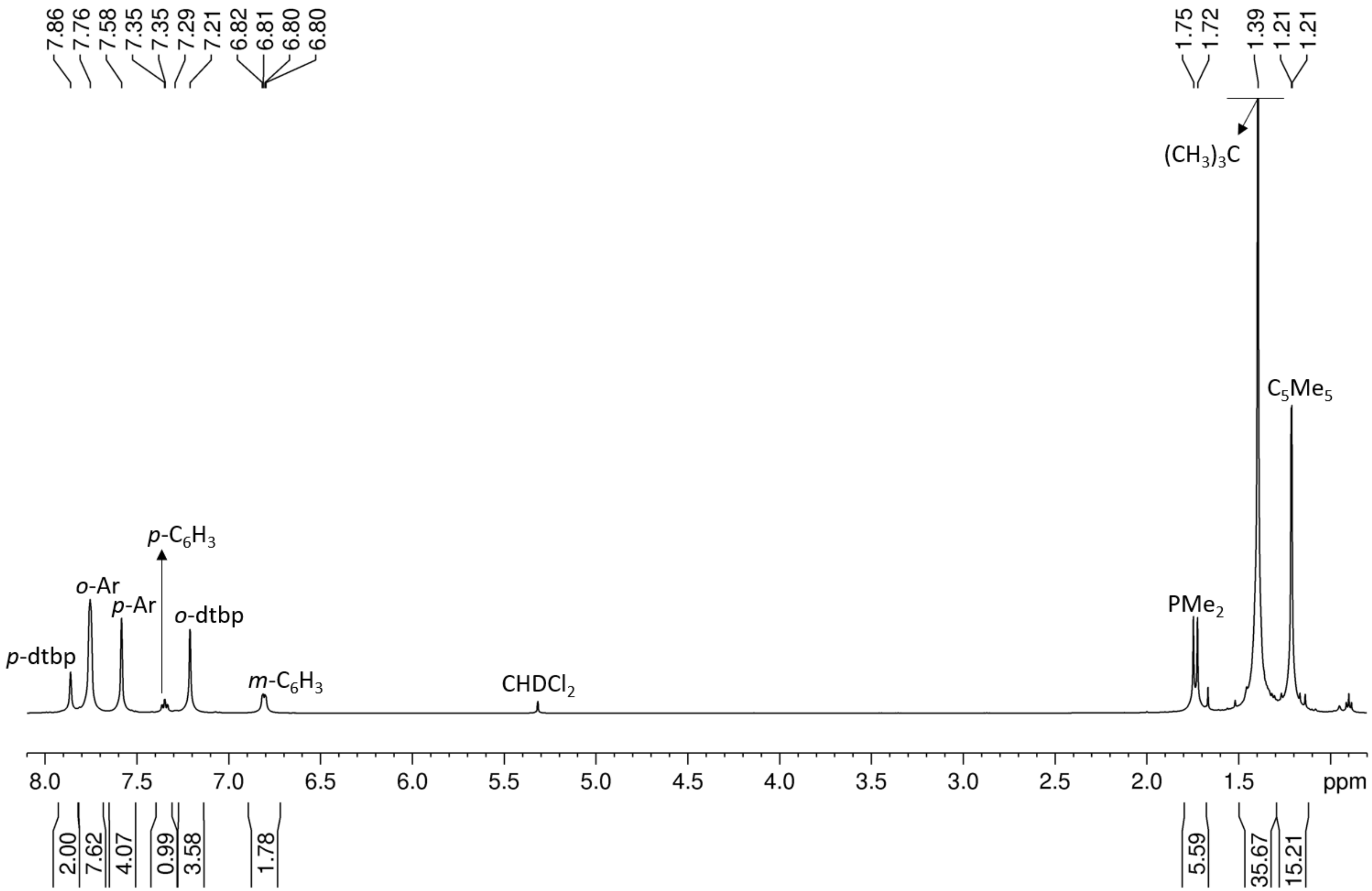

Figure S5. *^{1}H NMR (500 MHz, CD_2Cl_2, 25 °C). Complex* ***2***.

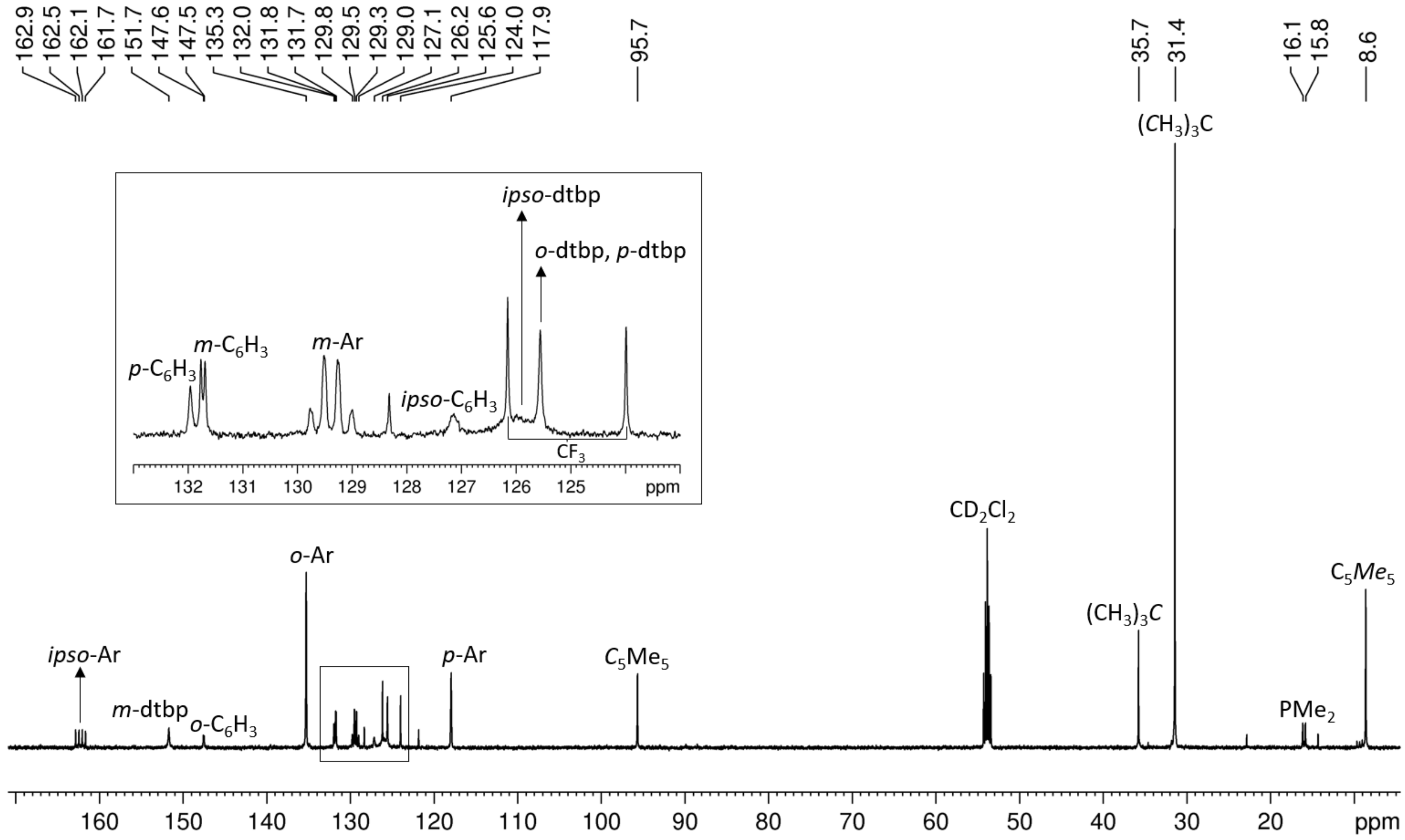

Figure S6. ${}^{13}C\{{}^{1}H\}$ *NMR (125 MHz, CD_2Cl_2, 25 °C). Complex* ***2***.

3

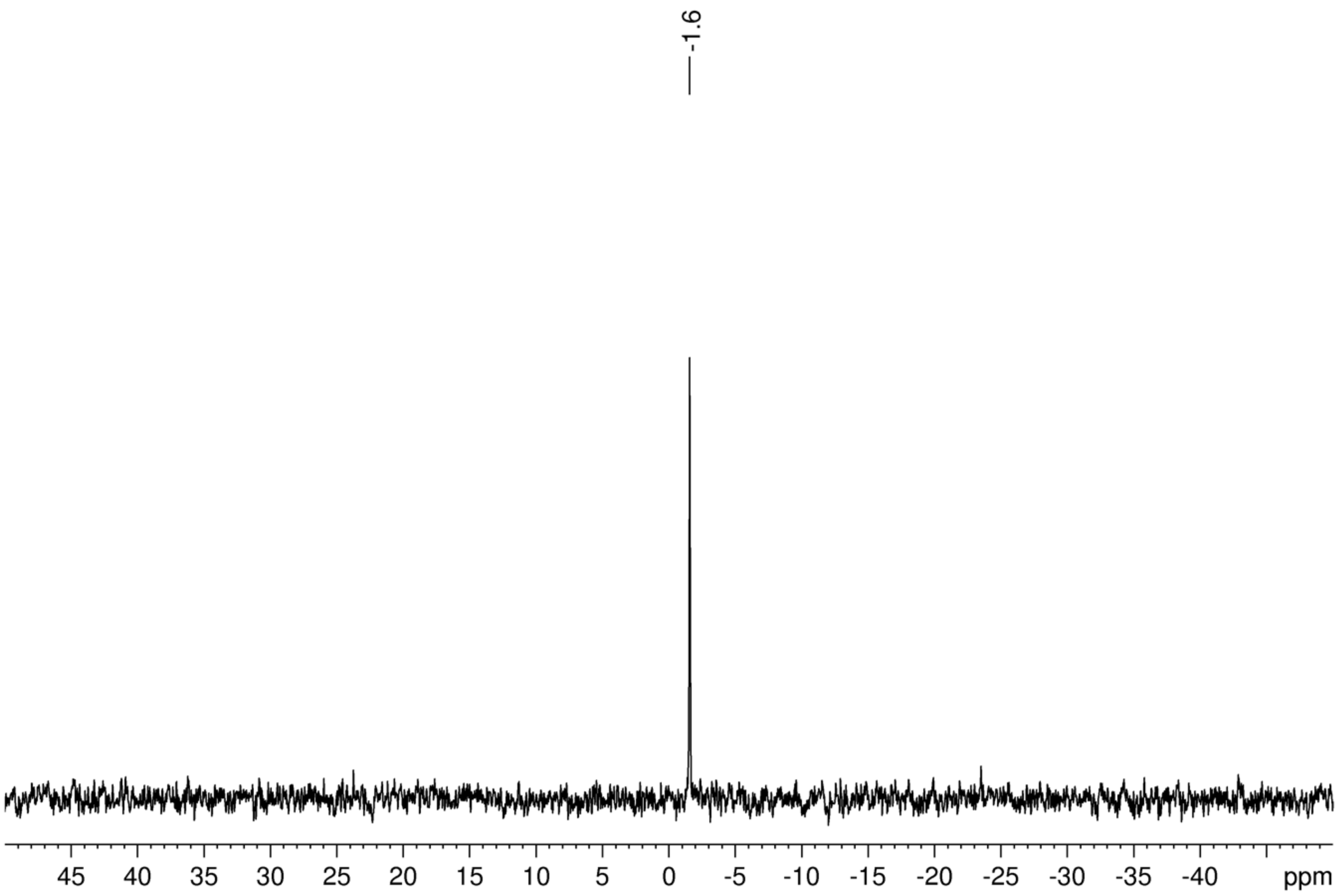

Figure S7. *$^{31}P\{^1H\}$ NMR (162 MHz, CD_2Cl_2, 25 °C). Complex* ***3***.

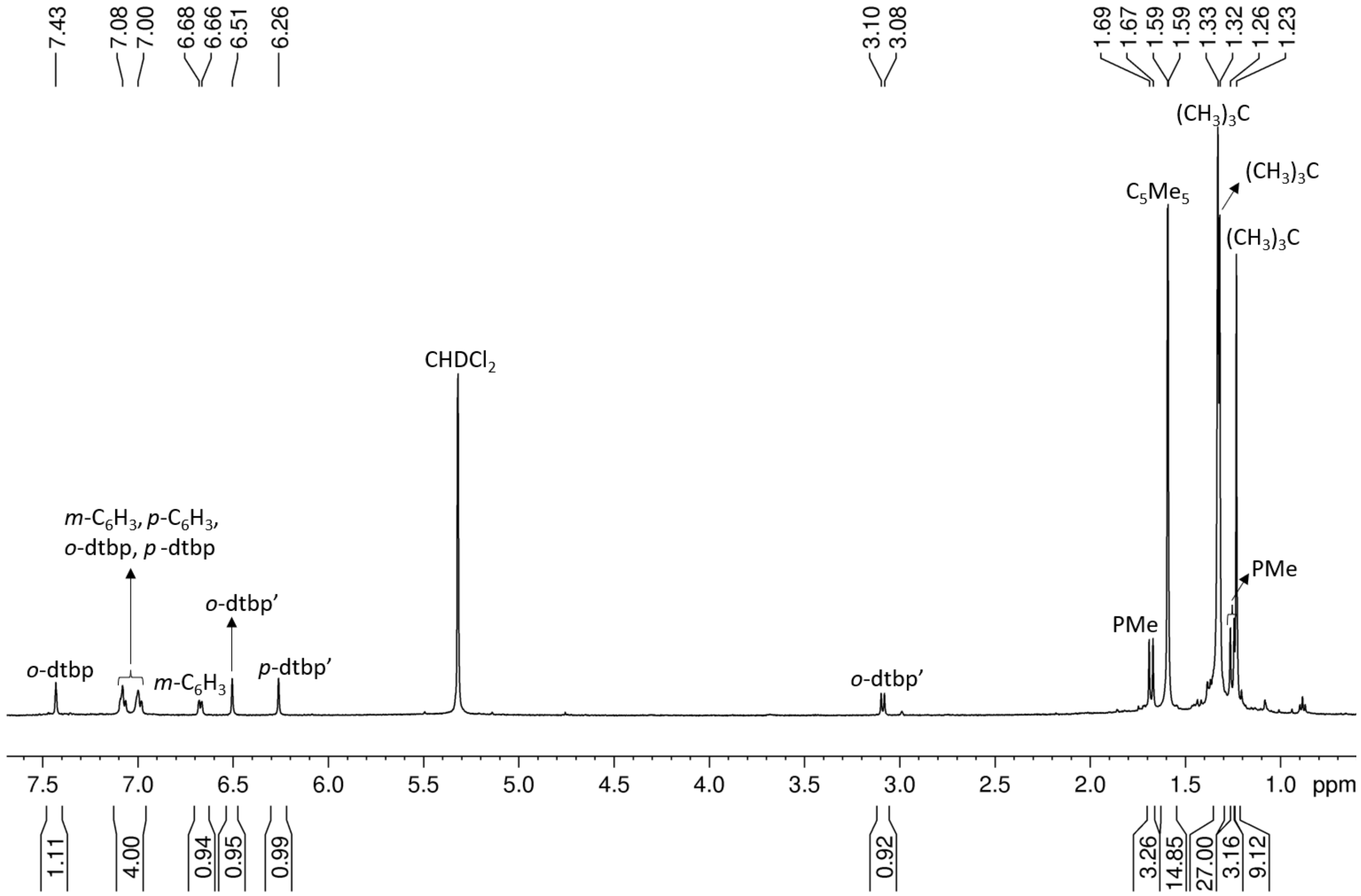

Figure S8. *^{1}H NMR (500 MHz, CD_2Cl_2, 25 °C). Complex **3**.*

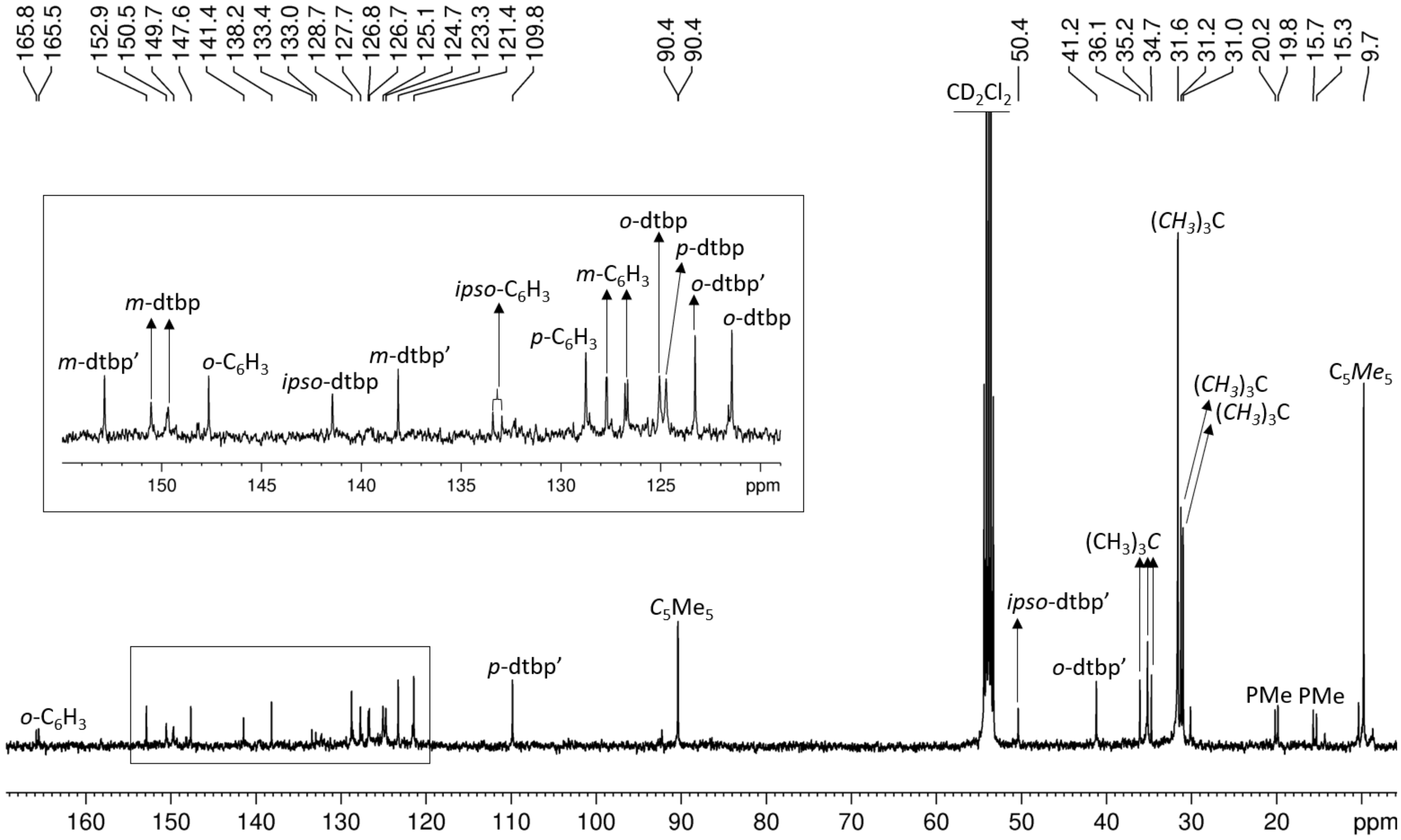

Figure S9. $^{13}C\{^{1}H\}$ *NMR (125 MHz, CD_2Cl_2, 25 °C). Complex **3**.*

4

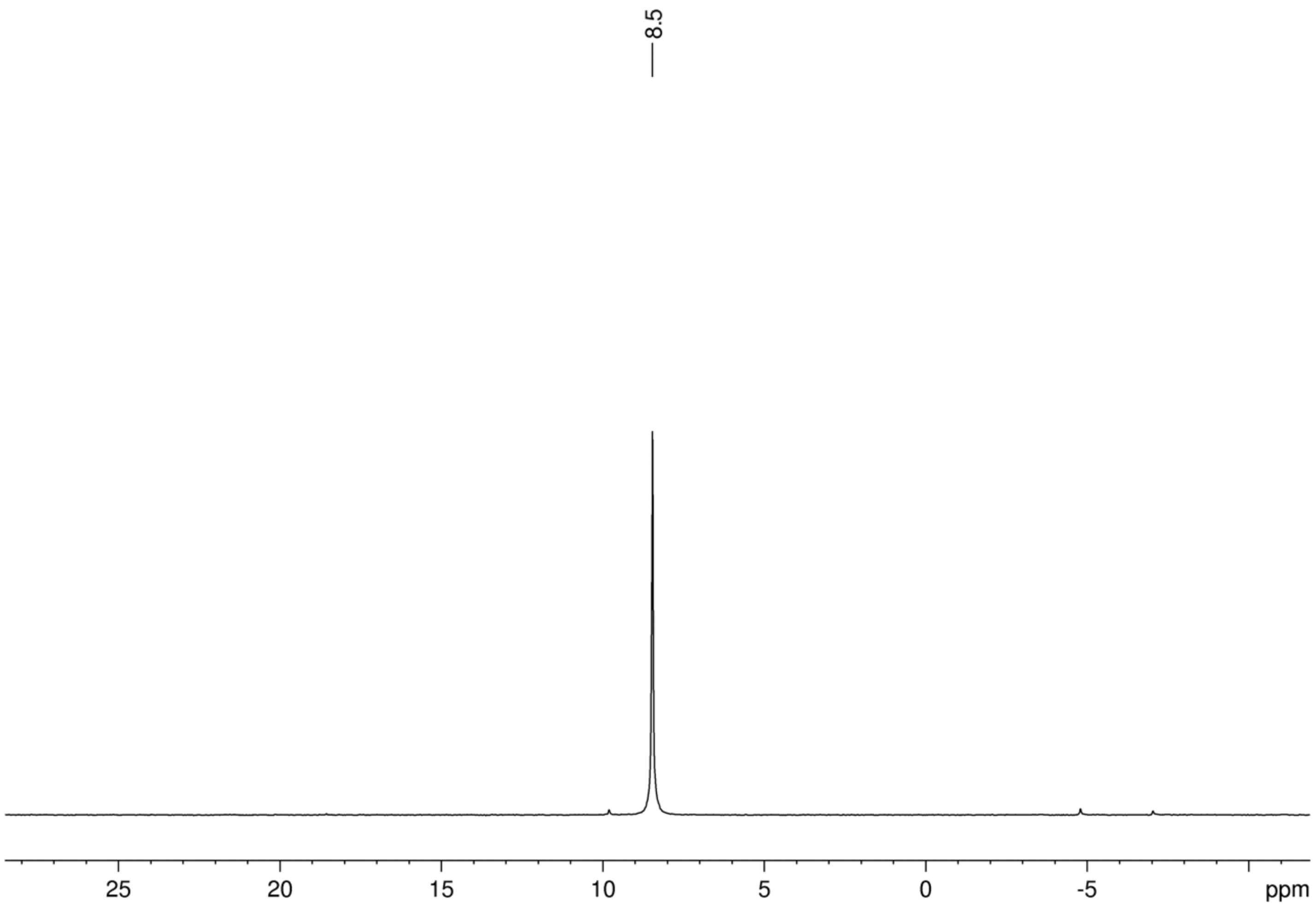

Figure S10. *$^{31}P\{^1H\}$ NMR (162 MHz, CD_2Cl_2, −80 °C). Complex* ***4***.

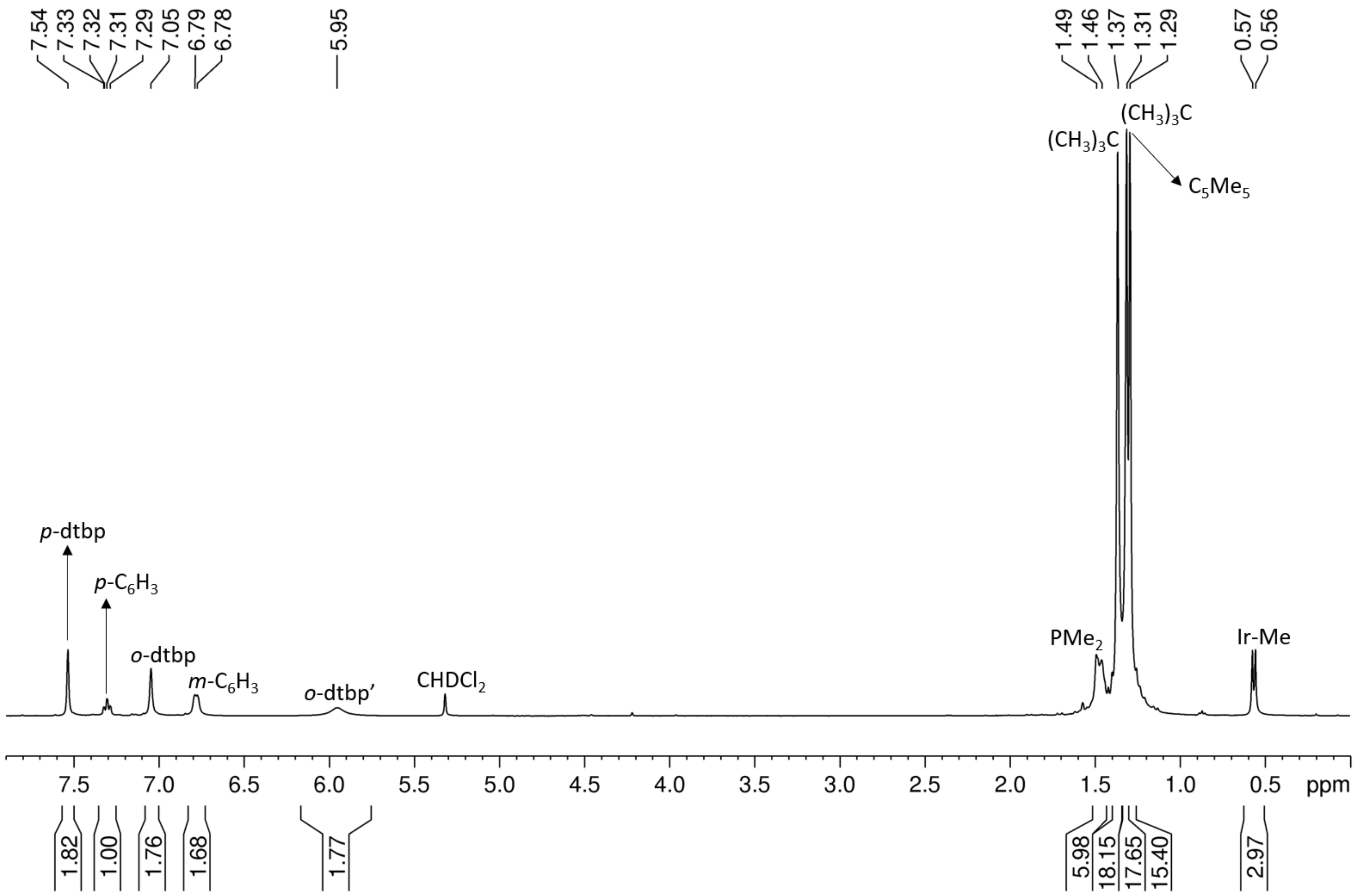

Figure S11. *^{1}H NMR (400 MHz, CD_2Cl_2, 10 °C). Complex* ***4***.

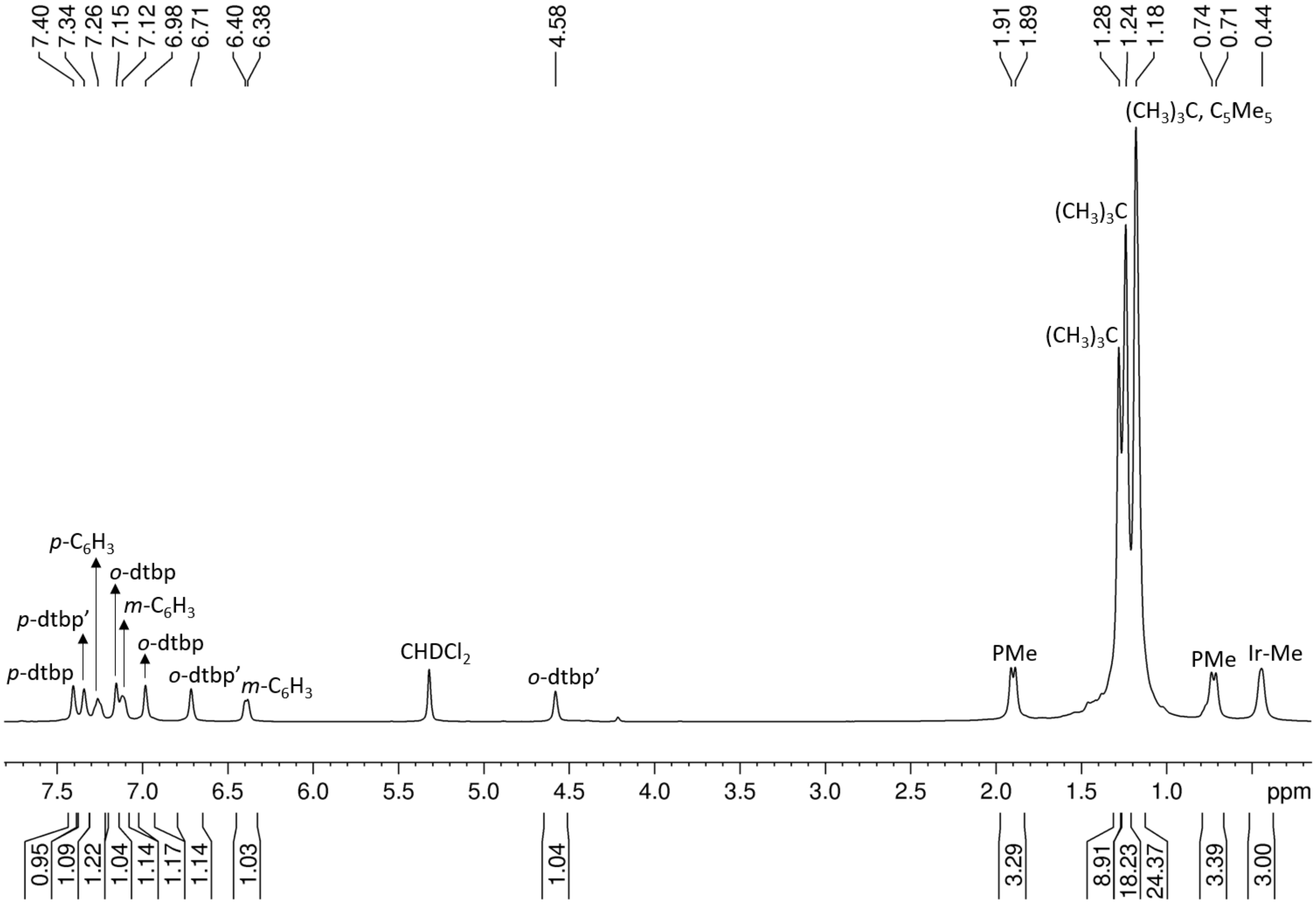

Figure S12. *^{1}H NMR (400 MHz, CD_2Cl_2, −80 °C). Complex* ***4***.

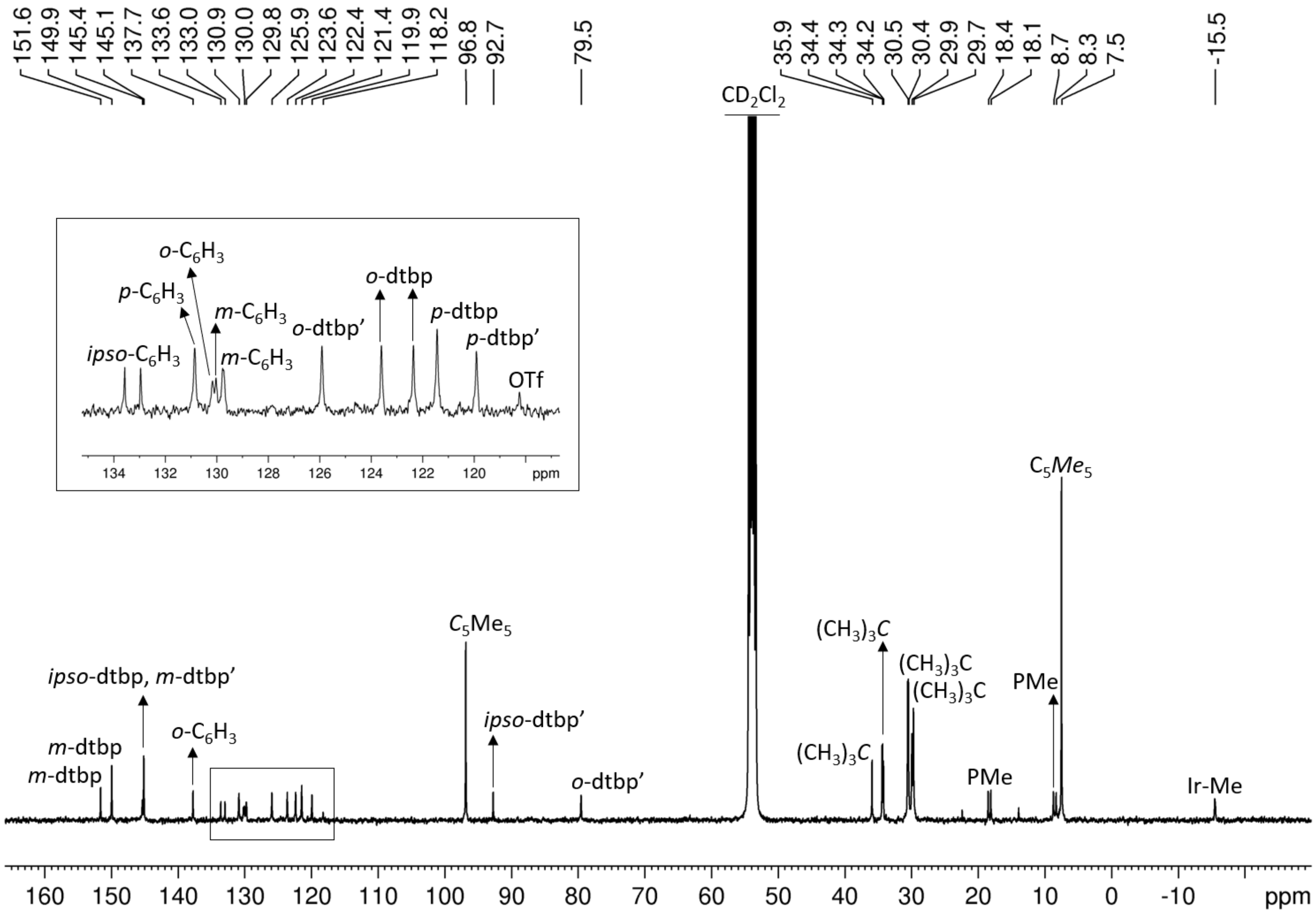

Figure S13. $^{13}C\{^1H\}$ *NMR (100 MHz,* CD_2Cl_2*, −80 °C). Complex* ***4***.

5

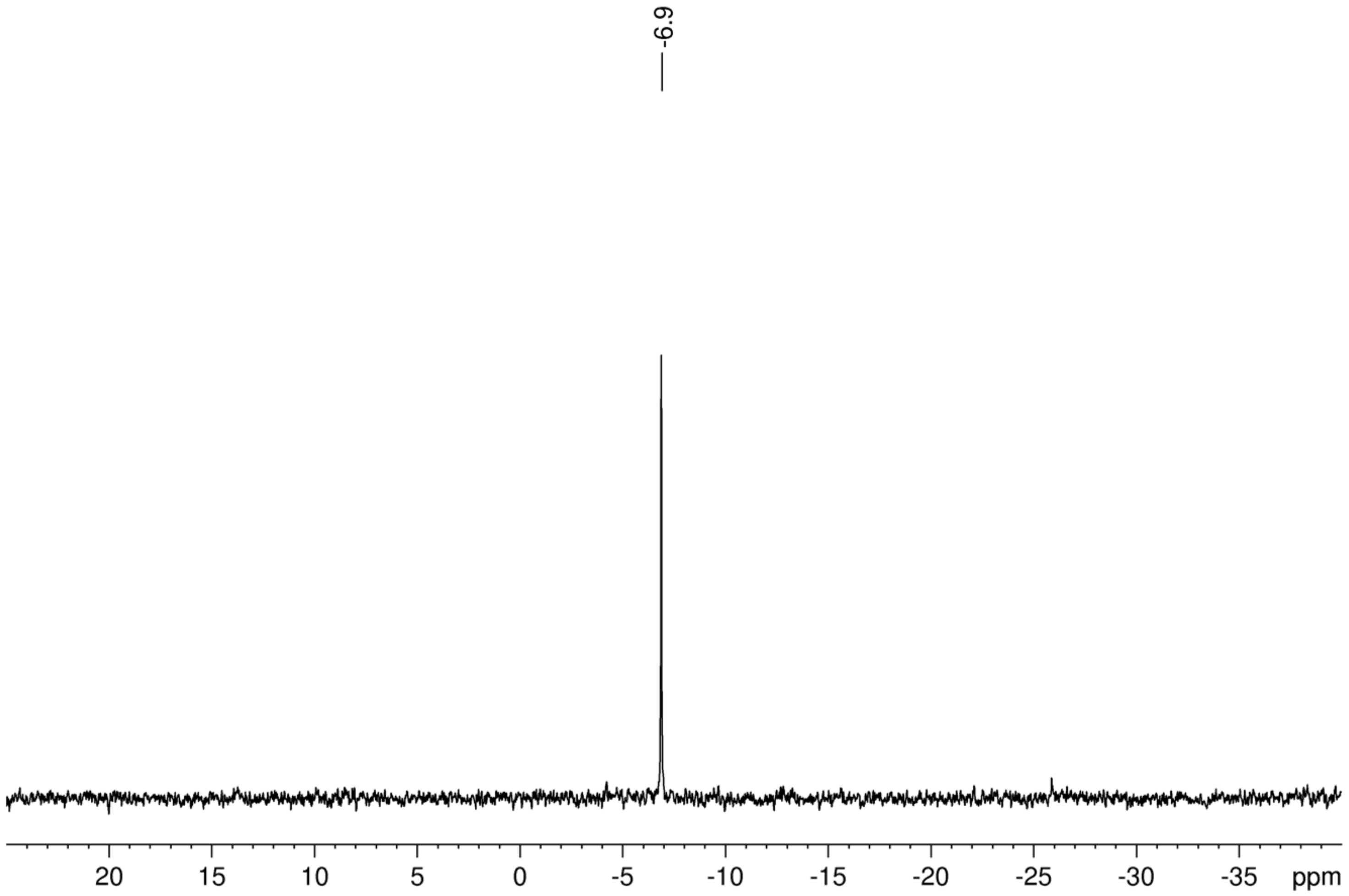

Figure S14. *$^{31}P\{^1H\}$ NMR (162 MHz, CD_2Cl_2, 25 °C). Complex* ***5***.

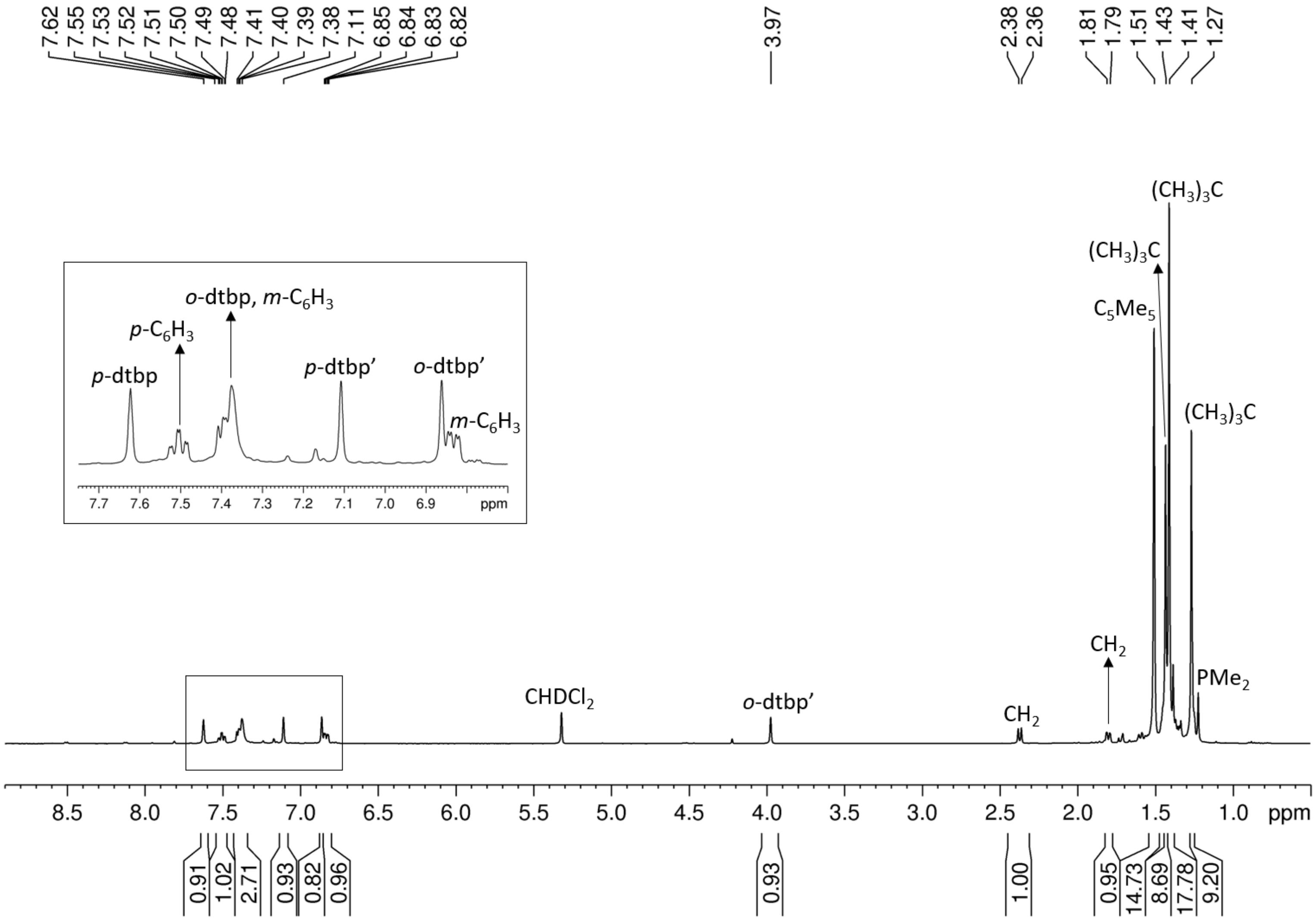

Figure S15. *^{1}H NMR (500 MHz, CD_2Cl_2, 25 °C). Complex* ***5***.

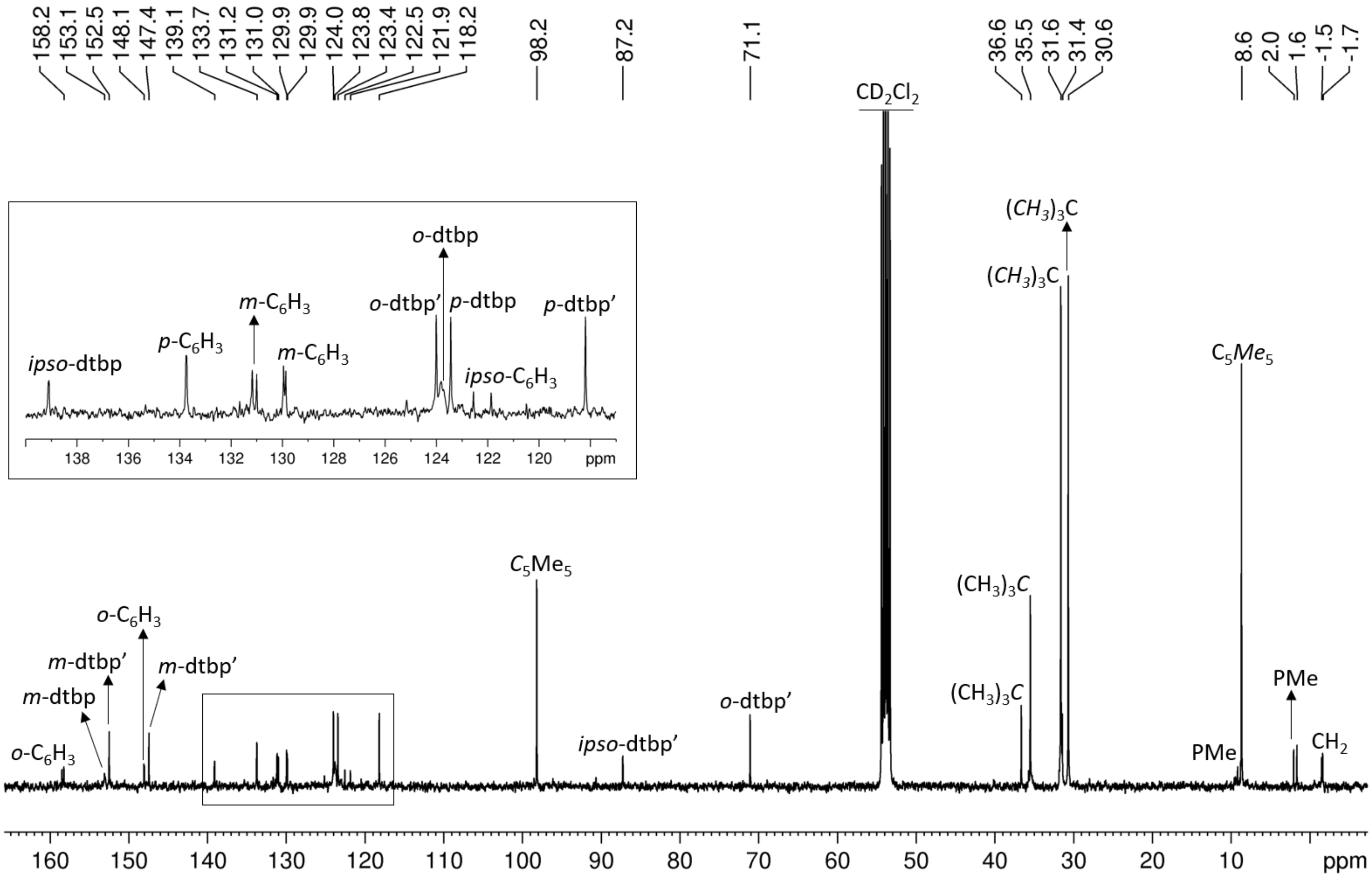

Figure S16. *$^{13}C\{^{1}H\}$ NMR (100 MHz, CD_2Cl_2, 25 °C). Complex* ***5***

6

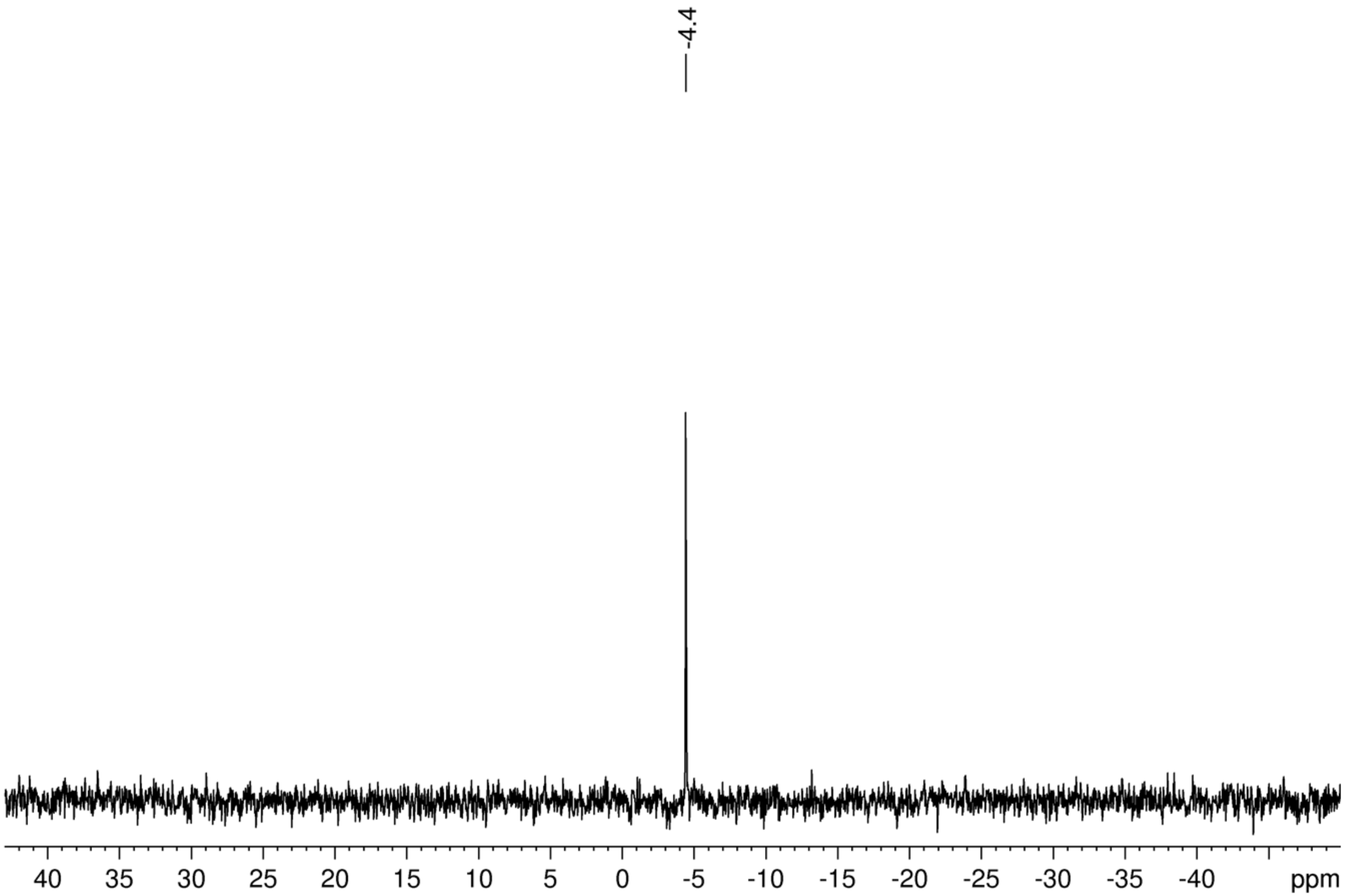

Figure S17**. $^{31}P\{^{1}H\}$ NMR (202 MHz, CD_2Cl_2, 25 °C). Complex **6

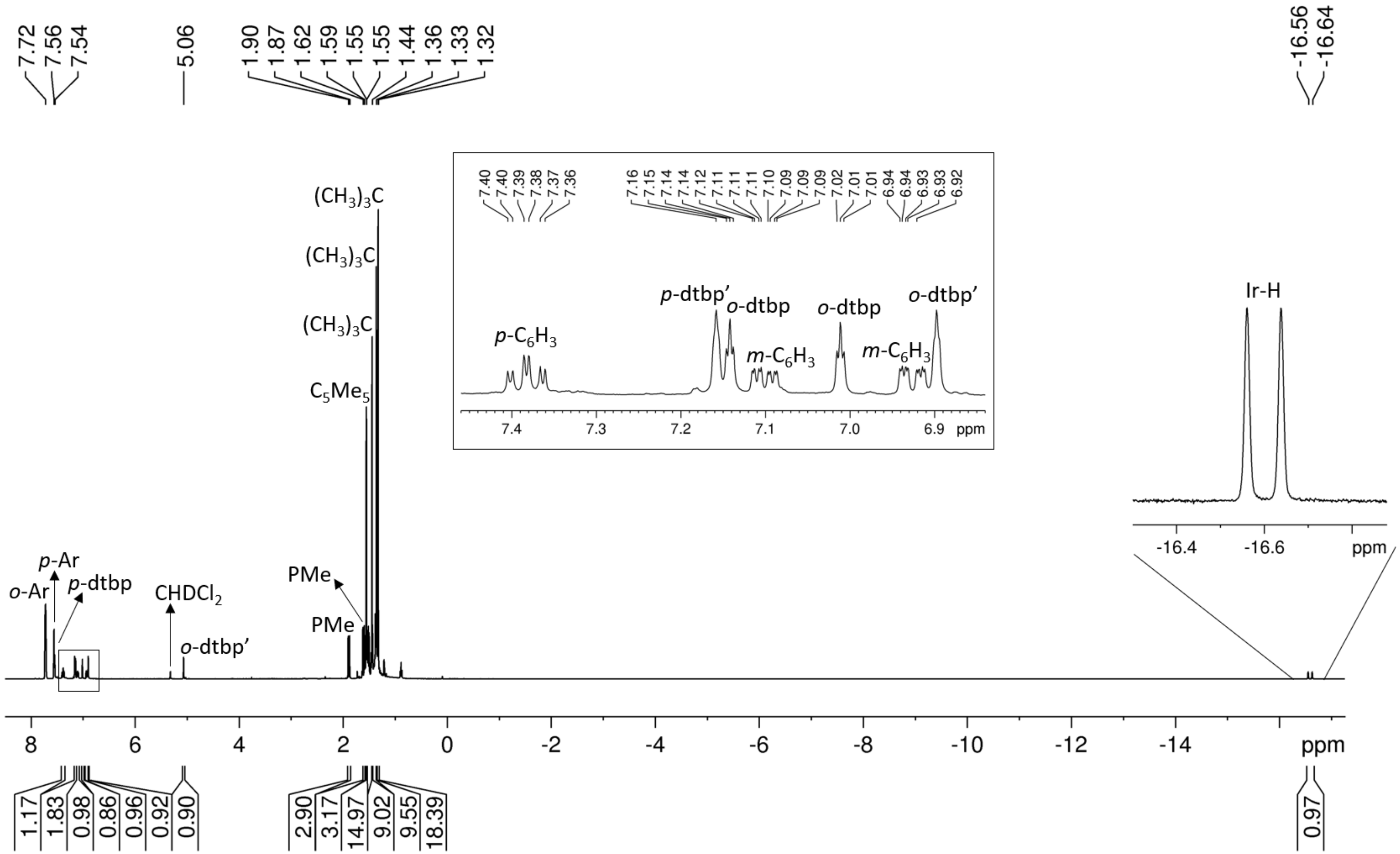

Figure S18**. ^{1}H NMR (500 MHz, CD_2Cl_2, 25 °C). Complex **6

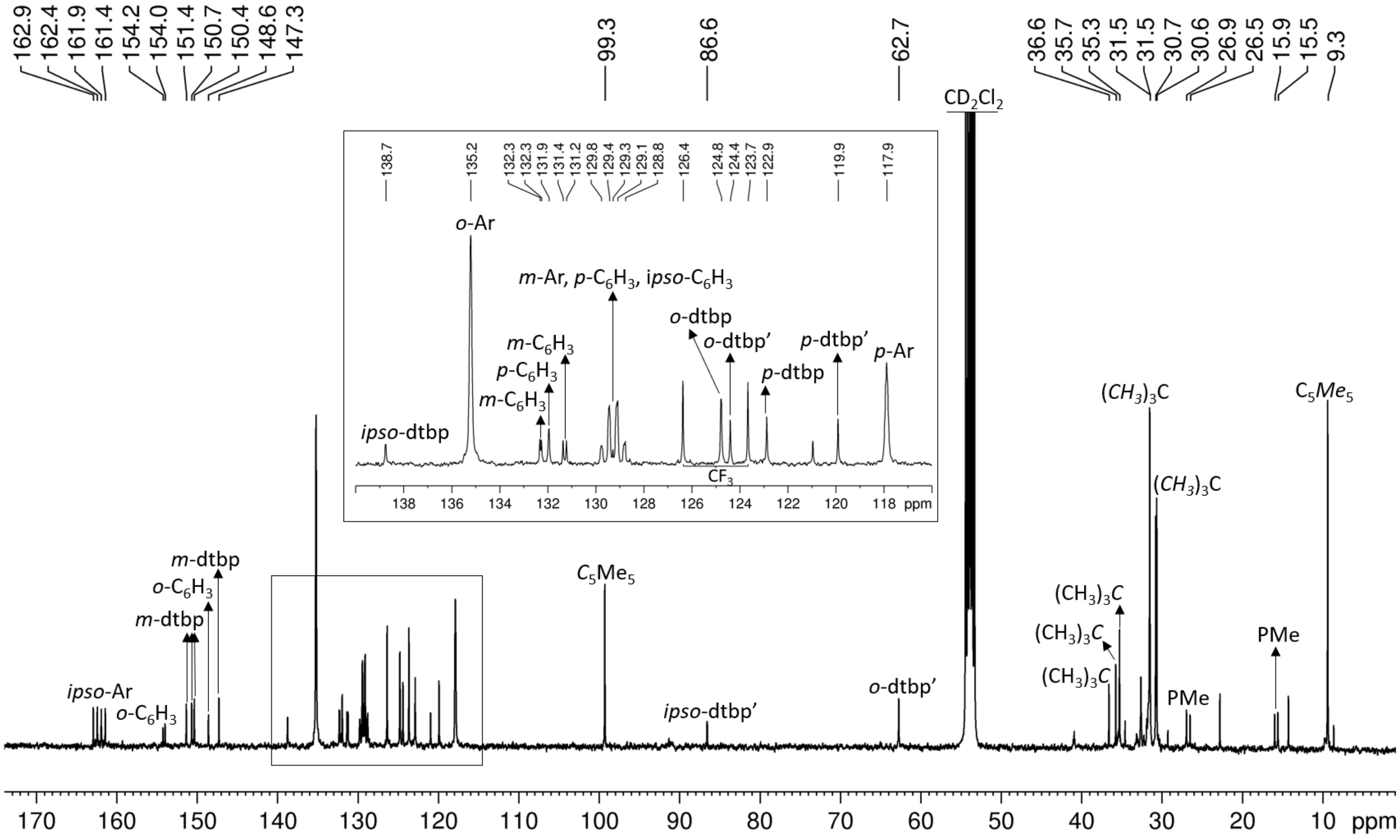

Figure S19. $^{13}C\{^1H\}$ *NMR (125 MHz,* CD_2Cl_2*, 25 °C). Complex* ***6***.

7

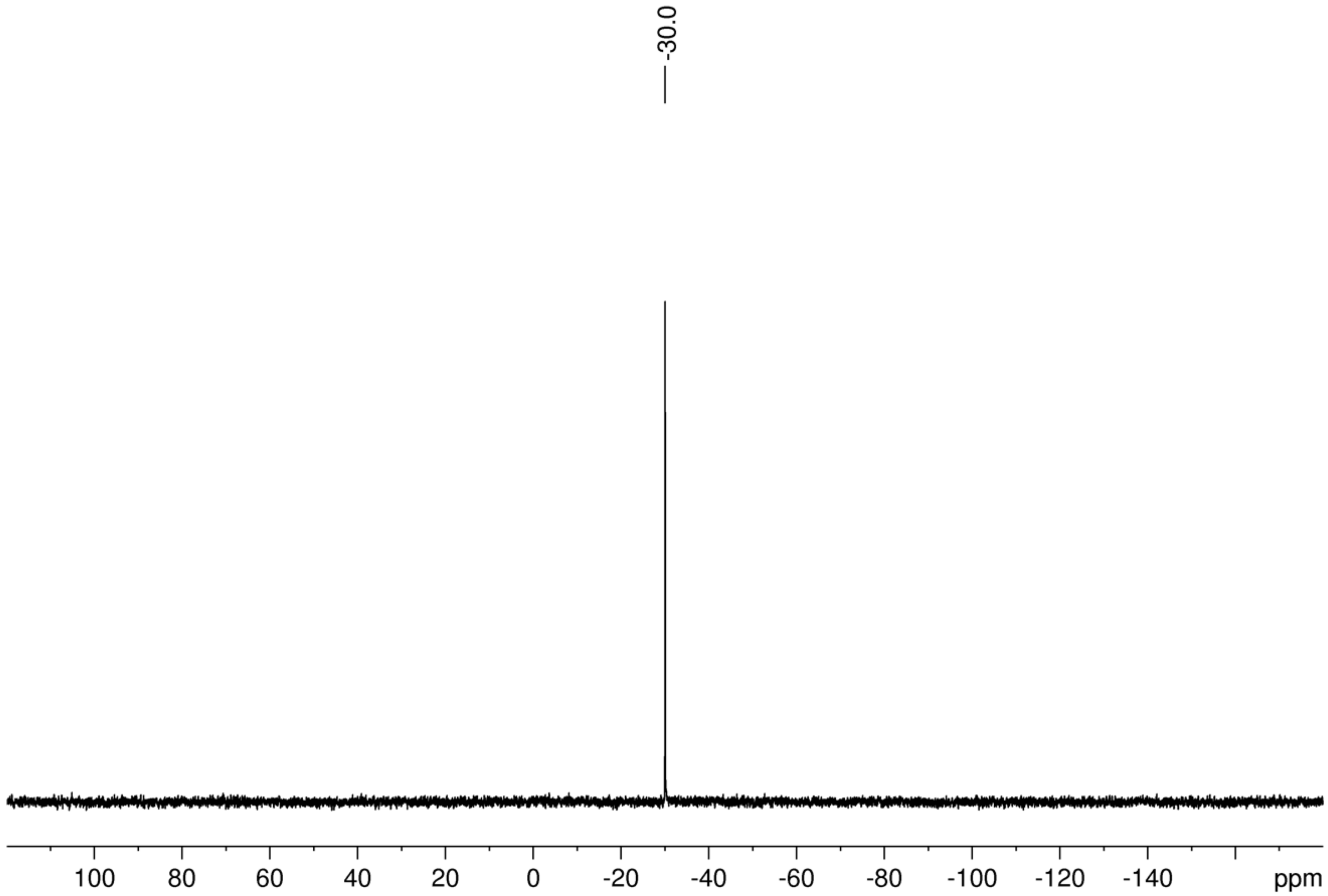

Figure S20. *$^{31}P\{^{1}H\}$ NMR (202 MHz, CD_2Cl_2, 25 °C). Complex 7.*

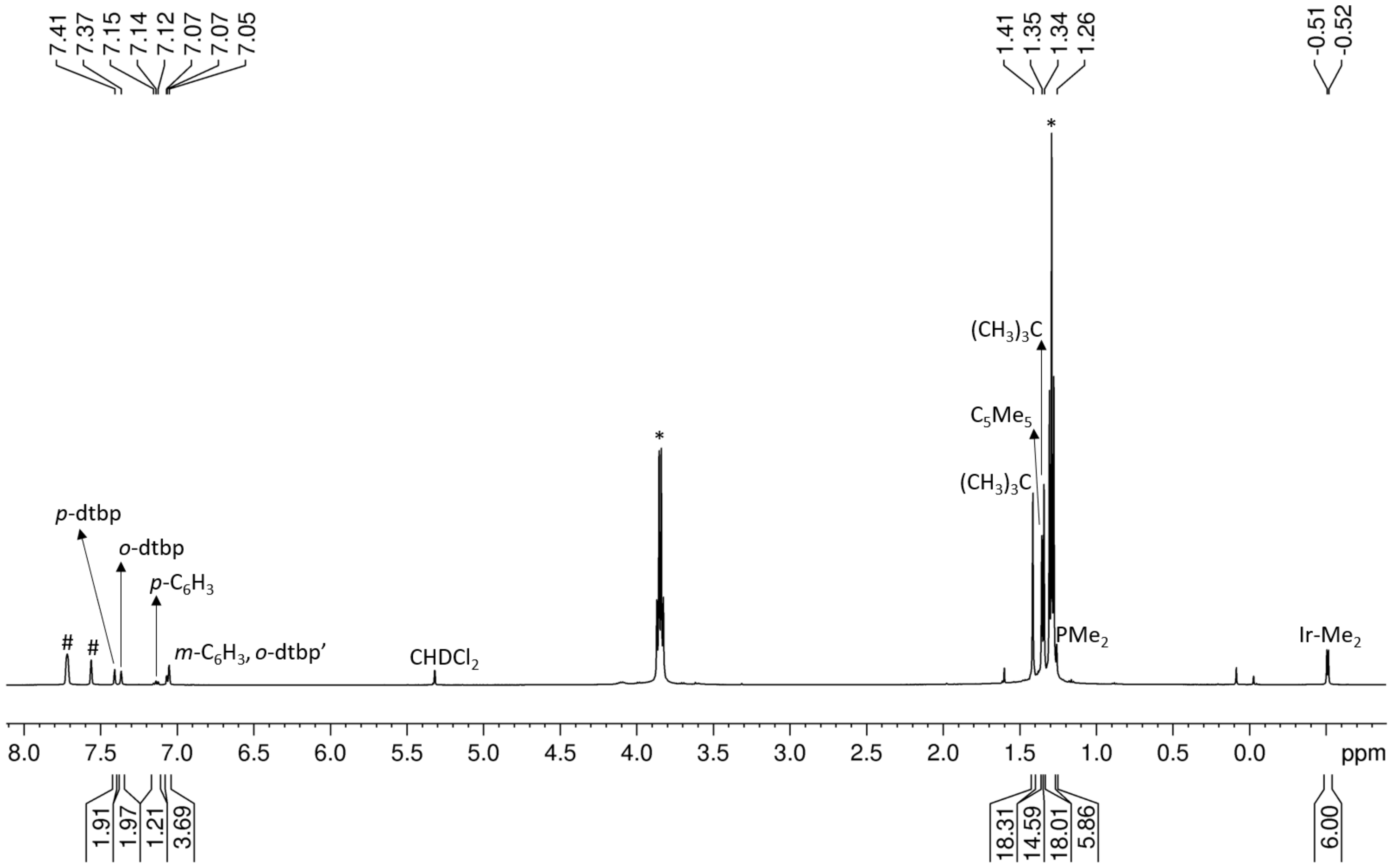

Figure S21. *^{1}H NMR (500 MHz, CD_2Cl_2, 25 °C). Complex **7** (# Denotes BArF and * Denotes Et_2O).*

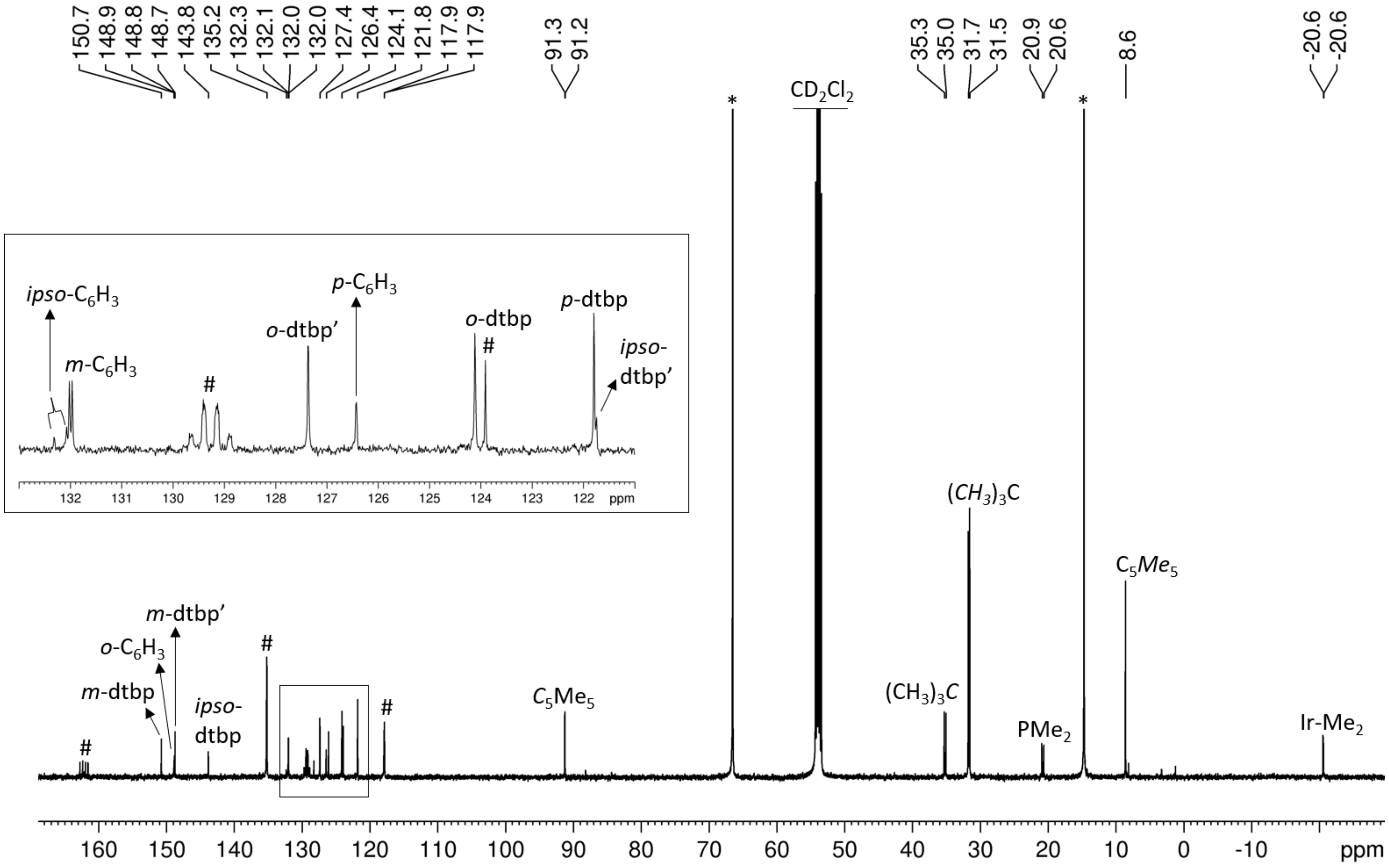

Figure S22. *$^{13}C\{^1H\}$ NMR (125 MHz, CD_2Cl_2, 25 °C). Complex **7** (# Denotes BArF and * Denotes Et_2O).*

8

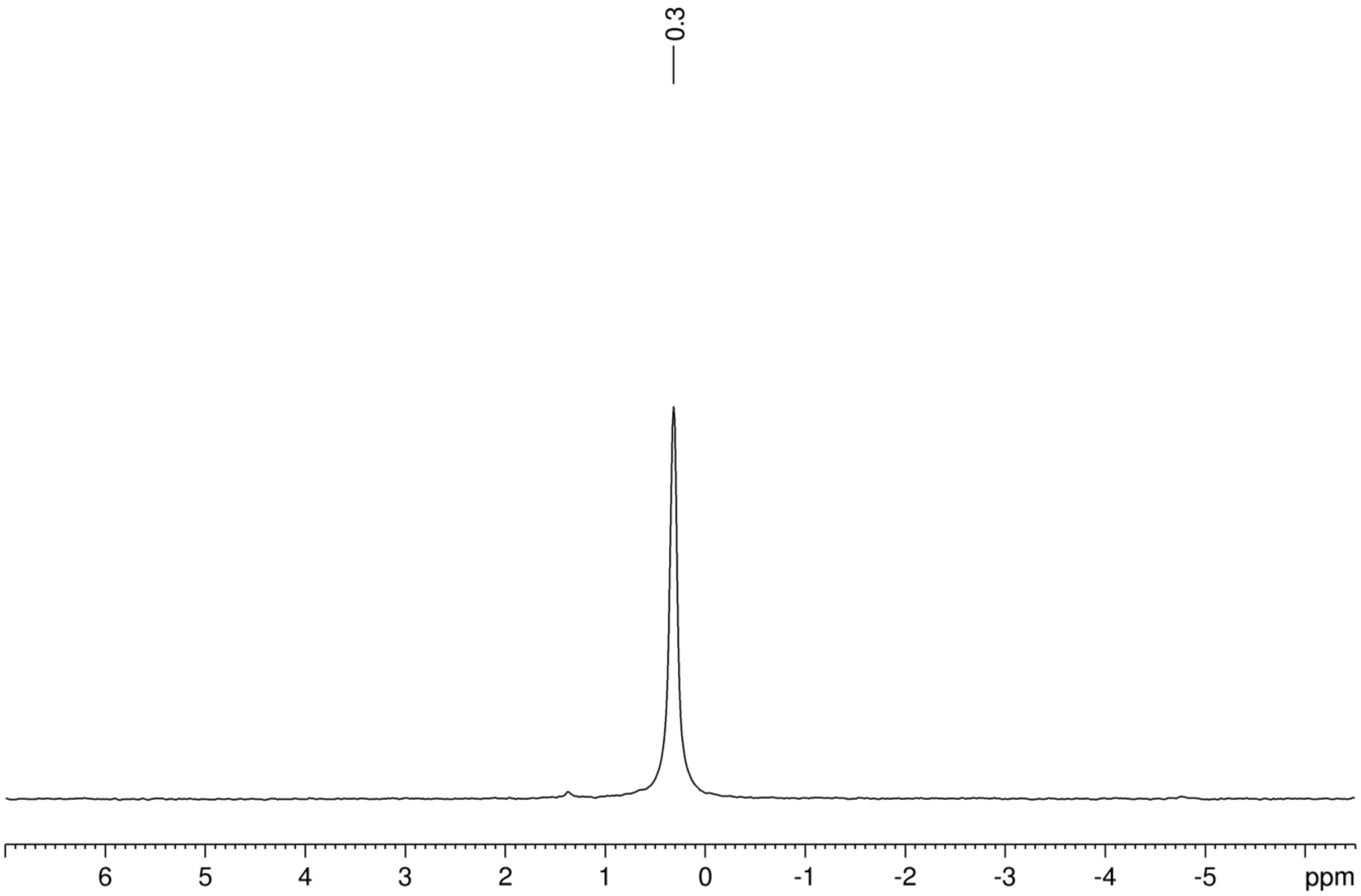

Figure S23. *$^{31}P\{^{1}H\}$ NMR (125 MHz, CD_2Cl_2, −80 °C). Complex **8**.*

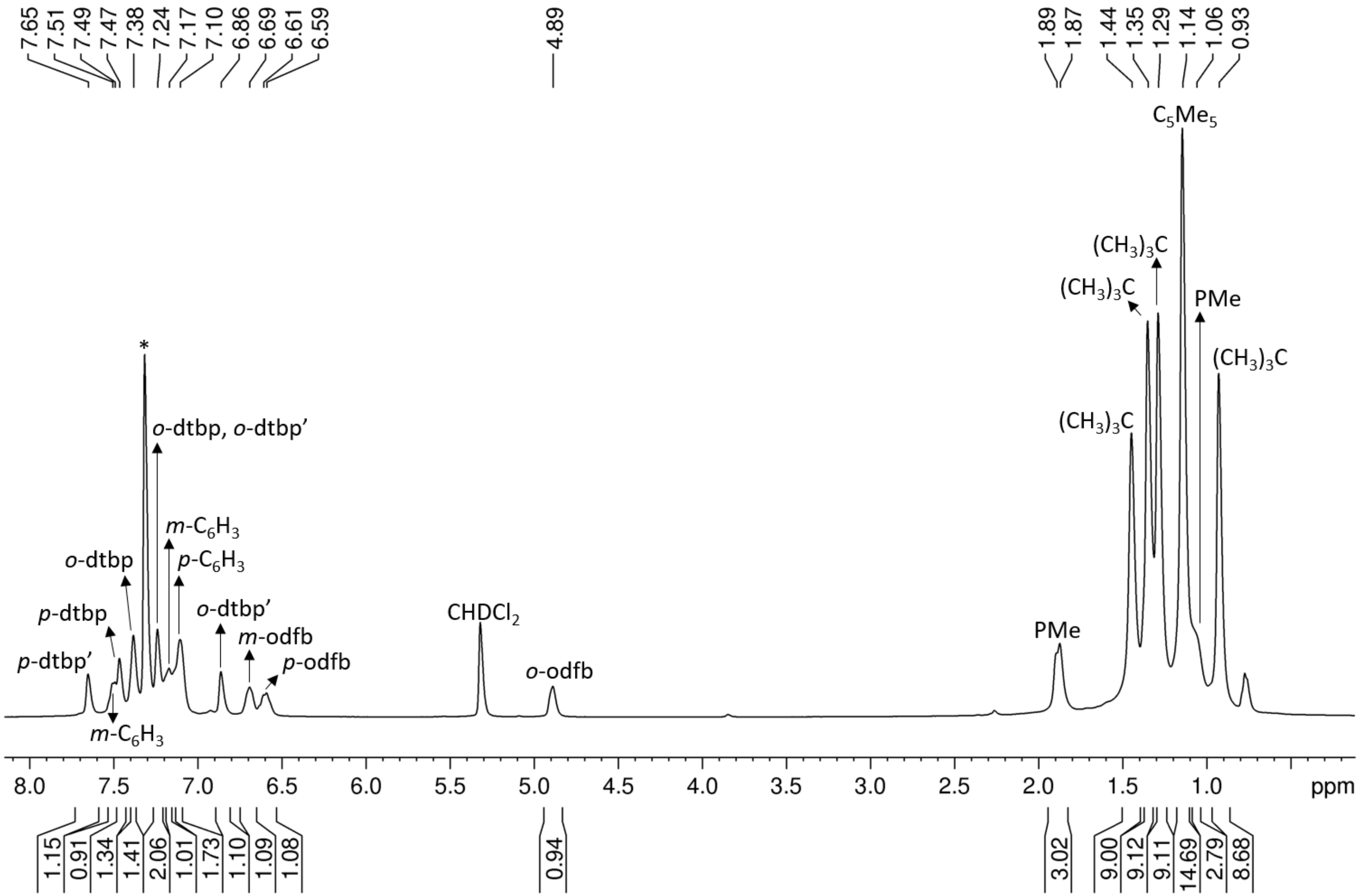

Figure S24. *^{1}H NMR (400 MHz, CD_2Cl_2, −80 °C). Complex **8**.*

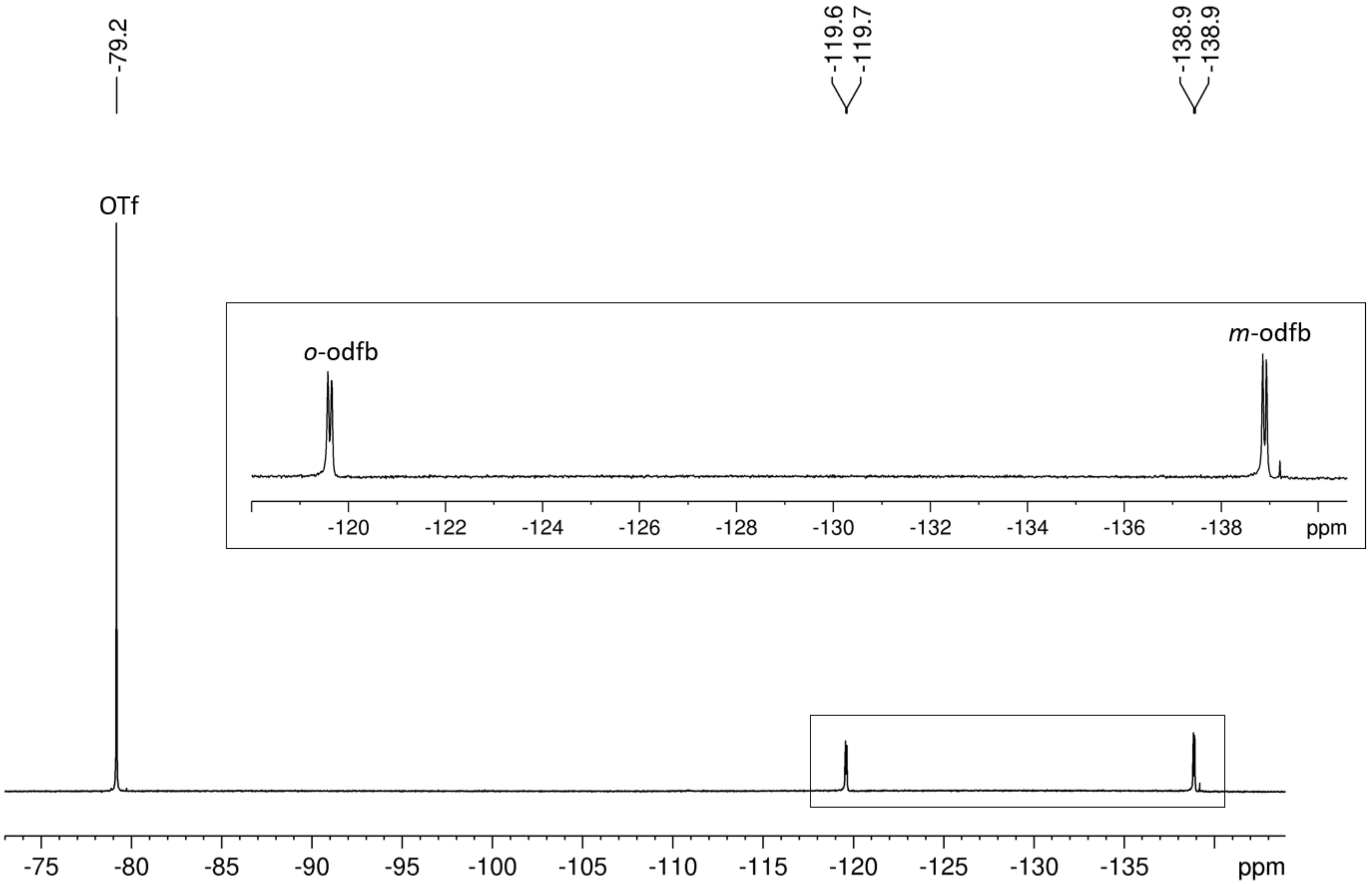

Figure S25. *$^{19}F\{^{1}H\}$ NMR (376 MHz, CD_2Cl_2, −80 °C). Complex **8**.*

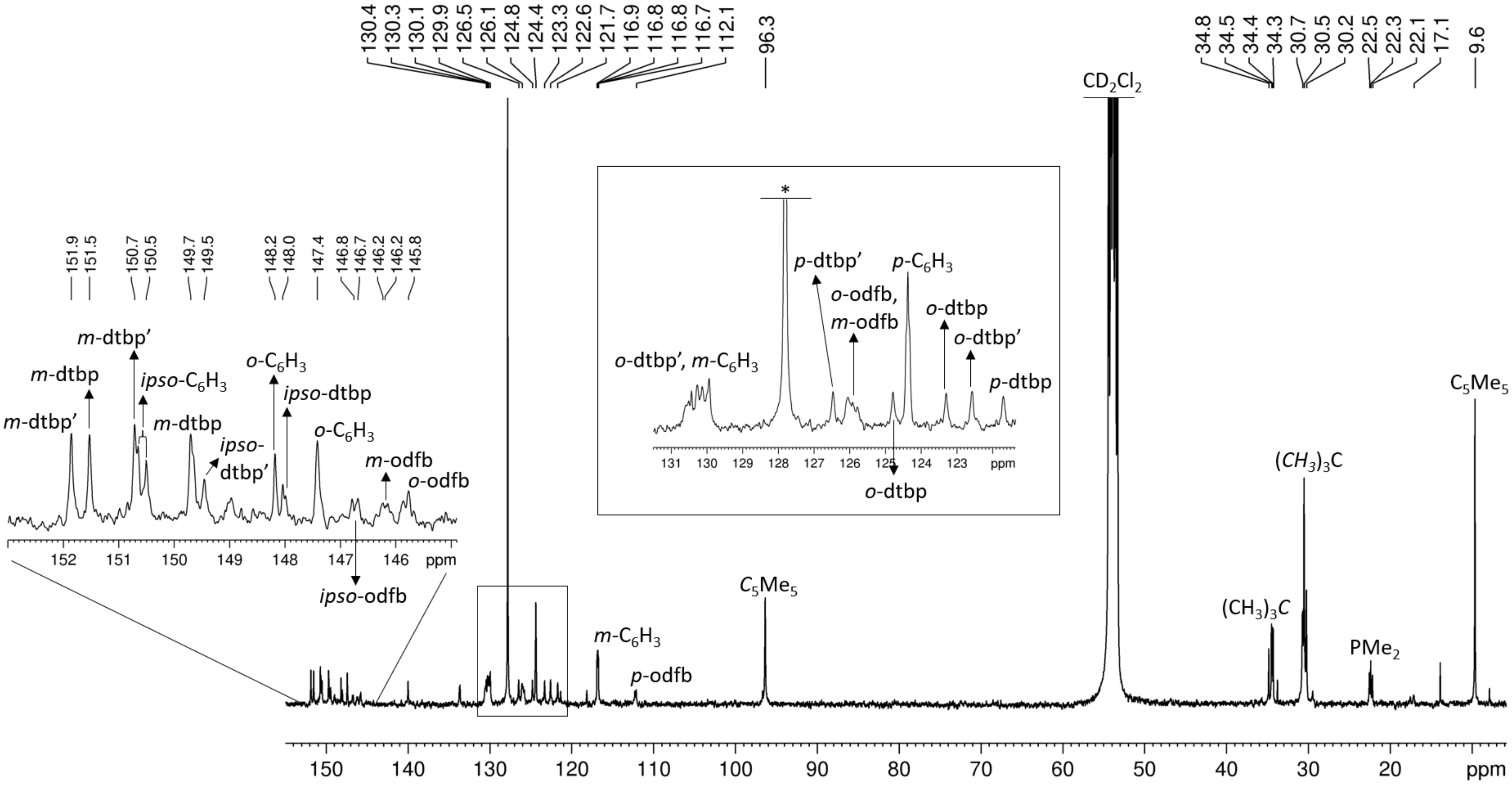

Figure S26. $^{13}C\{^1H\}$ *NMR (100 MHz, CD_2Cl_2, −80 °C). Complex **8**.*

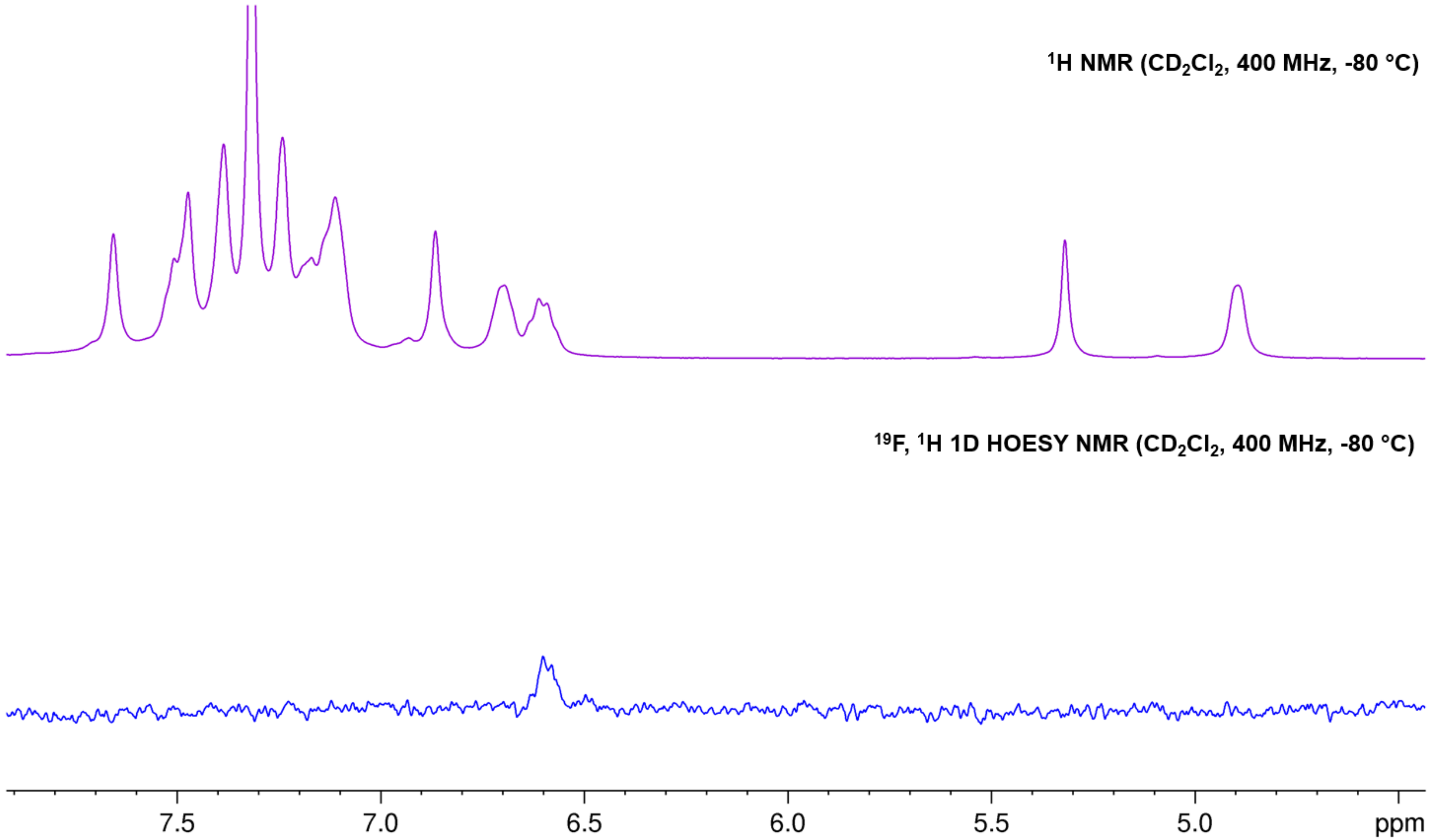

Figure S27. *Stacked ^{1}H NMR (400 MHz, CD_2Cl_2, −80 °C) spectrum of complex **8** (top) and monodimensional ^{1}H, ^{19}F HOESY NMR (400 MHz, CD_2Cl_2, −80 °C) obtained by selectively irradiating the fluorine resonance centered at -138.9 ppm (down).*

3’

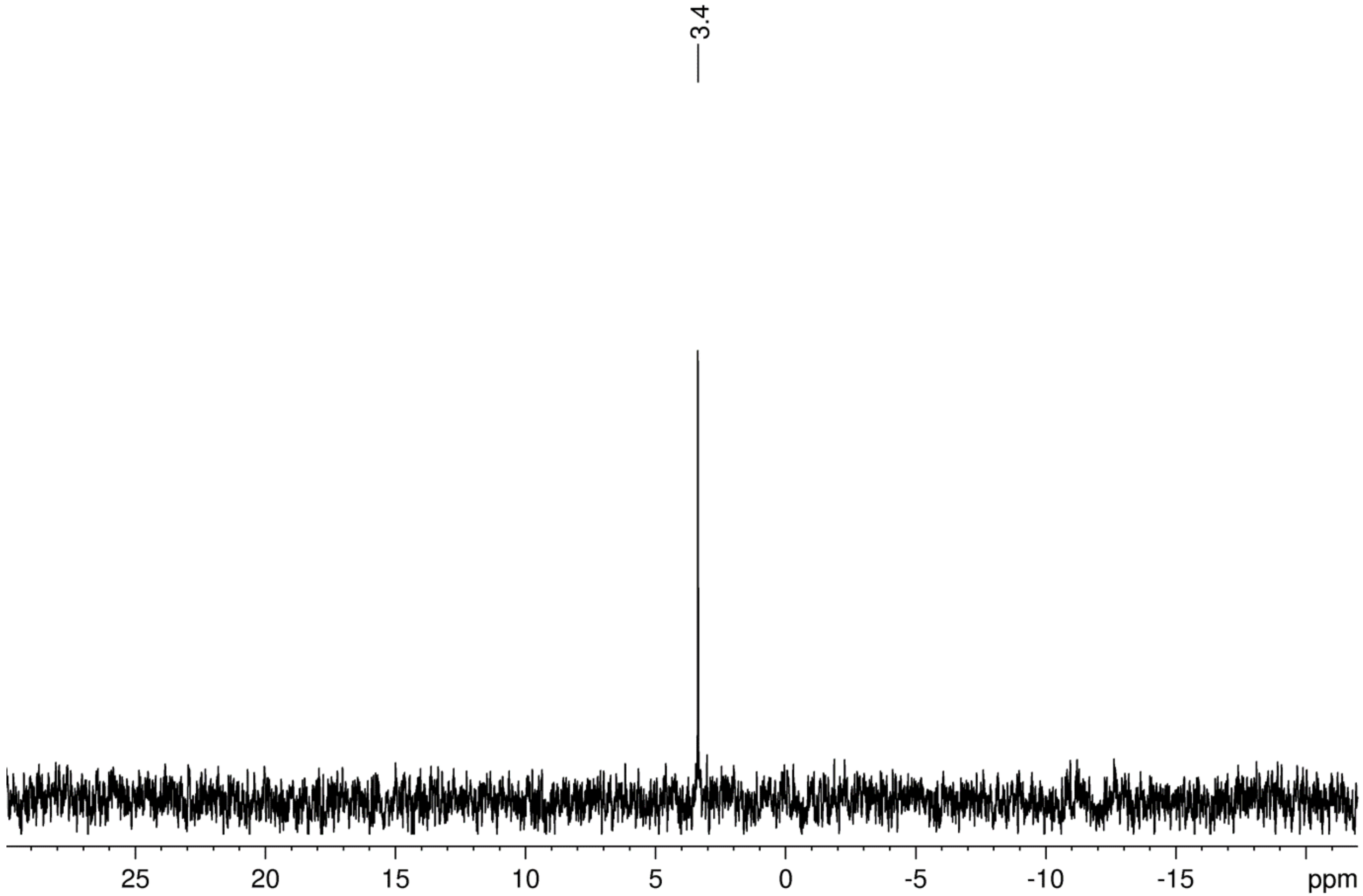

Figure S28. *$^{31}P\{^1H\}$ NMR (125 MHz, CD_2Cl_2, 25 °C). Complex* ***3’****.*

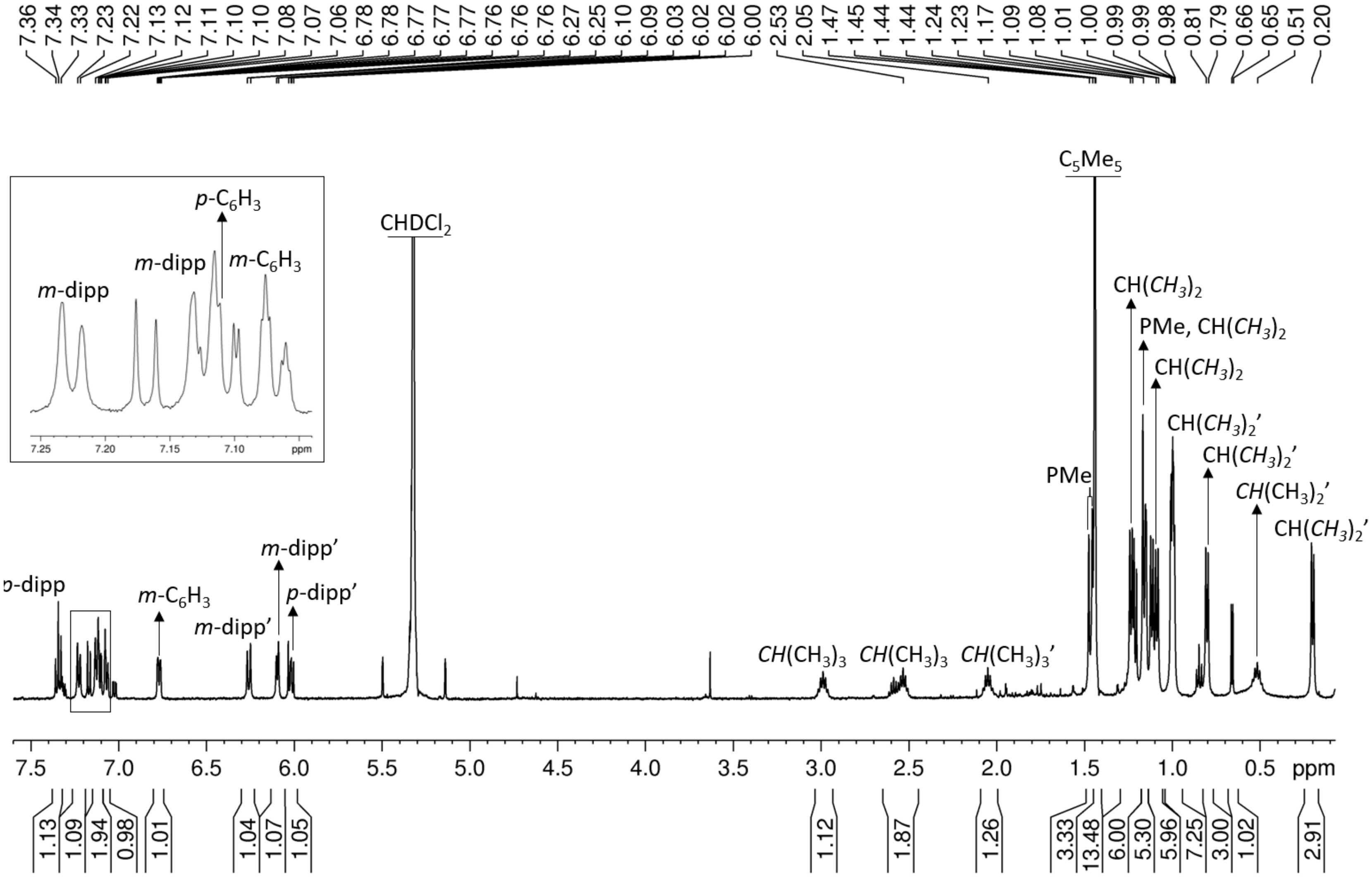

Figure S29. *^{1}H NMR (500 MHz, CD2Cl2, 25 °C). Complex **3’***.

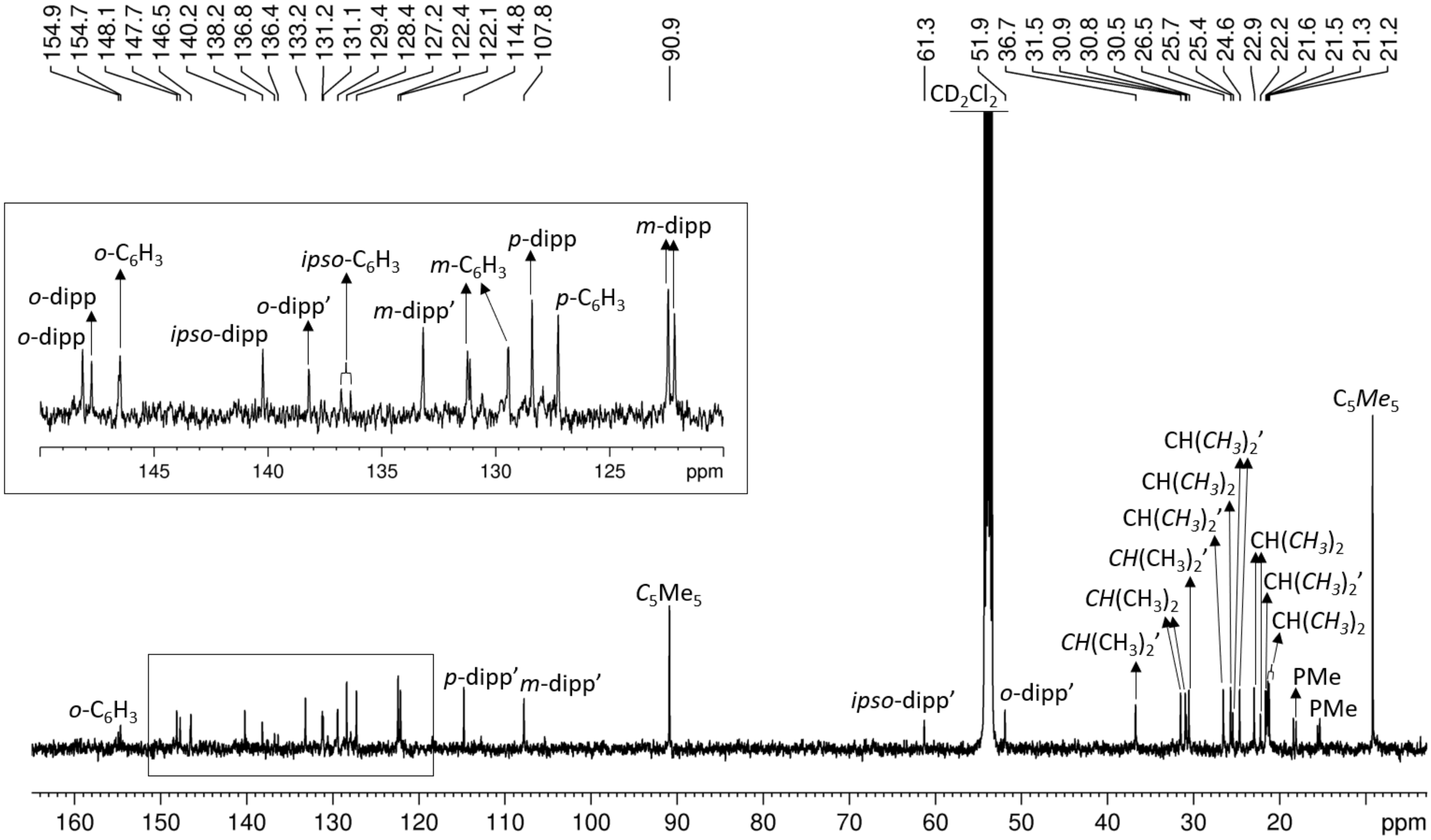

Figure S30. *$^{13}C\{^1H\}$ NMR (100 MHz, CD_2Cl_2, 25 °C). Complex* ***3'***

2. X-Ray Structural Characterization of complexes

A summary of the fundamental crystal and refinement data are given in **Table S1** and **Table S2**. Atomic coordinates, anisotropic displacement parameters and bond lengths and angles can be found in the cif files, which have been deposited in the Cambridge Crystallographic Data Centre with no 2539429, 2539430, 2537874, 2537872 and 2537869. These data can be obtained free of charge from The Cambridge Crystallographic Data Centre via www.ccdc.cam.ac.uk/data_request/cif.

Table S1. *Crystal data and structure refinement*

	**1**	**2**	**3**
Formula	$C_{46}H_{66}IrCl_2P$	$C_{82}H_{85}IrBClF_{24}P$	$C_{46}H_{66}IrP$
Fw	913.05	2044.07	842.15
Crystal size, mm	0.27 x 0.23 x 0.21	0.21 x 0.20 x 0.12	0.42 x 0.36 x 0.115
Crystal system	Monoclinic	Triclinic	Triclinic
Space group	$P2_1/n$	P-1	P-1
a, Å	8.9003(5)	11.5369(8)	11.4649(4)
b, Å	21.6775(13)	18.8325(11)	12.2086(5)
c, Å	22.5359(13)	22.2864(15)	15.3137(5)
α, deg	90	112.911(2)	95.4630(10)
β, deg	90.566(3)	91.621(3)	104.238(2)
γ, deg	90	98.988(3)	92.853(2)
V, Å^3	4347.8(4)	4384.2(5)	2062.23(13)
T, K	273.15	173.15	193
Z	4	2	2
ρ_{calc}, g cm^{-3}	1.395	1.548	1.356
μ, mm^{-1}	1.770	1.014	3.305
Radiation	AgKα (λ = 0.56086)	AgKα (λ = 0.56086)	MoKα (λ = 0.71073)
F(000)	1872	2052	868.0
Absorption corrections	multi-scan 0.621-0.745	multi-scan 0.664-0.745	multi-scan 0.498-0.746
θ range, deg	2.26-22.01	2.36-21.96	2.00-28.31
no. of rflns meads	64649	134378	77509
R_{int}	0.0469	0.0528	0.0918
no. of rflns unique	10749	21888	10258
no. of params / restraints	486/0	1092/12	483/0
R_1 ($I > 2\sigma(I)$) [a]	0.0377	0.0423	0.0318
R_1 (all data)	0.0469	0.0505	0.0425
wR_2 ($I > 2\sigma(I)$)	0.0755	0.1073	0.0729
wR_2 (all data)	0.0778	0.1126	0.0799
Diff. Fourier peaks min/max, eÅ^{-3}	-3.78/2.17	-2.10/2.79	-1.94/1.90
CCDC number	2539429	2539430	2537874

Table S2. *Crystal data and structure refinement*

	**3'**	**8**
Formula	$C_{42}H_{58}IrP$	$C_{62}H_{78}F_5IrO_3PS$
Fw	786.05	1221.47
cryst.size, mm	0.20 x 0.12 x 0.10	0.24 x 0.18 x 0.075
crystal system	Monoclinic	Triclinic
space group	$P2_1/c$	P-1
a, Å	11.4711(5)	10.2631(9)
b, Å	16.4971(8)	15.6693(13)
c, Å	19.6300(7)	19.8486(18)
α, deg	90	105.049(3)
β, deg	98.4350(10)	98.999(3)
γ, deg	90	106.558(3)
V, Å^3	3674.6(3)	2861.7(4)
T, K	193	193
Z	4	2
ρ_{calc}, g cm^{-3}	1.421	1.418
μ, mm^{-1}	3.704	2.457
Radiation	MoKα (λ = 0.71073)	MoKα (λ = 0.71073)
F(000)	1608.0	1254.0
Absorption corrections	multi-scan<br>0.582-0.746	multi-scan<br>0.562-0.744
θ range, deg	2.18-28-37	2.07-26.43
no. of rflns measd	70974	63516
R_{int}	0.0539	0.0934
no. of rflns unique	9120	11758
no. of params / restraints	412/0	709/0
R_1 ($I > 2\sigma(I)$) [a]	0.0335	0.0352
R_1 (all data)	0.0518	0.0510
wR_2 ($I > 2\sigma(I)$)	0.0772	0.0724
wR_2 (all data)	0.0925	0.0783
Diff. Fourier peaks min/max, eÅ^{-3}	-1.61/2.19	-1.18/1.00
CCDC number	2537872	2537869

3. Computational Chemistry

Calculations were performed at the DFT level with the Gaussian 09 (Revision E.01) program.[1,2] The hybrid functional PBE0[3] was used throughout all computational studies. Dispersion effects were accounted for by using Grimme's D3 parameter set with Becke−Johnson (BJ) damping.[4] Geometry optimizations were carried out without geometry constraints, using the 6-31G(d,p)[5-7] basis set to represent the C, H, P, Cl, O, Mg, F, and S atoms and the Stuttgart/Dresden Effective Core Potential and its associated basis set (SDD)[8] to describe the Ir atom. Bulk solvent effects (dichloromethane or diethylether) were included at the optimization stage with the SMD continuum model.[9] The stationary points and their nature as minima or saddle points (TS) were characterized by vibrational analysis, which also produced enthalpy (H), entropy (S) and Gibbs energy (G) data at 298.15 K. The minima connected by a given transition state were determined by perturbing the transition states along the TS coordinate and optimizing to the nearest minimum. QTAIM analyses were performed with the Multiwfn[10] software on wavefunctions generated with the Gaussian 09 program. Energy Decomposition calculations have been performed with the ADF program.[11,12]

An Activation Strain Analysis is a fragment-based approach, in which the relative energy of a molecular complex along the reaction energy profile can be broken into to two contributing factors: (i) the energy needed to deform the reactants from their optimum geometry into the geometry required to react: ΔE^{strain}, and II the interaction energy, previously described, between the deformed reactants: ΔE^{int}. This method provides the user with insight into the factors that govern the height of the reaction.[13]

3.1 Formation of 5 via oxidative addition

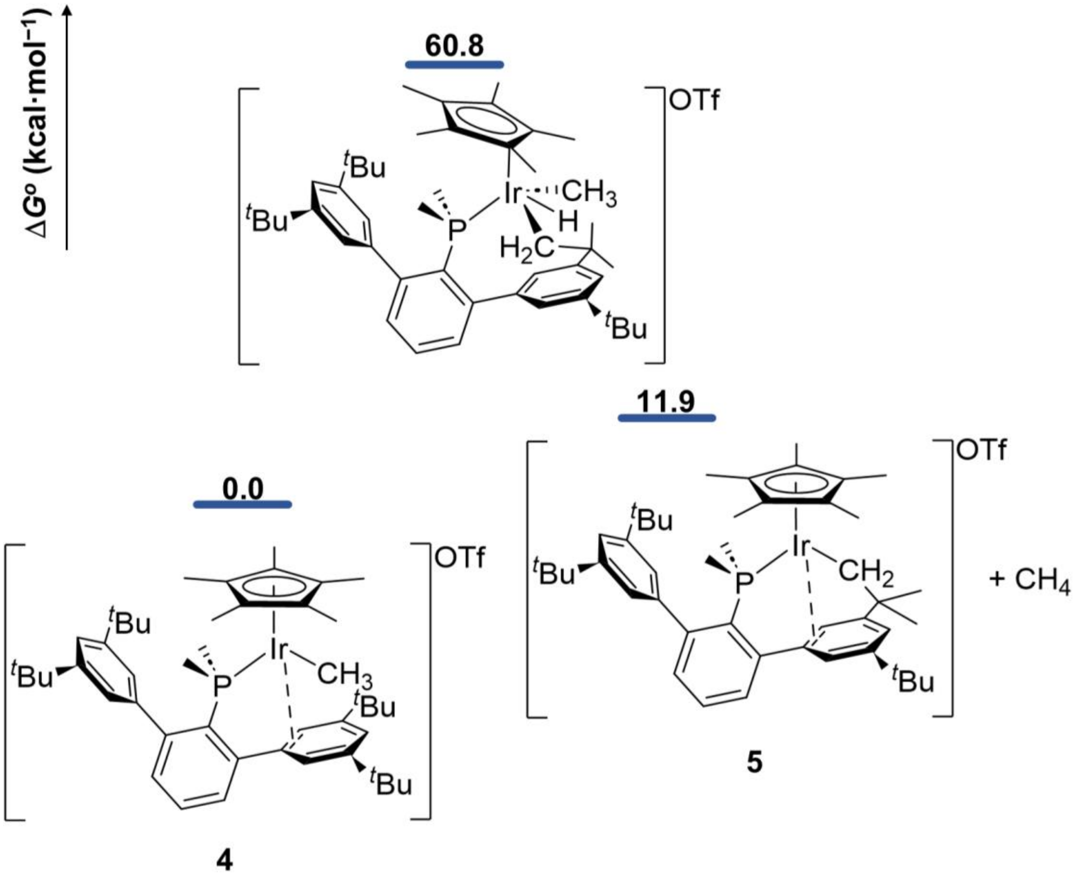

Figure S31. *Free energy of the intermediate in the oxidative addition pathway leading to **5**.*

3.2 Elimination of CH_4 from 4 via formation of a fulvene species

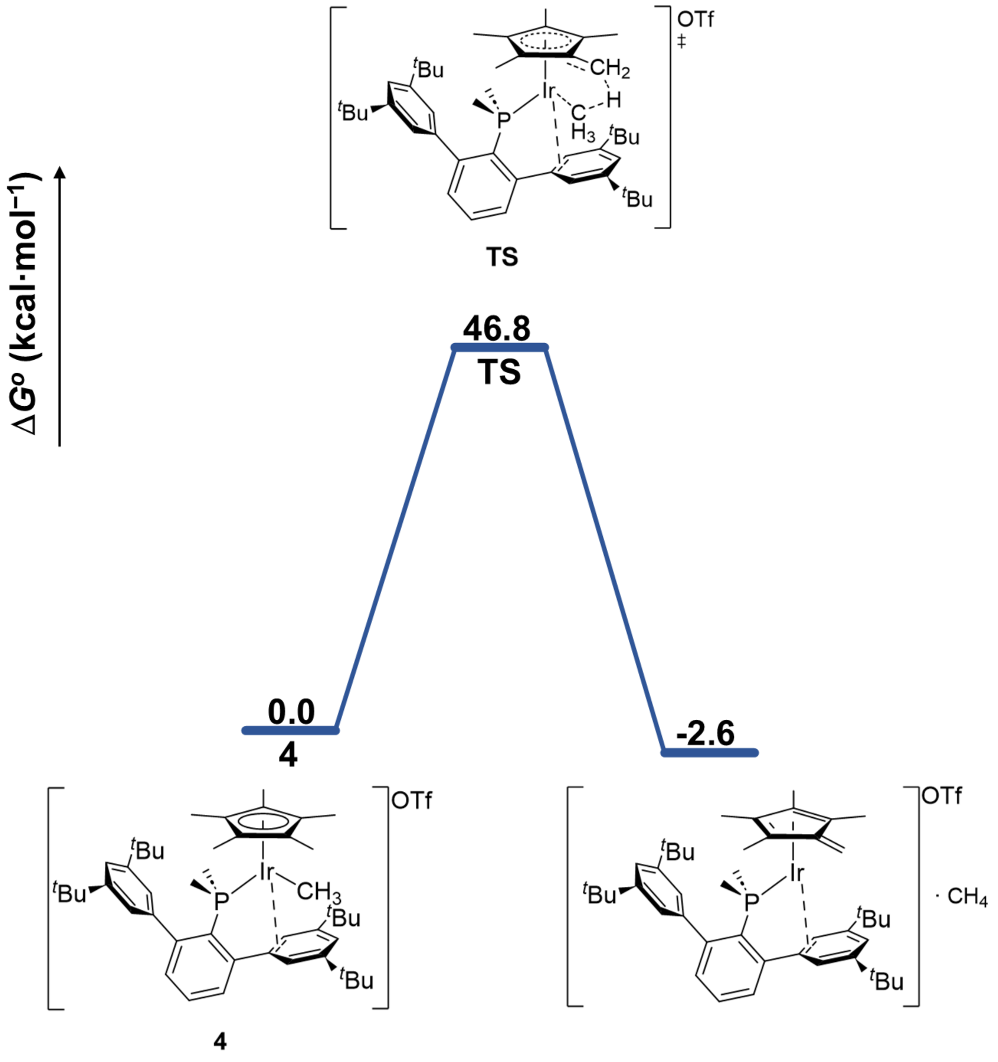

Figure S32. *Free energy profile for the elimination of CH_4 from **4** by forming a fulvene species.*

3.3 Formation of 7

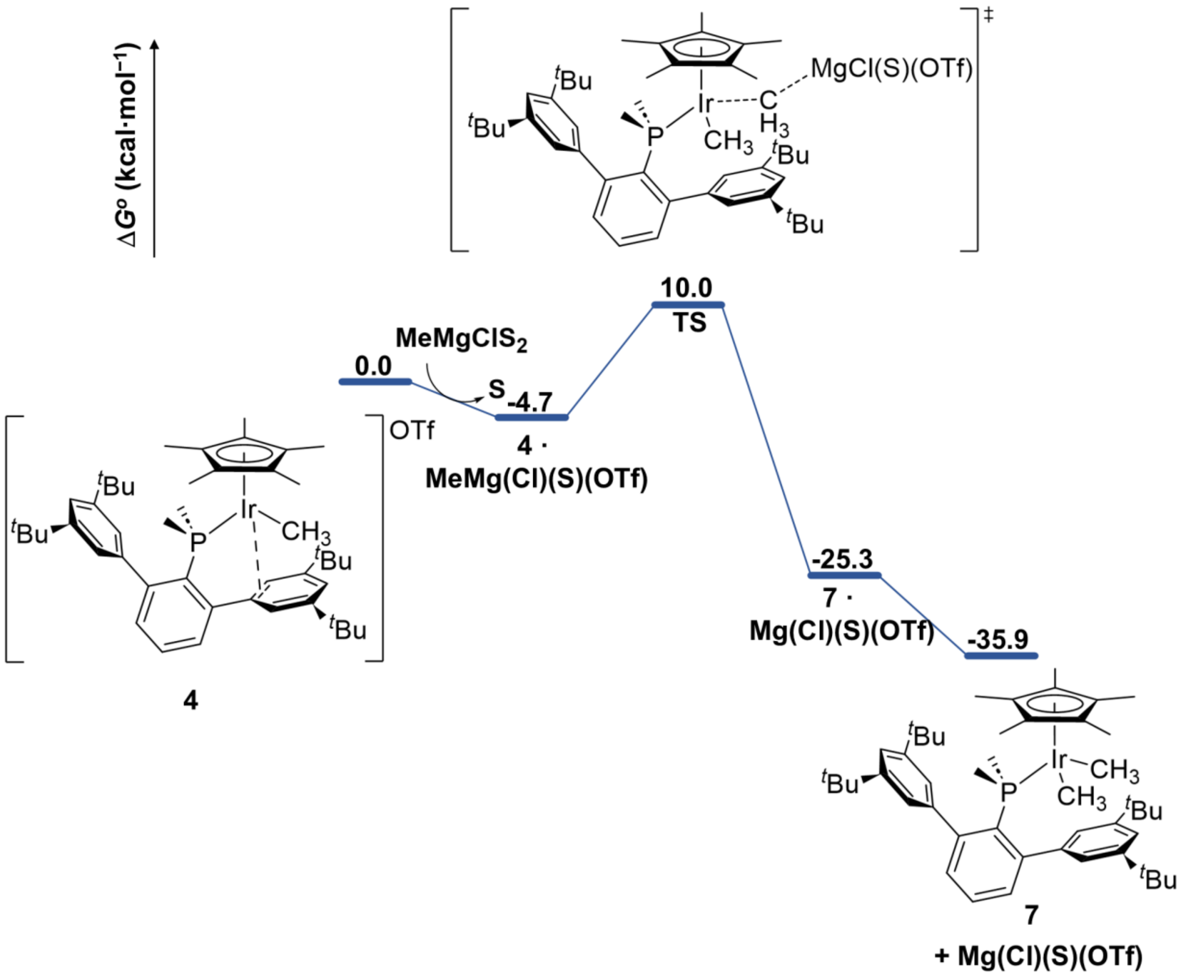

Figure S33. *Free energy profile for the formation of 7 from* ***4*** *and MeMgClS$_2$. Explicit solvent molecules (Me$_2$O) included in the calculations are represented as S.*

3.4 Elimination of ethane from 7

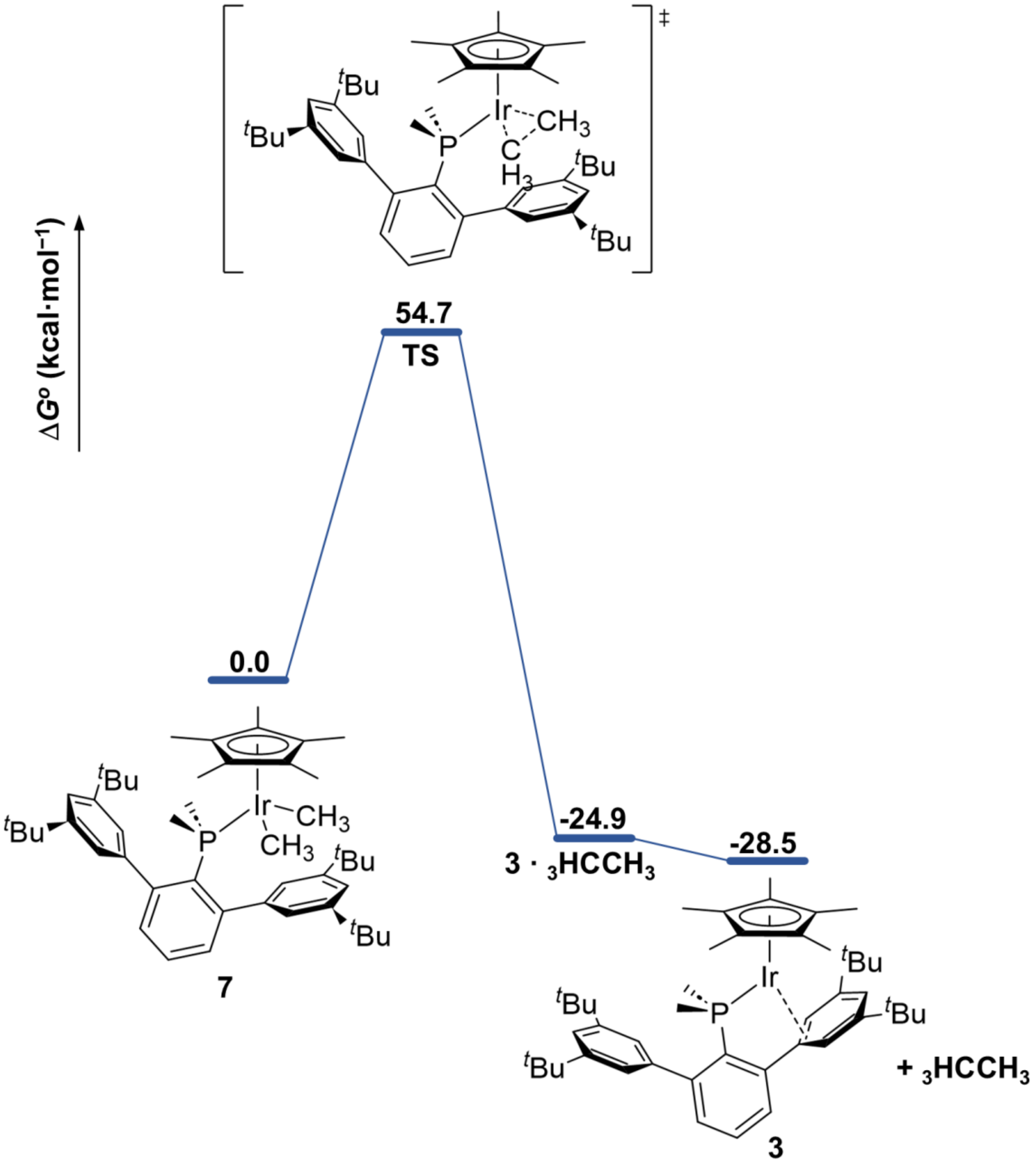

Figure S34. *Free energy profile for the elimination of ethane from* **7**.

3.5 Formation of 6 from 4

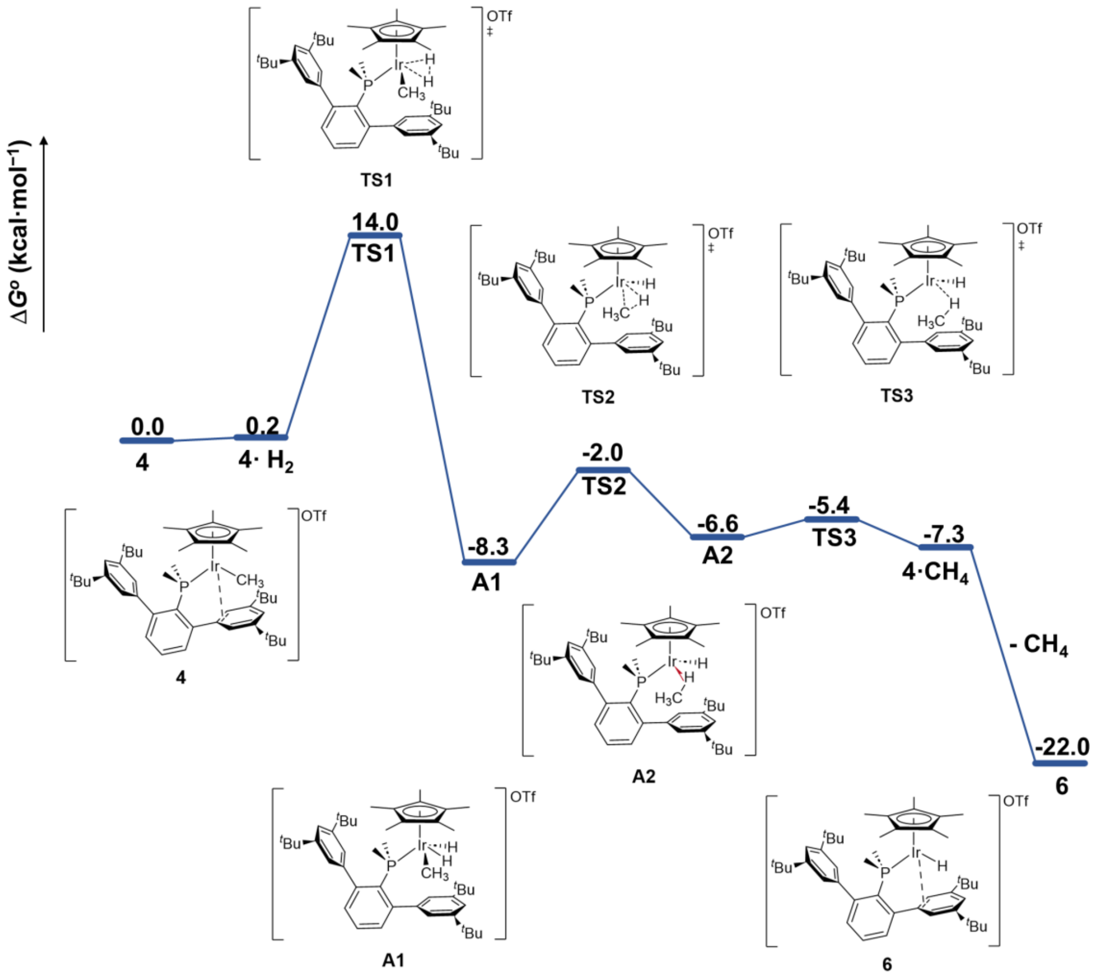

Figure S35. *Free energy profile for the formation of **6** from **4** and H_2.*

3.6 Formation of 6 from 5

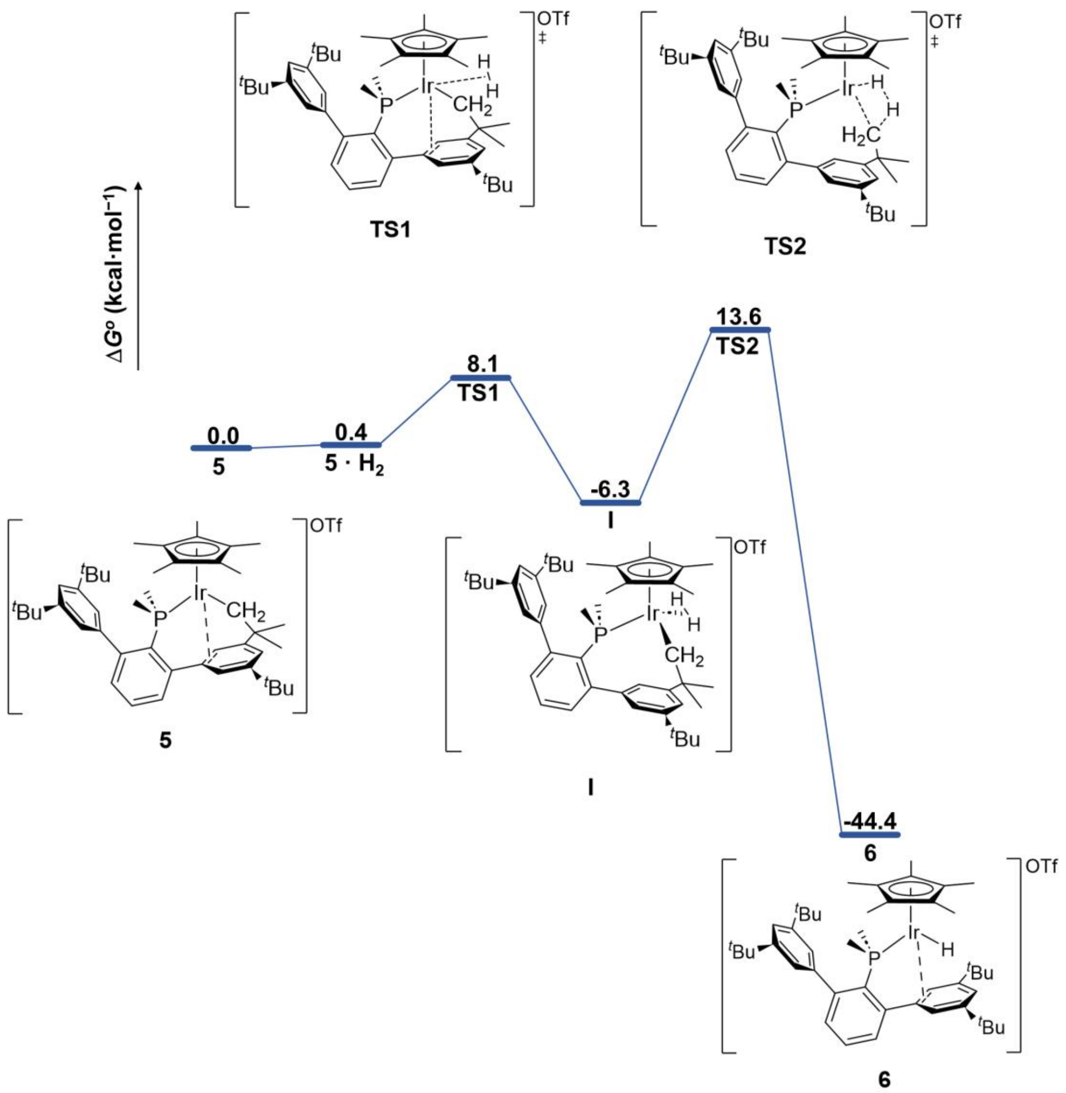

Figure S36. *Free energy profile for the formation of* ***6*** *from* ***5*** *and* H_2.

3.7 Activation Strain Analysis of the formation of Ir-Me species

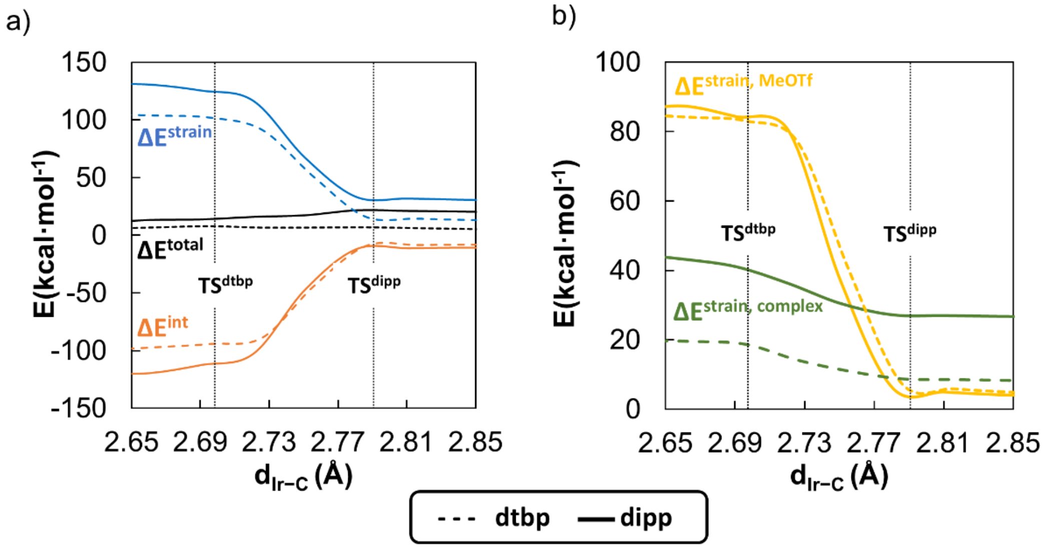

Figure S37*. Activation strain analyses of the electrophilic attack of MeOTf to* ***3’*** *(solid lines) or* ***3*** *(dashed lines) projected onto the Ir-C bond-forming distance. a) Comparative activation strain analyses; b) Comparative strain energies of the [Ir] and MeOTf fragments. The Ir–C distance at which the transition states take place is indicated with dotted vertical lines. All data have been computed at the ZORA-BP86-D3/TZ2P//PBE0-D3(SMD=DCM)/SDD(Ir)/6-31(d,p)(all other atoms) level.*

3.8 Interaction energy and torsion angle correlation

The strength of that interaction between the metal center and the arene rings. can be inferred from the torsion of the interacting aryl ring relative to the central aryl ring of the phosphine. We wondered whether we could correlate the geometric parameters of the compounds with the strength of the interaction between the metal center and the arene ring. In this context, we plotted the torsion angle given by the X-Ray structures and the interaction energy between the phosphine and the rest of the metal complex (see Figure S40), obtained by a Energy Decomposition Analysis. The more the torsion angle deviates from 180º, lower (more intense) is the interaction energy, as it could be expected.

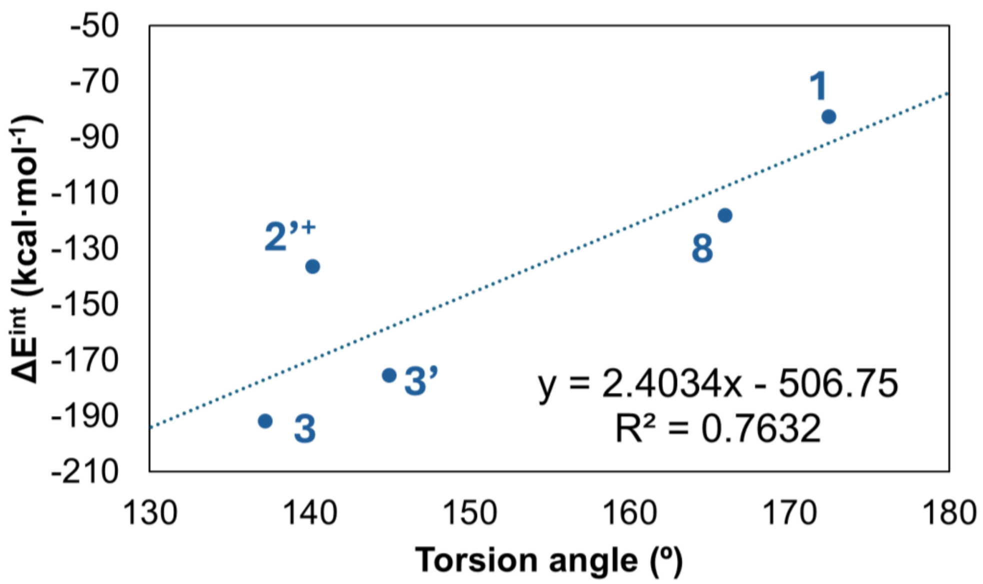

Figure S38. *Plot of the interaction energy (ΔE^{int}) between the phosphine ligand and the rest of the complex versus the torsion angle of the lateral ring. In each case, the ring considered is the one that exhibits the greatest deviation from 180º. All data have been computed at the ZORA-BP86-D3/TZ2P//PBE0-D3(SMD=DCM)/SDD(Ir)/6-31(d,p)(all other atoms) level.*

3.9 Topological studies

The electron density value at a bond critical point (ρ) is often associated with the bond strength. The Laplacian of the electron density at this point ($\nabla^2\rho$) is negative in covalent bonds, indicating an accumulation of electron density, whereas it is positive in ionic or closed-shell interactions, which signifies a depletion of electron density. Additionally, the total energy density (H) is generally positive for ionic bonds and negative for covalent bonds. The nature of an interaction can also be inferred from the ratio of local Potential (V) to Kinetic (G) energies. For covalent bonds, the ratio $|V|/G$ is greater than 2, while for ionic interactions, it is typically less than 1. Intermediate interactions, such as metal-metal or metal-ligand bonds, often exhibit a positive $\nabla^2\rho$, near-zero H, and a $|V|/G$ ratio between 1 and 2.[14,15]

In all figures in this section the solid and dashed lines correspond to positive and negative values of $\nabla^2\rho$ respectively. In-plane bcps and bond paths of the electron density are superimposed.

Topological analysis offers a complementary perspective on the nature of the metal-ligand interaction. Previously, we observed that complex **2’** featured only a single bcp between the metal center and any of the carbon atoms of the lateral aryl ring, namely a bcp between

the Ir and C_{ipso} atom, was found.[16] In contrast, complexes **3'** and **3** feature both a bcp involving the Ir and the C_{ipso} and a bcp involving the Ir and the C_{ortho} of one of the lateral rings (Figure S41 and Figure S42). Both bcp exhibit parameters consistent with a metal-ligand interaction (see first paragraph of this section and Tables S3 and S4).

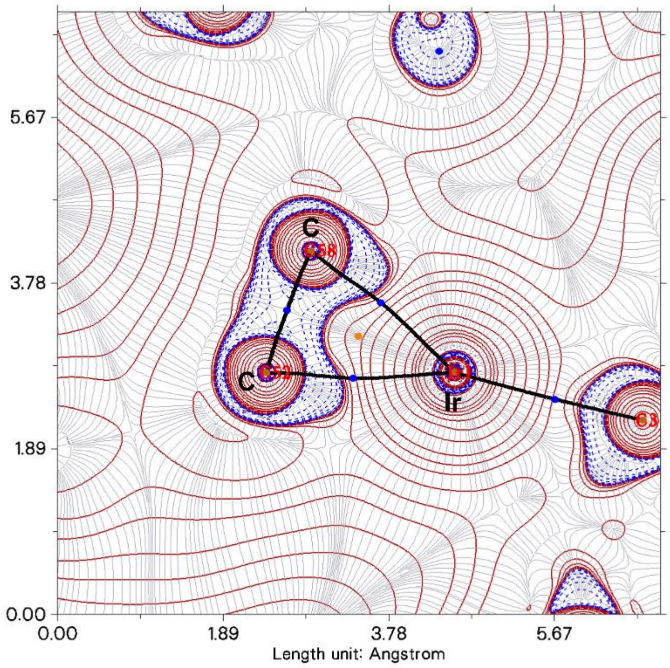

Figure S39. *Plot of the laplacian of the electron density,* $\nabla^2\rho$, *of complex* ***3*** *in the plane containing the Ir,* C_{ipso}, *and one of the* C_{ortho} *atoms. Both C atoms belong to the dtbp group which features an interaction with the metal center.*

Table S3. *Selected properties of the electron density at relevant bcps shown in* ***Figure S37***

bond	ρ^b	G_a^c	V_a^c	H_a^c	$\lvert V_a \rvert / G_a$	$\nabla^2\rho^e$
**Ir–C*ipso***	0.10744	0.07928	– 0.12337	– 0.04409	1.5561	0.14669
**Ir–C*ortho***	0.10572	0.07937	– 0.12189	– 0.04252	1.5357	0.15328
**C–C**	0.26248	0.08132	– 0.30878	– 0.22746	3.7971	– 0.58457

[a] average values, [b] $e \cdot bohr^{-3}$, [c] Hartree, [d] $e \cdot bohr^{-5}$, e = elementary charge.

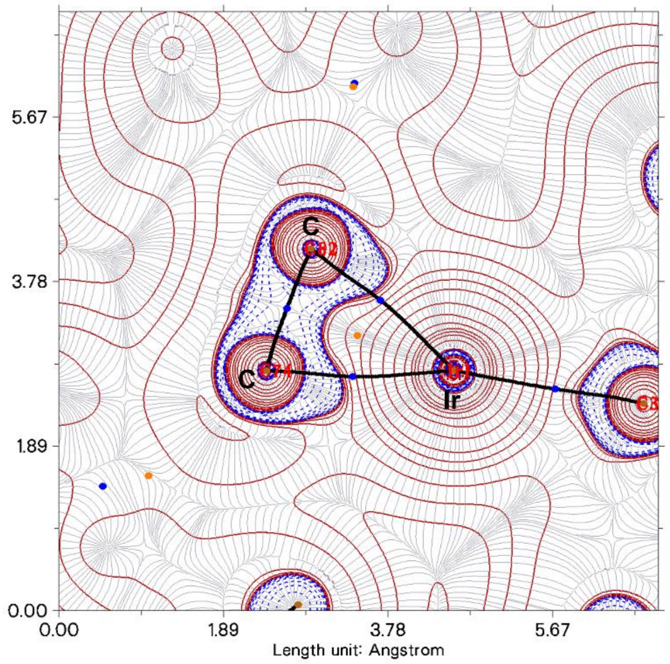

***Figure S40**. Plot of the laplacian of the electron density, $\nabla^2\rho$, of complex **3'** in the plane containing the Ir, C_{ipso}, and one of the C_{ortho} atoms. Both C atoms belong to the dipp group which features an interaction with the metal center.*

Table S4**. Selected properties of the electron density at relevant bcps shown in **Figure S38

bond	ρ^b	$G_a{}^c$	$V_a{}^c$	$H_a{}^c$	$\lvert V_a \rvert / G_a$	$\nabla^2\rho^e$
**Ir–C_{ipso}**	0.10576	– 0.07828	– 0.12108	– 0.04279	1.5471	0.14775
**Ir–C_{ortho}**	0.10600	– 0.07603	– 0.11915	– 0.04311	1.5671	0.13711
**C–C**	0.25574	0.07921	– 0.29560	– 0.2163	3.7318	– 0.54865

[a] average values, [b] $e\cdot bohr^{-3}$, [c] Hartree, [d] $e\cdot bohr^{-5}$, e = elementary charge.

Intermediate **A2** of the mechanism depicted in Figure S37 was characterized topologically (Figure S43 and Table S4) showing the presence of a bcp connecting the hydrogen and iridium atoms. This bcp features parameters expected for a metal-ligand interaction, making A2 a methane sigma complex.

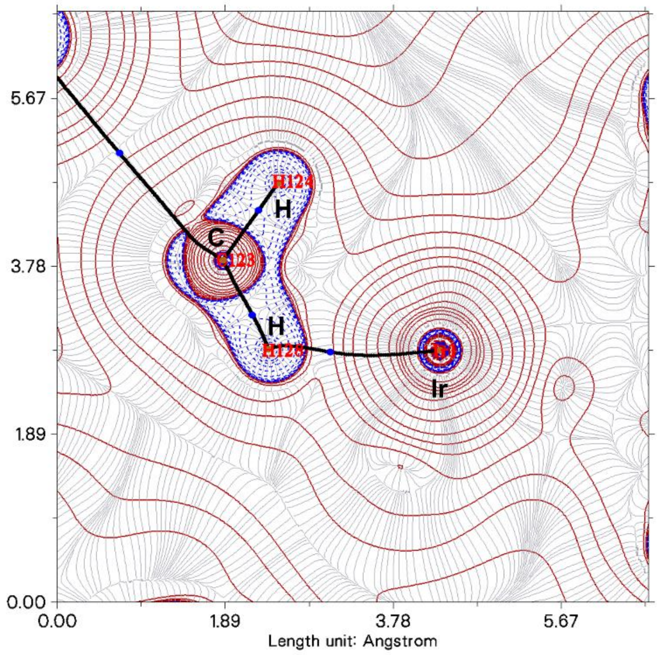

Figure S41. *Plot of the laplacian of the electron density, $\nabla^2\rho$, of intermediate **A2** of the energy profile of the formation of **6** from **4** and H_2 in the plane containing the Ir atom and the H and C atoms of the forming CH_4.*

Table S5. *Selected properties of the electron density at relevant bcps shown in **Figure S39***

bond	ρ^b	G_a^c	V_a^c	H_a^c	$\lvert V_a \rvert / G_a$	$\nabla^2\rho^e$
**Ir–H**	0.05947	0.05455	– 0.06975	– 0.01520	1.2786	0.15998
**H–C**	0.23567	0.05021	– 0.26489	– 0.21468	5.2756	– 0.65786

[a] average values, [b] e·bohr^{-3}, [c] Hartree, [d] e·bohr^{-5} , e = elementary charge.

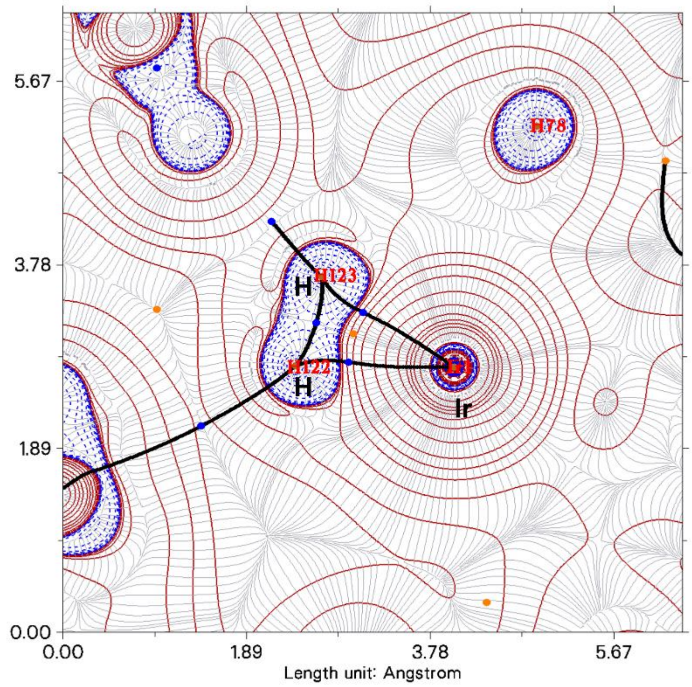

Figure S42. *Plot of the laplacian of the electron density, $\nabla^2\rho$, of intermediate **A1** of the energy profile of the formation of **6** from **5** and H_2 in the plane containing the Ir atom and the two H atoms.*

Table S6. *Selected properties of the electron density at relevant bcps shown in **Figure S40***

bond	ρ^b	G_a^c	V_a^c	H_a^c	$\lvert V_a \rvert / G_a$	$\nabla^2\rho^e$
**Ir–H**	0.13407	0.12243	– 0.18444	– 0.06201	1.5064	0.25544
**Ir–H**	0.13219	0.11497	– 0.17569	– 0.06071	1.5281	0.13219
**H–H**	0.30670	0.09978	– 0.40409	– 0.30430	4.0498	– 0.81810

[a] average values, [b] $e\cdot bohr^{-3}$, [c] Hartree, [d] $e\cdot bohr^{-5}$, e = elementary charge.

4. References

1. Gaussian 09, Revision E.01, M. J. Frisch, G. W. Trucks, H. B. Schlegel, G. E. Scuseria, M. A. Robb, J. R. Cheeseman, G. Scalmani, V. Barone, G. A. Petersson, H. Nakatsuji, X. Li, M. Caricato, A. Marenich, J. Bloino, B. G. Janesko, R. Gomperts, B. Mennucci, H. P. Hratchian, J. V. Ortiz, A. F. Izmaylov, J. L. Sonnenberg, D. Williams-Young, F. Ding, F. Lipparini, F. Egidi, J. Goings, B. Peng, A. Petrone, T. Henderson, D. Ranasinghe, V. G. Zakrzewski, J. Gao, N. Rega, G. Zheng, W. Liang, M. Hada, M. Ehara, K. Toyota, R. Fukuda, J. Hasegawa, M. Ishida, T. Nakajima, Y. Honda, O. Kitao, H. Nakai, T. Vreven, K. Throssell, J. A. Montgomery, Jr., J. E. Peralta, F. Ogliaro, M. Bearpark, J. J. Heyd, E. Brothers, K. N. Kudin, V. N. Staroverov, T. Keith, R. Kobayashi, J. Normand, K. Raghavachari, A. Rendell, J. C. Burant, S. S. Iyengar, J. Tomasi, M. Cossi, J. M. Millam, M. Klene, C. Adamo, R. Cammi, J. W. Ochterski, R. L. Martin, K. Morokuma, O. Farkas, J. B. Foresman, and D. J. Fox, Gaussian, Inc., Wallingford CT, **2016**.
2. Perdew, J. P.; Burke, K.; Ernzerhof, M. Generalized Gradient Approximation Made Simple. *Phys. Rev. Lett.* **1996**, 77 (18), 3865-3869. DOI: 10.1103/PhysRevLett.77.3865
3. Grimme, S.; Antony, J.; Ehrlich, S.; Krieg, H. A Consistent and Accurate Ab Initio Parametrization of Density Functional Dispersion Correction (DFT-D) for the 94 Elements H-Pu. *J. Chem.Phys.* **2010**, 132 (15). DOI: 10.1063/1.3382344.
4. Hariharan, P. C.; Pople, J. A. The Influence of Polarization Functions on Molecular Orbital Hydrogenation Energies. *Theor. Chim. Acta* **1973**, 28 (3), 213-222. DOI: 10.1007/BF00533485
5. Francl, M. M.; Pietro, W. J.; Hehre, W. J.; Binkley, J. S.; Gordon, M. S.; DeFrees, D. J.; Pople, J. A. Self-Consistent Molecular Orbital Methods. XXIII. A Polarization-Type Basis Set for Second-Row Elements. *J. Chem. Phys.* **1982**, 77 (7), 3654–3665. DOI: 10.1063/1.444267.
6. Hehre, W. J.; Ditchfield, R.; Pople, J. A. Self—Consistent Molecular Orbital Methods. XII. Further Extensions of Gaussian—Type Basis Sets for Use in Molecular Orbital Studies of Organic Molecules. *J. Chem. Phys.* **1972**, 56 (5), 2257–2261. DOI: 10.1063/1.1677527
7. Andrae, D.; Häußermann, U.; Dolg, M.; Stoll, H.; Preuß, H. Energy-Adjusted *ab Initio* Pseudopotentials for the Second and Third Row Transition Elements. *Theor. Chim. Acta* **1990**, 77 (2), 123–141. DOI: 10.1007/BF01114537
8. Marenich, A. v.; Cramer, C. J.; Truhlar, D. G. Universal Solvation Model Based on Solute Electron Density and on a Continuum Model of the Solvent Defined by the Bulk Dielectric Constant and Atomic Surface Tensions. *J. Phys. Chem. B* **2009**, 113 (18), 6378-6396. DOI: 10.1021/jp810292n
9. Ribeiro, R. F.; Marenich, A. v.; Cramer, C. J.; Truhlar, D. G. Use of Solution-Phase Vibrational Frequencies in Continuum Models for the Free Energy of Solvation. *J. Phys. Chemistry B* **2011**, 115 (49), 14556-14562. DOI: 10.1021/jp205508z
10. Lu, T.; Chen, F. Multiwfn: A Multifunctional Wavefunction Analyzer. *J. Comput. Chem.* **2012**, 33 (5), 580–592. DOI: 10.1002/jcc.22885.
11. G. te Velde, F.M. Bickelhaupt, E.J. Baerends, C. Fonseca Guerra, S.J.A. van Gisbergen, J.G. Snijders and T. Ziegler, Chemistry with ADF. J. Comp. Chem. **2001**, 22, 931-967. DOI: 10.1002/jcc.1056
12. ADF 2021.104, SCM, Theoretical Chemistry, Vrije Universiteit, Amsterdam, The Netherlands, http://www.scm.com.
13. Curado, N.; Carrasco, M.; Álvarez, E.; Maya, C.; Peloso, R.; Rodríguez, A.; López-Serrano, J.; Carmona, E. Lithium Di- and Trimethyl Dimolybdenum(II) Complexes with Mo-Mo Quadruple Bonds and Bridging Methyl Groups. *J. Am. Chem. Soc.* **2015**, 137 (38), 12378-12387. DOI: 10.1021/jacs.5b07899
14. Varadwaj, P. R.; Varadwaj, A.; Marques, H. M. DFT-B3LYP, NPA-, and QTAIM-Based Study of the Physical Properties of [M(II)(H_2O)$_2$(15-Crown-5)] (M = Mn, Fe, Co, Ni, Cu, Zn) Complexes. *J. Phys. Chem. A* **2011**, 115 (22), 5592-5601. DOI: 10.1021/jp2001157
15. Varadwaj, P. R.; Cukrowski, I.; Marques, H. M. DFT-UX3LYP Studies on the Coordination Chemistry of Ni2+. Part 1: Six Coordinate $[Ni(NH_3)_n(H_2O)_{6-n}]^{2+}$ Complexes. *J. Phys. Chem. A* **2008**, 112 (42), 10657-10666. DOI: 10.1021/jp803961s
16. Pita-Milleiro, A.; Alférez, M. G.; Moreno, J. J.; Espada, M. F.; Maya, C; Campos, J. *Inorg. Chem.* **2023**, 62 (15), 5961-5971. DOI: 10.1021/acs.inorgchem.2c04381
